# Beyond Biomimetics: Pathology‐Informed Engineering Rescues Reconstituted HDL From Inflammatory Dysfunction for Sepsis Immunotherapy

**DOI:** 10.1002/advs.77714

**Published:** 2026-09-10

**Authors:** Han Zhou, Lei Zhang, Xingyu Cai, Jiajia Su, Guangyu Dai, Xue Chen, Hanming Wang, Jia Hua, Jianping Liu, Chunmeng Sun, Xiancheng Chen, Erhao Wang, Wenli Zhang

**Affiliations:** ^1^ Department of Pharmaceutics China Pharmaceutical University Nanjing Jiangsu People's Republic of China; ^2^ Department of pharmacy Hainan Women and Children’s Medical Center Haikou Hainan People's Republic of China; ^3^ Department of Critical Care Medicine Nanjing Drum Tower Hospital, The Affiliated Hospital of Nanjing University Medical School Nanjing Jiangsu People's Republic of China

**Keywords:** cerium nanozyme, drug delivery system, dysfunction, immunotherapy, reconstituted high‐density lipoprotein, sepsis

## Abstract

Biomimetic therapies hold great promise for treating intractable diseases, yet their translation is often hindered by dysfunction—the loss of intended biological function upon exposure to pathological microenvironments. This challenge is well exemplified by reconstituted high‐density lipoprotein (rHDL), a clinically advanced HDL mimetic and representative biomimetic drug delivery platform. Here, guided by a pathology‐informed identification‐design‐evaluation‐validation workflow, we identified representative sepsis‐relevant dysfunction liabilities of HDL‐based systems under pathology‐relevant conditions and designed an adaptive engineered guard for inflammatory stress (AEGIS) strategy to rescue rHDL function. AEGIS integrates triphenylphosphine‐modified ceria nanozyme‐celastrol coordination complex (TCe–C) into rHDL to form TCe‐C@rHDL, enabling carrier protection, decoupling disease‐driven damage from dysfunction, and preserving HDL‐intrinsic routing for hierarchical cytoplasmic and mitochondrial delivery. Across multiple evaluation models, including inflammatory biochemical challenges, patient serum under shear flow, and inflammatory animal settings, TCe‐C@rHDL consistently preserved biomimetic function better than a clinical‐stage CER‐001 mimetic. This performance was further validated by therapeutic efficacy in vivo, restoring 90% survival in LPS‐induced systemic inflammation versus 40% for the CER‐001 mimetic, and improving survival to 80% in the CLP model when combined with antibiotics versus 40% for antibiotics alone. Collectively, this work establishes a paradigm for next‐generation biomimetic systems that remain functional under pathological stress.

## Graphical abstract

A pathology‐informed engineering strategy rescues reconstituted HDL from inflammatory dysfunction in sepsis. By integrating a ceria nanozyme‐celastrol coordination complex, the platform preserves HDL structure and endotoxin handling, sustains hierarchical intracellular delivery, and enhances immunoregulation across pathological models, translating functional protection into markedly improved survival in endotoxin‐driven and polymicrobial sepsis.

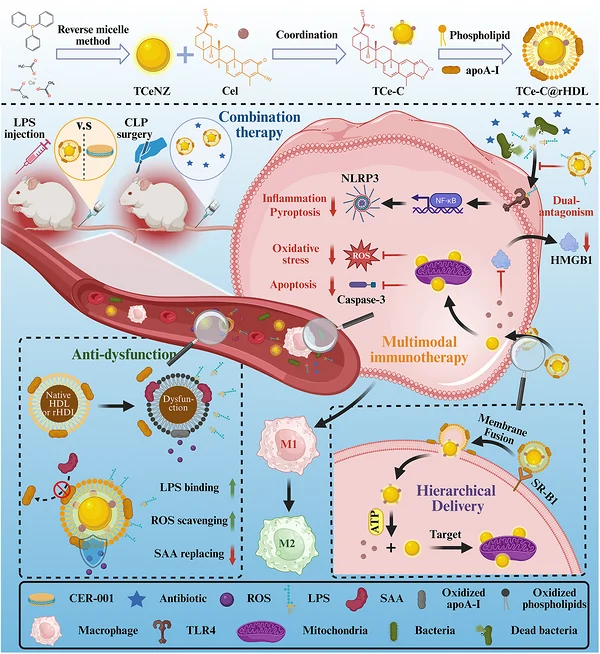

## 1 Introduction

Biomimetic therapeutics, including cell membrane‐coated nanoparticles, lipoprotein‐based nanoplatforms, virus‐like particles, and rapidly expanding live‐cell therapies, have emerged as promising drug delivery platforms for treating intractable diseases [1, 2, 3, 4, 5]. By replicating or harnessing endogenous structures, these systems are expected to inherit the biocompatibility, targeting ability, and biological functions of their natural counterparts [6, 7]. However, one critical question remains largely overlooked: can biomimetic functions be maintained after these systems enter pathological microenvironments?

This question is central to the translational future of biomimetic medicine. In reality, pathological environments are rich in reactive oxygen species (ROS), inflammatory mediators, and competing biomolecules. These factors can remodel biomimetic carriers, thereby reducing targeting precision, accelerating clearance, and even turning beneficial features into liabilities [8, 9, 10]. This pathological transformation is defined as dysfunction, a state where biomimetic functions no longer perform as intended due to pathological microenvironmental interference. However, such vulnerabilities are often masked when evaluations rely on simplified buffers, healthy serum, or other in vitro and in vivo models that poorly reflect the pathological milieu, leading to misjudge the biomimetic performance under pathological conditions [11, 12]. Drawing conceptual inspiration from quality by design (QbD) in pharmaceutical development [13], biomimetic performance should not be defined solely by end‐point testing, but should instead be considered prospectively through pathology‐informed design and screening, with continuous validation in disease‐relevant models.

High‐density lipoprotein (HDL) provides an instructive example. HDL is a multifunctional plasma complex mainly composed of apolipoprotein A‐I (apoA‐I), phospholipids and regulatory factors [14]. Beyond its well‐established role in cholesterol transport, HDL also functions as an endogenous immunomodulator in inflammatory disorders such as sepsis [15, 16]. HDL can sequester lipopolysaccharide (LPS) and facilitate its detoxification through apoA‐I‐ and scavenger receptor class B type 1 (SR‐B1)‐associated processes, thereby attenuating systemic inflammation [17, 18]. On this basis, reconstituted HDL (rHDL), exemplified by the clinical candidate CER‐001, has shown promise in Phase IIa trials of sepsis [19]. Besides, inspired by SR‐B1‐mediated selective uptake, the natural pathway directly delivering lipids into the cytoplasm, as well as its intrinsic immunomodulatory and macrophage‐targeting properties [20, 21], rHDL has also been harnessed as a versatile biomimetic delivery nanoplatform for inflammatory diseases [22, 23, 24]. Despite this promise, clinical experience suggests that rHDL may be less equipped to withstand pathological remodeling, given that native HDL (nHDL) itself undergoes dysfunction under inflammatory stress. For example, the phase III failure of CSL‐112 [25], another clinically advanced rHDL formulation, together with the still limited therapeutic validation of CER‐001 in inflammatory settings [26], have been attributed in part to inflammation‐associated structural and functional remodeling. In fact, the absence in rHDL of endogenous regulators present in nHDL, such as paraoxonase‐1 and lipopolysaccharide transfer protein, renders it more susceptible to oxidative damage and impaired LPS‐neutralization capacity. In parallel, serum amyloid A (SAA), which increases more than 100‐fold during acute inflammation, can more readily displace apoA‐I as the dominant apolipoprotein in rHDL. This diminishes its immunomodulatory efficacy, targeting precision, and circulating time [27, 28, 29, 30].

To address this problem, we propose an “anti‐dysfunction” design concept: a biomimetic carrier should not merely imitate physiology at baseline, but preserve and rescue its function under pathology. In our preliminary studies, strategies such as preserving endogenous paraoxonase‐1 or introducing small‐molecule antioxidants achieved only limited immunoactivity and short‐lived benefits [31, 32]. This led us to reason that overcoming dysfunction requires a broader strategy: beyond protecting the carrier itself, the pathological microenvironment must be interrupted to prevent continued carrier damage, while the preserved HDL‐like route should be exploited for more potent bioactivity; importantly, conceptually aligned with QbD, such a strategy should be designed and screened from the outset using pathology‐relevant models to improve in vitro–in vivo correlation.

**SCHEME 1:**
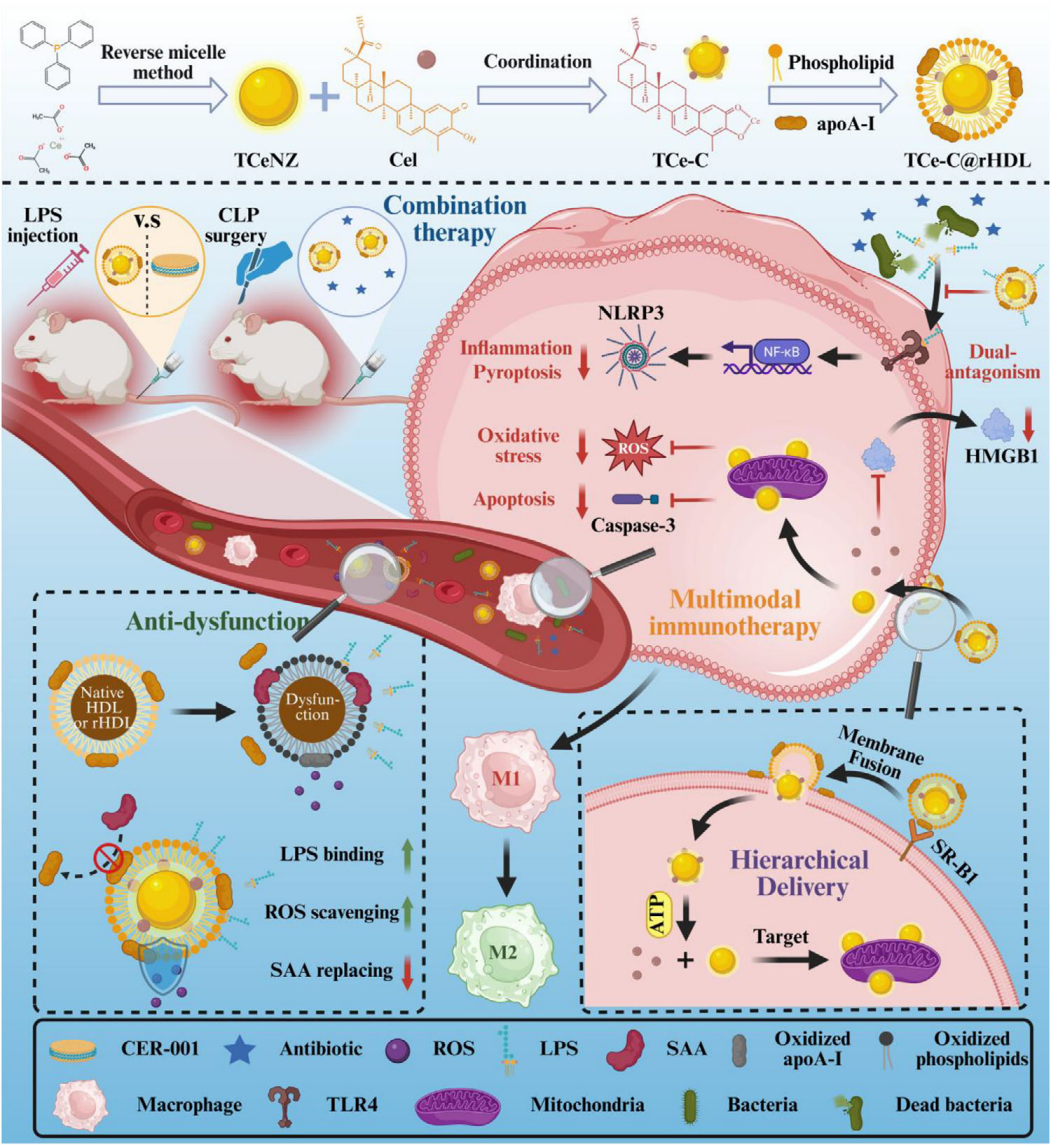
Anti‐dysfunction TCe‐C@rHDL for sepsis immunotherapy. TCeNZ was synthesized by the reverse micelle method and coordinated with Cel to form the responsive TCe–C complex, which was then incorporated into rHDL to generate TCe‐C@rHDL. Guided by a pathology‐informed workflow, HDL‐based dysfunction liabilities under inflammatory stress were first defined and then addressed through the AEGIS strategy. Compared with native HDL or the clinical‐stage rHDL mimetic, TCe‐C@rHDL exhibits improved resistance to inflammatory dysfunction, including enhanced ROS scavenging, preserved LPS capture, and reduced SAA‐mediated apoA‐I displacement. After SR‐B1‐mediated membrane fusion, intracellular ATP triggers dissociation of the TCe–C complex, enabling cytoplasmic release of Cel for HMGB1‐directed immunomodulation and mitochondrial accumulation of TCeNZ for oxidative‐stress intervention. Through this hierarchical delivery process, TCe‐C@rHDL promotes dual antagonism of LPS and HMGB1, attenuation of oxidative stress, pyroptosis‐associated inflammatory signaling, and apoptosis, and reprogramming of macrophages toward an immunoregulatory phenotype. Therapeutic efficacy was further evaluated against the CER‐001 mimetic in the LPS‐induced systemic inflammation model and in combination with antibiotics in the clinically relevant cecal ligation and puncture (CLP) model.

Based on this rationale, we first identified representative sepsis‐relevant dysfunction liabilities of HDL‐based systems by benchmarking nHDL and clinical‐stage rHDL mimetics under simulated inflammatory conditions. Guided by these identified dysfunction liabilities, we then developed an adaptive engineered guard for inflammatory stress (AEGIS) strategy for rHDL (Scheme 1). AEGIS centers on the incorporation of a triphenylphosphine‐modified ceria nanozyme‐celastrol coordination complex (TCe‐C) into rHDL. In this construct, triphenylphosphine (TPP)‐modified ceria nanozyme (TCeNZ) provides sustained ROS‐scavenging activity [33, 34], while its cationic TPP moiety modulates surface charge relevant to LPS binding and resistance to SAA‐mediated apoA‐I displacement [35, 36, 37]. Celastrol (Cel), a natural compound with HMGB1‐antagonistic and anti‐inflammatory activities [38, 39], was introduced to interrupt the LPS/HMGB1‐driven amplification of oxidative and inflammatory stress, thereby decoupling disease‐driven microenvironmental damage from carrier dysfunction. Meanwhile, the responsive coordination between TCeNZ and Cel preserves the intrinsic HDL routing advantage: following cellular delivery, the ATP‐responsive TCe–C coordination is expected to facilitate intracellular component separation, enabling cytoplasmic availability of Cel and TPP‐guided mitochondrial accumulation of TCeNZ. We then evaluated whether AEGIS could preserve rHDL function across progressively more stringent and disease‐relevant models, including oxidative and inflammatory biochemical challenges, patient serum under relevant shear flow, inflammatory pharmacokinetic and biodistribution settings, and cellular assays of uptake, intracellular routing, LPS clearance, and immunoregulation. Finally, transcriptomic analysis, an LPS‐induced systemic inflammation model, and an infection‐associated cecal ligation and puncture (CLP) sepsis model were used to evaluate whether the preserved anti‐dysfunction phenotype translated into therapeutic activity across controlled endotoxin‐driven inflammation and polymicrobial sepsis.

## 2 Methods

### 2.1 Cell Culture, Animals, and Ethics Statement

RAW264.7 cells (ATCC: TIB‐71) were cultured in DMEM medium supplemented with 10% (v/v) fetal bovine serum (FBS) and 1% (v/v) penicillin‐streptomycin (P/S) at 37°C in a humidified atmosphere containing 5% CO_2_.

ICR mice (20–25 g) and Sprague–Dawley (SD) rats (180–220 g) were purchased from Qinglongshan Biotechnology Co., Ltd. (Nanjing, China). All animals were acclimated to standard conditions at the Experimental Animal Centre of China Pharmaceutical University. All experimental procedures were approved by the Animal Ethical Experimentation Committee of China Pharmaceutical University (Approval No. YSL‐202506114).

The collection and use of serum samples from patients with endotoxemia were approved by the Medical Ethics Committee of Nanjing Drum Tower Hospital (Approval No. 2025‐0577‐03). All procedures involving human samples were conducted in accordance with the approved protocols and relevant ethical guidelines.

### 2.2 Evaluation and Validation of the Anti‐Dysfunction Properties of TCe‐C@rHDL

#### 2.2.1 Antioxidant Enzyme‐Mimetic Activities

The SOD‐ and CAT‐like activities of rHDL (blank reconstituted HDL), Cel@rHDL (rHDL encapsulating Cel only), TCe@rHDL (rHDL encapsulating TCeNZ only), TCe‐C@rHDL, nHDL (Human‐derived natural HDL), CER‐001 mimetic, and CSL‐112 mimetic were determined using the SOD test kits and CAT test kits respectively.

#### 2.2.2 Resistance to Lipid and Protein Oxidation

rHDL samples (protein concentration = 0.3 mg/mL) were incubated with 50 µm CuSO_4_ and 150 µm H_2_O_2_, and the mixtures were shaken (120 rpm) at 37°C. Samples were collected at 0, 3, 6, 12, and 24 h, and malondialdehyde (MDA) and protein carbonyl content were measured using micro‐malondialdehyde kits and protein carbonyl content kits, respectively. In addition, according to the above protocol, the levels of MDA and protein carbonyl content in a co‐incubation system of nHDL and TCe‐C@rHDL (protein ratio = 1:1, w: w; total protein concentration 0.3 mg/mL) were measured to characterize the protective effect of TCe‐C@rHDL on nHDL.

#### 2.2.3 LPS Binding and Neutralization Capacity

**In vitro LPS binding assay**: All test formulations were mixed with FBS (final concentration 10%) and FITC‐LPS (final concentration 20 µg/mL). The mixtures were incubated in the dark at 37°C with constant shaking (120 rpm) for 30 min. The incubated mixtures were centrifuged to separate nanoparticles and unbound FITC‐LPS. The supernatant was transferred and the fluorescence intensity of residual (unbound) FITC‐LPS was measured using a multifunctional microplate reader (Infinite 200 PRO, TECAN, Switzerland). The PBS group was used as the negative control, and the LPS‐binding rate (%) was calculated according to the following formula:

$$\mathrm{LPS}\;\mathrm{binding}\;\left(\%\right)=\left(1-\frac{F_{test}}{F_{control}}\right)\times 100\%$$

**LPS neutralization assay under clinically relevant shear conditions**: Serum samples from Patient 1 and Patient 2 were mixed with varying concentrations (protein concentrations: 1, 0.5, and 0.25 mg/mL) of TCe‐C@rHDL or CER‐001 mimetic. The mixtures were circulated in a peristaltic pump system under simulated physiological blood shear stress (40 dyn/cm^2^) at 37°C for 30 min. After incubation, the remaining unbound LPS activity in each sample was quantified using a limulus amebocyte lysate (LAL) assay.

#### 2.2.4 Resistance to SAA‐Mediated apoA‐I Displacement

Fluorescence resonance energy transfer (FRET) occurred when energy transferred from an excited donor fluorophore (Coumarin 6, C6) to an acceptor fluorophore (Rhodamine B isothiocyanate, RBITC) within a proximity of <10 nm. C6 was incorporated into the lipid component; RBITC was conjugated to apoA‐I [40]. Three types of labeled formulations were prepared: C6 single‐labeled, RBITC single‐labeled, and C6/RBITC dual‐labeled.

**In vitro displacement assay**: Dual‐labeled formulations were mixed with SAA at a final concentration of 100 µg/mL (simulating levels during bacterial infection [41]) and incubated in the dark (37°C, 120 rpm). Samples were collected at 0, 5, 15, 30, and 60 min. Emission spectra were scanned using a multifunctional microplate reader (excitation: 465 nm). Additionally, RBITC single‐labeled formulation was mixed with SAA (100 µg/mL) and incubated in the dark at 37°C for 0 and 60 min. To remove unbound RBITC‐apoA‐I, the mixture was centrifuged using a 100 kDa molecular weight cutoff ultrafiltration device (Amicon Ultra, Merck, Germany). The fluorescence intensity of the retained supernatant was measured. The percentage of remaining apoA‐I bound to nanoparticles at 60 min was calculated relative to the 0‐min time point.

**Displacement assay under clinically relevant shear conditions**: Dual‐labeled formulations were mixed with serum from Patient 1 or Patient 2. The mixture was circulated in a peristaltic pump system under simulated physiological shear stress (40 dyn/cm^2^) at 37°C for 60 min. After incubation, the emission spectra of the dual‐labeled formulations were scanned using a fluorescence spectrophotometer (excitation: 466 nm). An additional negative control was performed by incubating the dual‐labeled formulation with PBS, following the same procedure. The FRET index of each sample was calculated according to the following formula:

$$\mathrm{FRET}\;\mathrm{index}=\frac{F_{b}}{D_{b}}-\frac{F_{d}}{D_{d}}$$

here, *F_b_* and *D_b_* represent the fluorescence intensities of the dual‐labeled formulation in channels *F* and *D*, while *F_d_* and *D_d_* represent the fluorescence intensities of the C6 single‐labeled formulation in channels *F* and *D* (Table S4).

#### 2.2.5 Pharmacokinetic and Biodistribution Studies in Physiological and Inflammatory Environments

**Pharmacokinetic profiling in rats**: SD rats were randomly divided into two groups: one group received no pretreatment and served as the physiological condition (LPS (−)) group, while the other group received an intravenous (i.v.) injection of 5 mg/kg LPS to induce systemic inflammation and SAA production [42]. Twelve hours post‐LPS injection, the latter group (LPS (+)) and the LPS (−) group received an i.v. injection of free DIR, DIR‐labeled TCe‐C@rHDL, or DIR‐labeled CER‐001 mimetic (DIR dose: 1 mg/kg). Blood samples were collected and the fluorescence signal was immediately captured using an in vivo imaging system (IVIS). Fluorescence intensity in the blood was quantified for pharmacokinetic analysis. Key pharmacokinetic parameters were subsequently calculated using the non‐compartmental model in PKsolver software.

**Biodistribution assessment in mice**: ICR mice were similarly randomized into two groups: a LPS (−) group and a LPS (+) group induced by an i.v. injection of 5 mg/kg LPS. Twelve hours later, both groups received an i.v. injection of free DIR, DIR‐labeled TCe‐C@rHDL, or DIR‐labeled CER‐001 mimetic (DIR dose: 1 mg/kg). Twenty‐four hours post‐formulation administration, the mice were euthanized. Major organs were harvested for ex vivo imaging using the IVIS system. The fluorescence intensity in each organ was quantified to assess and compare tissue distribution patterns under physiological versus inflammatory conditions.

### 2.3 Research on the Hierarchical Delivery Mechanism of TCe‐C@rHDL

#### 2.3.1 Evaluation of Mitochondrial Targeting Efficiency

FITC‐labeled oleylamine (FITC‐OA) was conjugated to the surface of TCe–C via hydrophobic interactions [43]. Specifically, FITC‐OA powder was added to TCe‐C dispersion in chloroform at a 1:1 molar ratio (OA‐FITC to Ce). The resulting mixture was stirred in the dark for 8 h to obtain the FITC‐labeled TCe–C complex. FITC‐labeled Ce–C complex were prepared using the identical procedure.

RAW 264.7 cells in the logarithmic growth phase were seeded onto confocal dishes at a density of 2 × 10^5^ cells/dish and incubated for 24 h. After removing the medium, cells were pretreated with 800 µm H_2_O_2_ for 2 h. Subsequently, FITC‐labeled Ce‐C@rHDL or TCe‐C@rHDL were added at a final Ce concentration of 60 µm. For the SR‐B1 inhibition control group, cells were pre‐incubated with 100 µm SR‐B1 inhibitor BLT‐1 concurrently with H_2_O_2_ pretreatment, followed by the addition of TCe‐C@rHDL. Following 1 h incubation, cells were washed and fixed. Then, cells were co‐stained with DAPI (nuclear) and Mito‐Tracker Deep Red FM (mitochondrial probe). Intracellular fluorescence distribution was visualized using a laser scanning confocal microscope (CLSM, STELLARIS 5, Leica, Germany). Pearson's correlation coefficient was quantified using ImageJ software to analyze fluorescence signal colocalization.

#### 2.3.2 Investigation of Macrophage Uptake Mechanisms

RAW 264.7 cells in the logarithmic growth phase were seeded in 24‐well plates at a density of 1 × 10^5^ cells/well and cultured for 24 h. Following medium removal, cells were pretreated for 2 h with 800 µm H_2_O_2_. Subsequently, FITC‐labeled Ce‐C@rHDL, TCe–C NPs, or TCe‐C@rHDL were added to respective wells at a final Ce concentration of 60 µm. After incubation periods (0.5, 1, 2, or 4 h), cells were washed and resuspended in PBS. Cellular fluorescence intensity was quantified using a flow cytometer (FACSCelesta, BD, USA). Quantitative analysis of flow cytometry data was performed using FlowJo software.

The involvement of specific endocytic pathways was evaluated in parallel experiments. During H_2_O_2_ pretreatment, cells were co‐incubated with the following pathway inhibitors for 2 h: 28 µm chlorpromazine (CPZ; clathrin‐mediated endocytosis inhibitor); 5 mm methyl‐β‐cyclodextrin (mβCD; caveolae‐mediated endocytosis inhibitor); 100 µm 5‐ (*N*‐ethyl‐*N*‐isopropyl)‐Amiloride (EIPA; macropinocytosis inhibitor); 100 µm BLT‐1 (SR‐B1 receptor inhibitor); 85 µm Z‐Phe‐Phe‐Phe‐OH (membrane fusion inhibitor); temperature blockade (4°C incubation to inhibit energy‐dependent endocytosis). Following inhibitor pretreatment, cells were processed identically for flow cytometric analysis as described above.

#### 2.3.3 Assessment of Membrane Fusion Mechanisms

RAW 264.7 cells were seeded onto confocal dishes and incubated for 24 h. After removing the medium, cells were pretreated with 800 µm H_2_O_2_ for 2 h. Subsequently, DiO‐labeled Ce‐C@rHDL, TCe–C NPs, or TCe‐C@rHDL were added to respective wells at a final Ce concentration of 60 µm. To investigate membrane fusion mechanisms, two inhibitor control groups (BLT‐1 and Z‐Phe‐Phe‐Phe‐OH) were established. Following 30 min incubation of formulations, cells were stained with plasma membrane dye DiI. After washing, cells were fixed and counterstained with DAPI. Following washes, membrane fusion dynamics were visualized using CLSM. Pearson's correlation coefficient was quantified using ImageJ software to analyze fluorescence signal colocalization.

### 2.4 Intracellular Fate and Degradation Kinetics of LPS‐Nanoparticle Complexes

#### 2.4.1 In Vitro LPS Clearance and Degradation Kinetics

The capacity of macrophages to clear and degrade nanoparticle‐bound LPS was quantitatively assessed using a LAL assay. The experimental design included the following groups: Control (untreated cells), LPS alone, LPS + chloroquine, TCe‐C@rHDL + LPS, TCe‐C@rHDL + LPS + chloroquine, TCe‐C@rHDL + LPS + BLT‐1, and CER‐001 mimetic + LPS. Nanoparticle formulations (at a protein concentration of 50 µg/mL) were pre‐incubated with LPS (1 µg/mL) in DMEM supplemented with 10% FBS at 37°C for 30 min to allow complex formation. RAW 264.7 cells, seeded in 24‐well plates at a density of 2 × 10^5^ cells/well and cultured for 24 h, were then incubated with the respective complexes for either 4 or 24 h. At each time point, cell culture supernatants were collected and centrifuged to remove cellular debris. Cells were washed with PBS and lysed on ice. To ensure complete release of nanoparticle‐bound LPS for accurate quantification, all samples were treated with guanidine hydrochloride to dissociate the rHDL structure. LPS activities were subsequently measured using a LAL kit according to the manufacturer's protocol.

#### 2.4.2 CLSM‐Based Spatiotemporal Tracking of Nanoparticle‐LPS Complexes

The intracellular trafficking and fate of nanoparticle‐LPS complexes in macrophages were dynamically monitored using CLSM. Nile Red‑labeled TCe‑C@rHDL or CER‑001 mimetic was incubated with FITC‑conjugated LPS in DMEM containing 10% FBS at 37°C for 30 min. RAW 264.7 cells were then incubated with these complexes for 30 min to capture the temporal progression of cellular uptake. Control conditions included free FITC‑LPS, as well as cells pretreated with either the SR‑B1 inhibitor BLT‑1 or the lysosomal acidification inhibitor chloroquine. After incubation, the medium was aspirated and cells were washed with PBS. Lysosomes were labeled by incubation with LysoTracker Red, followed by fixation, permeabilization, and blocking with 5% BSA. Immunofluorescence staining was performed using an Alexa Fluor 680‑conjugated anti‑TLR4 antibody. Nuclei were counterstained with DAPI. Following final PBS washes, five‑channel imaging was carried out on a CLSM system.

### 2.5 Evaluation of the Multimodal Immunomodulatory Effect of TCe‐C@rHDL

To simulate intervention strategies during the onset of sepsis, except where otherwise specified, all subsequent cell experiments employed the TPS intervention protocol: specifically, cells were first pre‐treated with H_2_O_2_ (800 µm) or LPS (1 µg/mL) for 2 h, followed by co‐incubation with the test formulations (Cel, 0.125 µm; Ce, 1.88 µm) for 24 h.

#### 2.5.1 Detection of Intracellular ROS and Oxidative Stress Levels

Single‐cell suspensions from (a) H_2_O_2_‐stimulated RAW 264.7 cells, or (b) peritoneal lavage fluid in the CLP model, were incubated with 10 µm DCFH‐DA under light‐protected conditions (37°C, 30 min). After washing and resuspension with PBS, intracellular ROS levels were quantified by BD flow cytometry. SOD activity and MDA levels served as oxidative stress biomarkers. Samples from (a) H_2_O_2_‐stimulated RAW 264.7 cells, or (b) livers in the systemic inflammation model, were homogenized and centrifuged to collect supernatant for analysis. Total protein concentration was determined using a BCA assay kit. SOD activities and MDA levels were measured using SOD assay kits and microscopic MDA assay kits, respectively.

#### 2.5.2 Detection of Apoptosis Levels

The apoptosis levels in (a) H_2_O_2_‐stimulated RAW264.7 cells and (b) cells from the peritoneal lavage fluid were detected using the Annexin V‐FITC/PI apoptosis detection kit and the Annexin V‐YSFluorTM 647/7AAD apoptosis detection kit, respectively. Fluorescence signals in each sample were detected using a BD flow cytometer. The levels of caspase‐3 activity and mitochondrial membrane potential (MMP) in RAW264.7 cells were detected using a live cell Caspase‐3 activity and mitochondrial membrane potential detection kit. Fluorescence signals in each sample were detected using a BD flow cytometer.

#### 2.5.3 Detection of Macrophage Polarization Phenotypes

**For RAW264.7 cells**: Following LPS pre‐stimulation and formulation treatment, cells were resuspended in 3% BSA solution to block at 37°C for 15 min. After washing, cells were incubated with APC‐CD86 antibody at 4°C for 60 min. Subsequently, cells were fixed and permeabilized. Finally, cells were resuspended and incubated with PE‐CD206 antibody at 4°C for 60 min. After washing and resuspension with PBS, fluorescence signals in each sample were detected using a BD flow cytometer.

**For peritoneal lavage fluid**: After processing into a single‐cell suspension, Fc receptors of cells were blocked by CD16/CD32 antibody at room temperature for 15 min. Then, cells were incubated with FITC‐CD11b antibody, PerCP/Cy5.5‐F4/80 antibody, and APC‐CD86 antibody at room temperature for 45 min. After fixation and permeabilization, cells were incubated with PE‐CD206 antibody at room temperature for 45 min. Finally, cells were washed and resuspended with PBS, and analyzed by flow cytometry.

#### 2.5.4 Detection of Inflammation‐Related Factor Levels

Following LPS pre‐stimulation and formulation treatment, the cell culture supernatant was collected and centrifuged to remove suspended cells. Levels of TNF‐α, IL‐10, HMGB1, and LPS in the supernatant were measured using respective ELISA kits. Besides, the levels of TNF‐α, IL‐10, IL‐1β, IL‐18, HMGB1, and LPS in the serum and peritoneal fluid samples were also determined using respective ELISA kits.

### 2.6 Transcriptomic Analysis

RAW264.7 cells were subjected to LPS stimulation and treated with TCe‐C@rHDL according to the experimental protocol described above. Total RNA was extracted from the LPS and TCe‐C@rHDL treatment groups. RNA quality and integrity were assessed and qualified RNA samples were used for library construction. Differentially expressed genes were identified with thresholds of [adjusted *P* < 0.05] and [|log2 fold change| ≥ 1]. KEGG pathway annotation and enrichment analyses were performed using the Majorbio Cloud Platform (www.majorbio.com). Heatmaps and volcano plots were generated based on the resulting differential‐expression dataset.

### 2.7 Systemic Inflammation Model

ICR mice were randomly divided into eight experimental groups: PBS, Cel, TCe NPs, TCe‐C NPs, TCe‐C@rHDL, rHDL, CER‐001 mimetic, and Control (healthy untreated mice). Mice in all groups except Control received an intravenous injection of LPS (15 mg/kg) to induce systemic inflammation. At 0.5 h post‐LPS challenge, the respective formulations were administered intravenously. Cel and Ce were administered at doses of 0.12 and 0.75 mg/kg, respectively, while the CER‐001 mimetic was administered at a dose matched to TCe‐C@rHDL on the basis of total lipid concentration. Twenty‐four hours after LPS administration, blood samples were collected from all mice. Subsequently, mice were euthanized and major organs (heart, liver, spleen, lungs, and kidneys) were harvested.

### 2.8 Pharmacodynamic Evaluation in the Systemic Inflammation Model

A batch of mice underwent identical LPS induction and treatment protocols for monitoring of survival rates and daily body weight changes over a 7‐day observation period (*n* = 10).

Fixed tissues were embedded in paraffin, sectioned, and subjected to H&E staining, terminal deoxynucleotidyl transferase dUTP nick end labeling (TUNEL) assay. Fresh samples of liver, lung, and kidney were snap‐frozen in liquid nitrogen for dihydroethidium (DHE) staining. Blood samples were centrifuged to obtain plasma for inflammatory cytokine quantification and biochemical analysis.

For immunofluorescence staining, liver sections were deparaffinized, rehydrated, and subjected to heat‐mediated antigen retrieval in EDTA buffer. After blocking with 3% BSA, sections were incubated overnight at 4°C with primary antibodies against NF‐κB, NLRP3, caspase‐1, caspase‐3, caspase‐11, GSDMD‐NT, iNOS, F4/80, and CD206, respectively. Following PBS washes, sections were incubated with Alexa Fluor‐conjugated secondary antibodies for 30 min at room temperature. Nuclei were counterstained with DAPI, and slides were mounted with antifade medium. Images were captured using a confocal microscope. Fluorescence intensity of each section was quantified with ImageJ software.

### 2.9 Cecal Ligation and Puncture (CLP) Surgery

The CLP model was established according to established protocols [44]. Prior to surgery, animals were fasted for 12 h and anesthetized via intraperitoneal injection of pentobarbital sodium. After shaving and disinfecting the abdominal area, a 1 cm midline incision was made to exteriorize the cecum. Approximately 75% of the cecal length was ligated using 4‐0 silk suture, followed by through‐and‐through puncture with a 22‐gauge needle. Fecal content was squeezed out from the puncture site. The cecum was then returned to the abdominal cavity, and the incision was closed in two layers. Postoperatively, all animals received subcutaneous saline resuscitation and were maintained on heating pads to prevent hypothermic shock.

### 2.10 Pharmacodynamic Evaluation in the CLP Model

ICR mice were randomly allocated to six experimental groups: Control (healthy untreated), Sham (laparotomy without CLP surgery), CLP, CIP (CLP surgery + 20 mg/kg ciprofloxacin hydrochloride i.p. at 0.5 h post‐CLP), TCe‐C@rHDL (CLP surgery + TCe‐C@rHDL containing 0.12 mg/kg Cel and 0.75 mg/kg Ce i.p. at 1 h post‐CLP), and Combine (CLP surgery + CIP at 0.5 h followed by TCe‐C@rHDL at 1 h with identical dosing).

At 24 h post‐treatment, blood samples were collected for inflammatory cytokine quantification, bacteriological culture, and biochemical analysis. Mice were subsequently euthanized, and peritoneal lavage was performed using 5 mL DMEM complete medium for flow cytometric analysis, inflammatory cytokine quantification, and bacteriological culture. Major organs (heart, liver, spleen, lungs, kidneys) were harvested for histopathological examination via H&E staining.

A separate batch of mice underwent identical CLP surgery and treatment protocols for monitoring of survival rates, daily body weight, and clinical score changes over a 7‐day observation period (*n* = 10). Clinical scores were assessed using an established scoring system [45] with the following predefined criteria: 0, no symptoms; 1, piloerection and huddling; 2, piloerection, diarrhea, and huddling; 3, lack of interest in surroundings and severe diarrhea; 4, decreased movement and listless appearance; 5, loss of self‐righting reflex. Animals reaching a score of 5 were humanely euthanized as a predetermined endpoint.

### 2.11 Statistical Analysis

Each experiment was independently repeated at least three times. The data were analyzed and presented as mean ± SD. Statistical comparisons between experimental groups were conducted using a one‐way analysis of variance (ANOVA) test, followed by Tukey's post hoc comparison test. The survival benefit was determined using a log‐rank test. */# indicates *P* < 0.05, **/## indicates *P* < 0.01, ***/### indicates *P* < 0.001, ****/#### indicates *P* < 0.0001, while ns indicates no significant difference between the two groups. Hash symbols (#) indicate significance relative to the TCe‐C@rHDL group. All statistical analyses were carried out using GraphPad Prism v.10.1.

## 3 Results and Discussion

### 3.1 Pathology‐Informed Profiling Identifies Representative Dysfunction Liabilities

**FIGURE 1:**
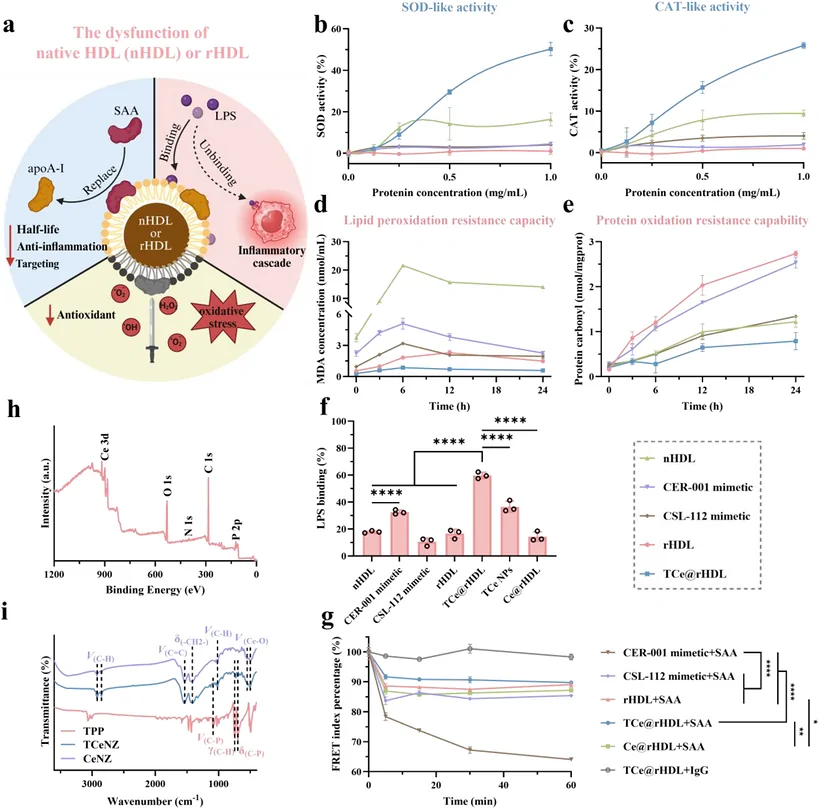
Pathology‐informed profiling of representative dysfunction liabilities and nanozyme‐armored functional protection. (a) Schematic illustration of the selected pathology‐relevant dysfunction liabilities of nHDL/rHDL under inflammatory stress, including oxidative damage to lipid and protein components, impaired LPS handling, and SAA‐mediated displacement of apoA‐I. (b,c) SOD‐like and CAT‐like activities of nHDL, rHDL, clinical‐stage rHDL mimetics, and nanozyme‐armored formulations. (d,e) Time‐dependent lipid oxidation and protein oxidation, measured as MDA and protein carbonyl content, respectively, after incubation with 50 µm Cu^2+^ and 150 µm H_2_O_2_ for 24 h. (f) LPS‐binding capacities of the indicated formulations in the presence of FITC‐LPS (20 µg/mL). (g) FRET‐based analysis of resistance to SAA‐mediated apoA‐I displacement after incubation with SAA (100 µg/mL) for 1 h. (h) XPS survey spectrum of TCeNZ. (i) FTIR spectrum of TCeNZ. Red labels indicate characteristic peaks of TPP; blue–purple labels indicate characteristic peaks of CeNZ. Data are presented as mean ± SD (*n* = 3).

HDL dysfunction during severe inflammation is multidimensional, involving changes in lipid and protein oxidation, endotoxin handling, cholesterol transport, receptor interactions, and acute‐phase compositional remodeling. Rather than attempting to comprehensively reproduce this entire spectrum, we focused on three representative sepsis‐relevant dysfunction liabilities that are directly linked to the intended immunomodulatory and delivery functions of rHDL [27, 28, 29, 30]: oxidative damage to lipid and protein components, impaired LPS handling, and SAA‐associated displacement of apoA‐I (Figure 1a). These parameters respectively reflect structural vulnerability under inflammatory oxidative stress, loss of an important endotoxin‐neutralizing function, and acute‐phase remodeling of the apoA‐I‐containing scaffold. Therefore, we benchmarked nHDL, conventional rHDL, and the CER‐001 and CSL‐112 mimetics against these representative liabilities under simulated inflammatory conditions as the initial pathology‐informed profiling step.

Superoxide dismutase (SOD) and catalase (CAT) are essential endogenous defenses against oxidative stress. As shown in Figure 1b,c, nHDL exhibited weak but detectable SOD‐ and CAT‐like activities, whereas all rHDL formulations showed little to no such activity, likely due to the loss of endogenous protective factors such as paraoxonase‐1. To further assess oxidative vulnerability, the formulations were exposed to a Cu^2+^/H_2_O_2_ system that generates abundant hydroxyl radicals and drives lipid and protein oxidation. Malondialdehyde (MDA) and protein carbonyl content were used as indicators of lipid and protein oxidation, respectively. Over 24 h, all formulations underwent progressive oxidative damage (Figure 1d,e). Notably, nHDL showed pronounced lipid oxidation, likely because its high content of unsaturated lipids makes it susceptible to free‐radical attack despite retaining limited antioxidant activity. Protein oxidation was also observed across all groups, indicating that both endogenous and reconstituted HDL are structurally vulnerable under inflammatory oxidative stress. Given that phospholipids and apolipoproteins are essential to HDL architecture and function, such oxidative damage is expected to directly impair the biological performance of rHDL.

We next examined LPS‐binding capacity, a key functional basis for the anti‐inflammatory activity of HDL‐based therapeutics (Figure 1f). Within 30 min, nHDL bound approximately 17.9% of LPS, consistent with previous reports [46]. In contrast, both the CSL‐112 mimetic and rHDL displayed weaker LPS‐binding capacity than nHDL, which may be attributed to the absence of endogenous LPS‐binding factors, such as lipopolysaccharide‐binding protein. Although the CER‐001 mimetic showed improved LPS binding, owing to its optimized phospholipid composition, the enhancement remained limited. These results indicate that inflammatory dysfunction in HDL‐based systems is not merely structural, but also functional, because reduced LPS capture would be expected to weaken their LPS‐clearing and immunomodulatory potential.

We finally assessed SAA‐induced apoA‐I displacement, another hallmark event in HDL dysfunction, using a fluorescence resonance energy transfer (FRET) assay [47]. To investigate the interaction between SAA and apoA‐I in the recombinant formulations, nHDL was excluded from this part. In this system, C6 and RBITC were used as a donor–acceptor pair to label phospholipids and apoA‐I, respectively [40]. In principle, SAA‐mediated displacement of apoA‐I increases the distance between the donor and acceptor, resulting in reduced FRET efficiency. As shown in Figure 1g and Figure S1, CER‐001 mimetic, CSL‐112 mimetic, and rHDL all exhibited clear FRET signals at baseline, indicating close association between apoA‐I and the lipid phase. After SAA challenge, FRET efficiency rapidly declined in all three formulations, with the extent of reduction following the order CER‐001 mimetic > CSL‐112 mimetic > rHDL. Independent ultrafiltration‐based quantification further showed a formulation‐dependent loss of particle‐associated apoA‐I after SAA exposure, consistent with the FRET trend (Figure S2). Because apoA‐I is central to both HDL bioactivity and receptor‐mediated trafficking, such displacement is expected to compromise not only immunomodulatory efficacy but also targeting precision and in vivo stability.

Collectively, these results identify oxidative deterioration, impaired LPS capture, and SAA‐driven apolipoprotein remodeling as the three representative liabilities of HDL‐based systems under inflammatory stress. Importantly, this pathology‐relevant dysfunction profile provided the basis for the subsequent design of the AEGIS strategy and for the progressive evaluation of its anti‐dysfunction performance.

### 3.2 Nanozyme Armoring Counteracts the Dysfunction Liabilities of rHDL

We next investigated whether these liabilities could be counteracted through AEGIS. To this end, TCeNZ was introduced as a nanozyme armor to preserve HDL‐like functions. TPP was selected as the mitochondrial‐targeting motif because its lipophilic cationic character enables membrane‐potential‐driven mitochondrial accumulation and has been extensively exploited for mitochondrial delivery of antioxidant and redox‐active cargos [48]. On this basis, TPP was grafted onto ceria nanozyme to produce TCeNZ. The resulting dispersion displayed a translucent yellow appearance with a distinct Tyndall effect (Figure S3), indicating the formation of a stable colloidal nanosystem. Dynamic light scattering (DLS) showed an average hydrodynamic diameter of 12.32 ± 0.30 nm with a low polydispersity index of 0.119 ± 0.010, supporting its uniform nanoscale structure. Successful TPP modification was further confirmed by XPS, in which a characteristic P 2p signal was detected (Figure 1h), and by FTIR, which revealed the characteristic C–P and C–H vibrations of TPP on the nanozyme surface (Figure 1i). XRD analysis demonstrated that TCeNZ retained the typical fluorite cubic structure of CeO_2_ (JCPDS No. 01‐080‐4829) (Figure S4). In addition, quantitative analysis showed that TCeNZ contained 35.63% Ce(III) (Figure S5), a redox state closely associated with the SOD‐ and CAT‐mimetic activities of ceria‐based nanozymes [49, 50]. Consistently, reversible color changes between yellow and colorless were observed during sequential H_2_O_2_ titration (Figure S6), reflecting dynamic Ce(IV)/Ce(III) redox cycling and confirming the sustained antioxidant nature of the nanozyme.

We then incorporated TCeNZ into rHDL to construct TCe@rHDL. This design substantially reinforced the anti‐dysfunction properties of rHDL. Compared with other rHDL formulations, TCe@rHDL showed markedly enhanced SOD‐ and CAT‐like activities (Figure 1b,c), which translated into significantly reduced lipid and protein oxidation over 24 h (Figure 1d,e). These data indicate that nanozyme compensates for the lack of endogenous antioxidant components in rHDL and shields the carrier from ROS‐driven structural damage. Importantly, TCe@rHDL also exhibited the highest LPS‐binding capacity among the tested rHDL formulations (Figure 1f). By contrast, control groups lacking apoA‐I incubation or TPP modification, such as TCe NPs or Ce@rHDL, displayed much weaker binding, indicating that both the HDL framework and TPP modification are required for optimal LPS capture. This enhancement may partly arise from TPP‐associated changes in interfacial charge, which could favor interaction with negatively charged LPS [51]. In support of this interpretation, although all formulations had comparable sizes in the range of 20–30 nm, positively charged formulations containing TCeNZ, such as TCe@rHDL and TCe NPs, bound LPS more efficiently than negatively charged or near‐neutral counterparts (Table S1), suggesting a contribution from interfacial electrostatics. However, despite comparable positive Zeta potentials, TCe@rHDL bound substantially more LPS than TCe NPs, indicating that surface charge alone is insufficient to explain the effect. Because the assay was performed in 10% FBS, serum‐mediated LPS transfer may also contribute: TPP‐associated electrostatics may facilitate initial LPS association, whereas the apoA‐I/phospholipid scaffold provides an additional lipoprotein‐like interface for LBP‐facilitated LPS sequestration. Notably, TCe@rHDL also showed the strongest resistance to the displacement of apoA‐I by SAA (Figure 1g; Figures S1,S2), retaining 85.5% ± 3.6% of apoA‐I after incubation, whereas Ce@rHDL exhibited significantly lower stability. Given that SAA binding to HDL is promoted by positively charged residues, while apoA‐I is negatively charged under physiological conditions [35, 36, 52], TPP‐associated changes in interfacial properties may contribute to the improved retention of particle‐associated apoA‐I after SAA challenge.

Collectively, these results show that nanozyme armoring substantially counteracts the three major dysfunction liabilities identified above. However, in the highly inflammatory milieu of sepsis, where persistent ROS generation and danger signals continuously amplify pathological stress, additional intervention is required to decouple disease‐driven microenvironmental damage from ongoing carrier dysfunction.

### 3.3 ATP‐Responsive TCe–C Coordination Supports Intracellular Cargo Separation and Hierarchical Delivery

**FIGURE 2:**
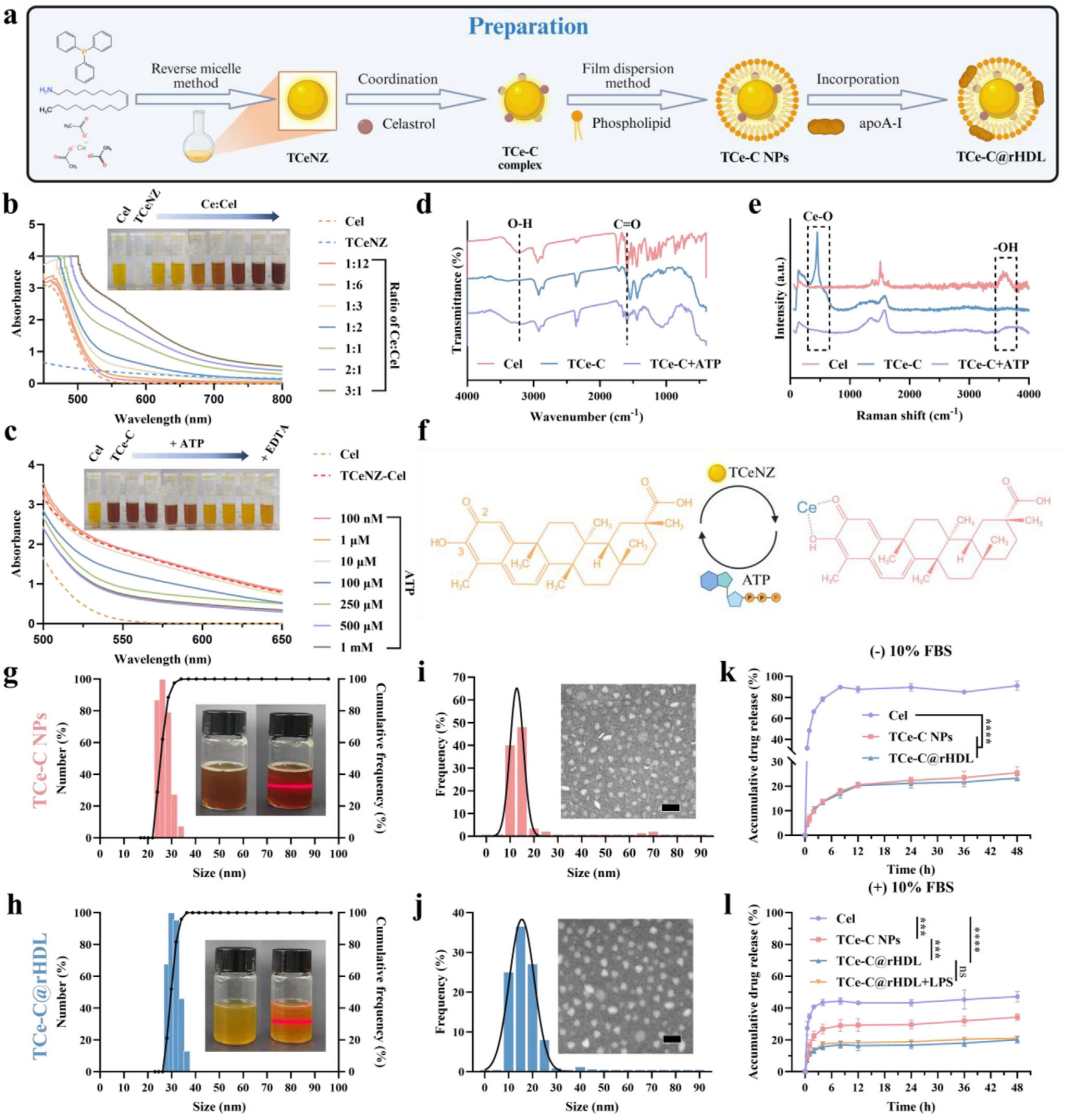
ATP‐responsive coordination of TCe–C and its structural integration into the final anti‐dysfunction rHDL nanoplatform. (a) Schematic illustration of the preparation of TCe–C, TCe–C NPs, and TCe‐C@rHDL. (b) UV–vis spectra of Cel solution (0.4 mm) after addition of increasing molar ratios of TCeNZ. (c) UV–vis spectra of TCe–C dispersion in the presence of increasing concentrations of ATP. (d,e) FTIR and Raman spectra of Cel, TCe–C, and TCe–C + ATP. (f) Schematic illustration of Cel‐TCeNZ coordination and ATP‐triggered dissociation of the TCe–C complex. (g,h) Particle size distribution, representative photographs, and Tyndall effect of TCe–C NPs and TCe‐C@rHDL. (i,j) TEM images and corresponding size distributions of TCe–C NPs and TCe‐C@rHDL. Scale bar, 50 nm. (k,l) Cumulative Cel release profiles of free Cel, TCe–C NPs, and TCe‐C@rHDL in pH 7.4 PBS with or without 10% FBS. Data are presented as mean ± SD (*n* = 3).

To couple carrier protection with inflammatory microenvironment intervention, we further introduced Cel, an HMGB1‐antagonistic and anti‐inflammatory agent, and coordinated it with TCeNZ to form a responsive functional unit, the TCe–C complex. Upon dropwise addition of TCeNZ into the Cel solution, the mixture gradually changed from yellow to dark brown (Figure 2b), suggesting the formation of a new coordination state. UV–vis spectroscopy further supported this process: a broad absorption band appeared at 500–650 nm, characteristic of ligand‐to‐metal charge transfer (LMCT) [53], and the absorbance at 550 nm increased with the Ce/Cel ratio. Saturation at a 1:1 molar ratio indicated that TCe–C was formed with a stoichiometric coordination ratio of 1:1 (Figure S7).

This coordination strategy was further employed for controlled release. Because ATP possesses strong affinity for metal ions through its triphosphate groups [54], we hypothesized that high‐level ATP could competitively dissociate Cel from TCe–C after intracellular delivery. This is particularly advantageous given that physiological ATP concentrations range from 10 to 100 nm in healthy tissue interstitium to hundreds of micromolar in stimulated or diseased tissues, with intracellular levels reaching up to millimolar concentrations [55, 56]. Consistent with this design, when ATP concentration exceeded 100 µm, the dark brown solution gradually reverted to orange–yellow and the LMCT absorption band disappeared (Figure 2c), indicating disruption of the coordination interaction. Similar results were obtained with EDTA, further confirming that the dissociation was driven by competitive metal binding. Spectroscopic analyses provided further evidence for this ATP‐responsive coordination behavior. In FTIR spectra, the characteristic peaks of Cel at 3213.8 cm^−1^ (O─H stretching) and 1649.8 cm^−1^ (C═O stretching) disappeared after complex formation but reappeared following ATP treatment (Figure 2d). Raman spectroscopy showed a similar trend: the characteristic ─OH signal of Cel vanished in TCe–C, while a Ce–O peak emerged at 452 cm^−1^; after ATP challenge, the Ce–O signal disappeared and the ─OH signal was restored (Figure 2e). These data indicate that Cel coordinates with cerium through its C2 carbonyl and C3 hydroxyl groups, and that high ATP levels can competitively break this interaction (Figure 2f). Together, these results establish TCe–C as a responsive integration of TCeNZ and Cel, enabling the anti‐dysfunction design to extend beyond carrier protection toward microenvironment intervention while preserving the potential for intracellularly triggered cargo release.

### 3.4 Structural Integration of TCe–C Into rHDL Yields a Stable Anti‐Dysfunction Nanoplatform (TCe‐C@rHDL)

We next integrated TCe‐C complex into rHDL framework through a thin‐film dispersion/self‐assembly process to obtain the final nanoplatform, TCe‐C@rHDL (Figure 2a). TCe–C nanoparticles without apoA‐I incorporation (TCe–C NPs) were prepared in parallel as a non‐rHDL control. Before final assembly, the formulation parameters of TCe–C NPs were optimized by screening multiple Ce/Cel feeding ratios (1:1, 3:1, 6:1, 9:1, and 15:1). Among them, the 9:1 ratio provided the most balanced profile in particle size, polydispersity, component content, and moderately positive surface charge (Table S2), and was therefore selected for subsequent construction of TCe‐C@rHDL.

The successful assembly of TCe‐C@rHDL was confirmed by its physicochemical properties. As shown in Figure 2g,h, both TCe–C NPs and TCe‐C@rHDL formed stable colloidal dispersions with evident Tyndall effects. DLS showed that the hydrodynamic diameter increased from 24.82 ± 0.79 nm for TCe–C NPs to 28.05 ± 3.20 nm for TCe‐C@rHDL, indicating successful apoA‐I incorporation. Transmission electron microscopy (TEM) further revealed near‐spherical nanoparticles with average diameters of 12.80 ± 2.82 nm and 15.54 ± 5.21 nm, respectively (Figure 2i,j), which were close to that of native HDL. The discrepancy in DLS and TEM size is possibly attributable to hydration layers [57]. In addition, the encapsulation efficiency and drug loading of TCeNZ and Cel were quantified (Table S3). Successful loading of TCe–C was further verified by the retained LMCT absorption features in the UV–vis spectra (Figure S8). Together, these results indicate that the final system retains the nanoscale structural characteristics necessary for HDL‐mimetic transport.

We next examined whether this assembly strategy provides the structural protection required for in vivo application. Both TCe–C NPs and TCe‐C@rHDL remained stable during storage at 4°C for 14 days. Under plasma‐mimicking conditions, TCe‐C@rHDL exhibited greater stability over 48 h than TCe–C NPs (Figure S9). This effect is attributable to the HDL shell, which provides a biomimetic environment that protects the responsive complex from premature disturbance. This protective effect was further evident in ATP‐triggered dissociation studies. Free TCe–C rapidly dissociated upon ATP exposure (Movie S1), whereas nanoencapsulated formulations especially TCe‐C@rHDL retained the coordination state for substantially longer periods (Figure S10). Thus, nanoencapsulation buffers ATP accessibility to the coordination core, enabling the system to remain stable during circulation. In vitro release studies supported this interpretation. Both formulations showed comparable profiles in pH 7.4 PBS, with approximately 24% cumulative release over 48 h (Figure 2k). However, TCe‐C@rHDL exhibited 1.7‐fold lower cumulative release than TCe–C NPs under plasma‐simulated conditions (Figure 2l). Additionally, LPS did not trigger Cel leakage from TCe‐C@rHDL, further confirming its stability under inflammatory conditions. Collectively, these data indicate that incorporation of TCe–C into the rHDL framework provides the stability needed to preserve its anti‐dysfunction design, supporting subsequent evaluation of TCe‐C@rHDL in clinically relevant inflammatory environments.

### 3.5 AEGIS Preserves rHDL Function Across Escalating Pathological Stress

**FIGURE 3:**
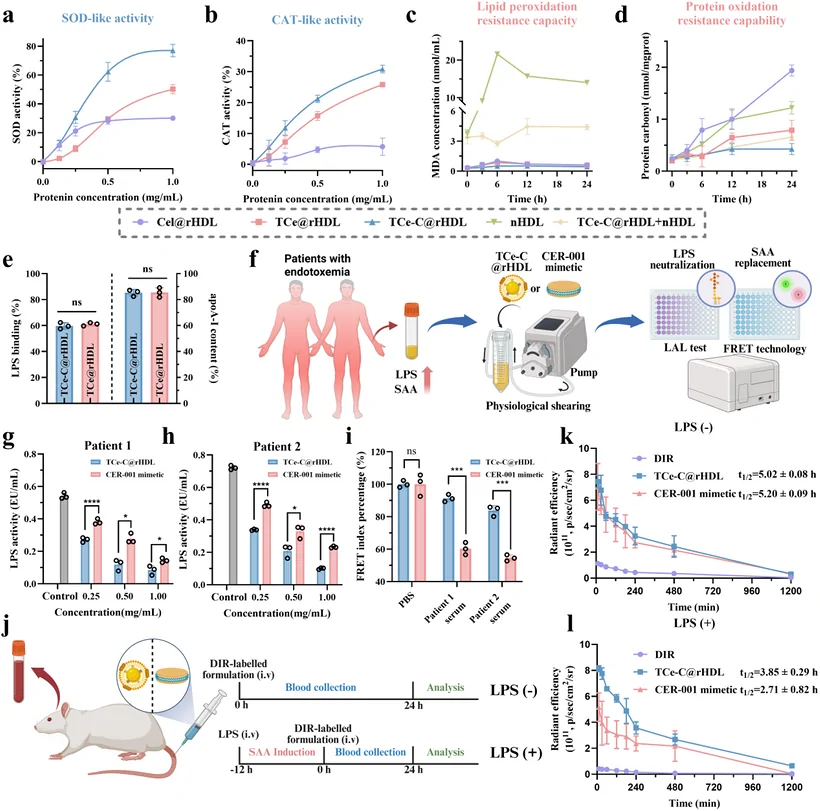
TCe‐C@rHDL preserves anti‐dysfunction performance across progressively more stringent inflammatory environments. (a,b) SOD‐like and CAT‐like activities of Cel@rHDL, TCe@rHDL, and TCe‐C@rHDL. (c,d) MDA and protein carbonyl levels after incubation with 50 µm Cu^2+^ and 150 µm H_2_O_2_ for 24 h in Cel@rHDL, TCe@rHDL, TCe‐C@rHDL, nHDL, and nHDL co‐incubated with TCe‐C@rHDL (TCe‐C@rHDL + nHDL). (e) Comparison of LPS‐binding capacity and residual apoA‐I content after SAA challenge in TCe@rHDL and TCe‐C@rHDL. (f) Schematic of the clinically relevant shear‐stress assay used to compare TCe‐C@rHDL and the CER‐001 mimetic in endotoxemia patient serum. (g,h) LPS‐neutralizing activities of TCe‐C@rHDL and the CER‐001 mimetic after incubation with serum from Patient 1 and Patient 2 under physiological shear conditions (40 dyn/cm^2^). (i) Percentage change in FRET index of TCe‐C@rHDL and the CER‐001 mimetic after incubation with patient serum under shear conditions. (j) Schematic of the pharmacokinetic study design under physiological [LPS (−)] and inflammatory [LPS (+)] conditions. (k,l) Plasma concentration‐time profiles of the indicated formulations under physiological and inflammatory conditions. Data are presented as mean ± SD (*n* = 3).

Building on the structural stability established above, we next asked whether TCe‐C@rHDL could preserve anti‐dysfunction performance across progressively more stringent inflammatory environments. Under simulated inflammatory conditions, incorporation of the TCe–C complex did not compromise the anti‐dysfunction properties conferred by nanozyme armoring. Instead, TCe‐C@rHDL further enhanced SOD‐ and CAT‐like activities relative to TCe@rHDL (Figure 3a,b), likely because Cel provided additional direct antioxidant effects against superoxide and hydrogen peroxide. Consistent with this, TCe‐C@rHDL showed lower levels of lipid and protein oxidation over 24 h (Figure 3c,d). Notably, when co‐incubated with nHDL, TCe‐C@rHDL also reduced oxidative damage to native HDL components, suggesting that its protective capacity extends beyond self‐preservation to the surrounding HDL microenvironment. At the same time, its LPS‐binding ability and resistance to SAA‐mediated apoA‐I displacement remained comparable to those of TCe@rHDL (Figure 3e).

We next performed an exploratory ex vivo assessment using serum from two patients with endotoxemia under physiological shear stress, to determine whether the anti‐dysfunction phenotype could be retained in complex pathological serum matrices. This assessment is critical, as biomimetic systems that function under simplified in vitro conditions often fail when exposed to pathological serum components, shear stress, and in vivo remodeling. Therefore, TCe‐C@rHDL was benchmarked against the CER‐001 mimetic under physiological shear stress using serum from two endotoxemia patients (Figure 3f). Under these conditions, both formulations showed concentration‐dependent LPS neutralization, but TCe‐C@rHDL consistently exhibited stronger neutralizing activity at every tested concentration in both patient samples (Figure 3g,h). Resistance to structural remodeling was also more pronounced in TCe‐C@rHDL. After circulation in patient serum under shear, TCe‐C@rHDL retained 91.4% and 83.7% of its initial FRET efficiency in samples from Patient 1 and Patient 2, respectively, whereas CER‐001 mimetic retained only 60.3% and 54.5% (Figure 3i; Figure S11).

Because inflammation‐driven remodeling could alter the in vivo fate of biomimetic carriers, we further compared the pharmacokinetic behavior of TCe‐C@rHDL and CER‐001 mimetic under physiological and inflammatory conditions. Under physiological conditions, both formulations showed prolonged and similar circulation, with half‐lives (*t*_1/2_) of 5.02 ± 0.08 h for TCe‐C@rHDL and 5.20 ± 0.09 h for CER‐001 mimetic (Figure 3k). However, after LPS pretreatment, the half‐life of CER‐001 decreased by 47.9% (to 2.71 ± 0.82 h), whereas that of TCe‐C@rHDL declined by only 23.3% (to 3.85 ± 0.29 h) (Figure 3l). This difference suggests that inflammatory dysfunction not only impairs molecular functions such as LPS capture and apoA‐I retention, but also compromises systemic behavior. Biodistribution studies further revealed distinct patterns. Under physiological conditions, both TCe‐C@rHDL and TCe–C NPs exhibited higher accumulation in the lungs and spleen compared to CER‐001 mimetic (Figure S12a), likely due to their positive surface charge and the metabolic pathways of ceria oxides [58, 59]. Under inflammatory conditions, all formulations showed altered tissue distribution. Quantification of fluorescence intensity changes indicated that CER‐001 mimetic accumulation decreased substantially in the liver and spleen, while TCe‐C@rHDL was cleared more slowly from these organs (Figure S12b–d). The accelerated clearance of rHDL formulations during sepsis is consistent with established pathophysiology, driven mainly by SAA‐mediated displacement of apoA‐I and oxidative damage to lipoprotein structure [16, 27, 60]. Taken together, these findings indicate that AEGIS preserves biomimetic function under clinically relevant inflammatory stress and thereby provides foundation for the subsequent assessment of therapeutic efficacy and biosafety.

### 3.6 TCe‐C@rHDL Confers Functional Advantages Without Compromising Biosafety

Given the inherent and dose‐limiting toxicity of Cel, a systematic safety evaluation of TCe‐C@rHDL was essential. However, in our design, stable coordination of Cel within the TCe–C complex, together with rHDL encapsulation, was expected to reduce premature exposure of free Cel during circulation and thereby improve biosafety while preserving therapeutic activity. First, hemolysis assays showed that all tested formulations except free Cel solution exhibited hemolysis rates below 5% across the investigated concentration range (Figure S13), indicating good blood compatibility after nanoformulation. Cell compatibility was then evaluated by MTT assay (Figure S14). Free Cel and TCe NPs maintained cell viabilities above 80% over relatively broad concentration ranges, whereas TCe‐C NPs and TCe‐C@rHDL showed a modest decline in viability, likely due to enhanced intracellular delivery and accumulation of Cel after nanoencapsulation. Nevertheless, TCe‐C@rHDL still maintained cell viability above 80% at Cel concentrations of 0.063–0.5 µm, indicating a reasonable in vitro safety window. We next examined in vivo safety across a range of payload doses. rHDL formulations containing different doses of Ce (0.75 and 1.5 mg/kg) and/or Cel (0.08, 0.12, and 0.18 mg/kg) caused no significant changes in organ coefficients compared with the Control group (Figure S15a). Serum biochemical indicators of liver and kidney function, including ALT, AST, BUN, and CREA, showed only a mild and statistically non‐significant upward trend with increasing Cel dose (Figure S15b). In addition, complete blood counts remained essentially unchanged (Figure S15c,d), and H&E staining showed no obvious histopathological abnormalities in major organs (Figure S16). Together, these results support a favorable safety profile for TCe‐C@rHDL despite incorporating Cel.

### 3.7 TCe‐C@rHDL Preserves HDL‐Intrinsic Routing to Enables Hierarchical Therapeutic Delivery and Lysosomal Disposal of Captured LPS

Following validation of the anti‐dysfunction performance, we next moved from evaluating preserved anti‐dysfunction performance to elucidating the delivery mechanism by which the AEGIS design translates this advantage into functional intracellular trafficking. By coupling the intrinsic SR‐B1‐mediated transport pathway of HDL with ATP‐responsive dissociation of the TCe–C complex and TPP‐assisted mitochondrial targeting, this design was expected to enable hierarchical intracellular delivery, featuring cytoplasmic release of Cel alongside mitochondrial accumulation of TCeNZ.

**FIGURE 4:**
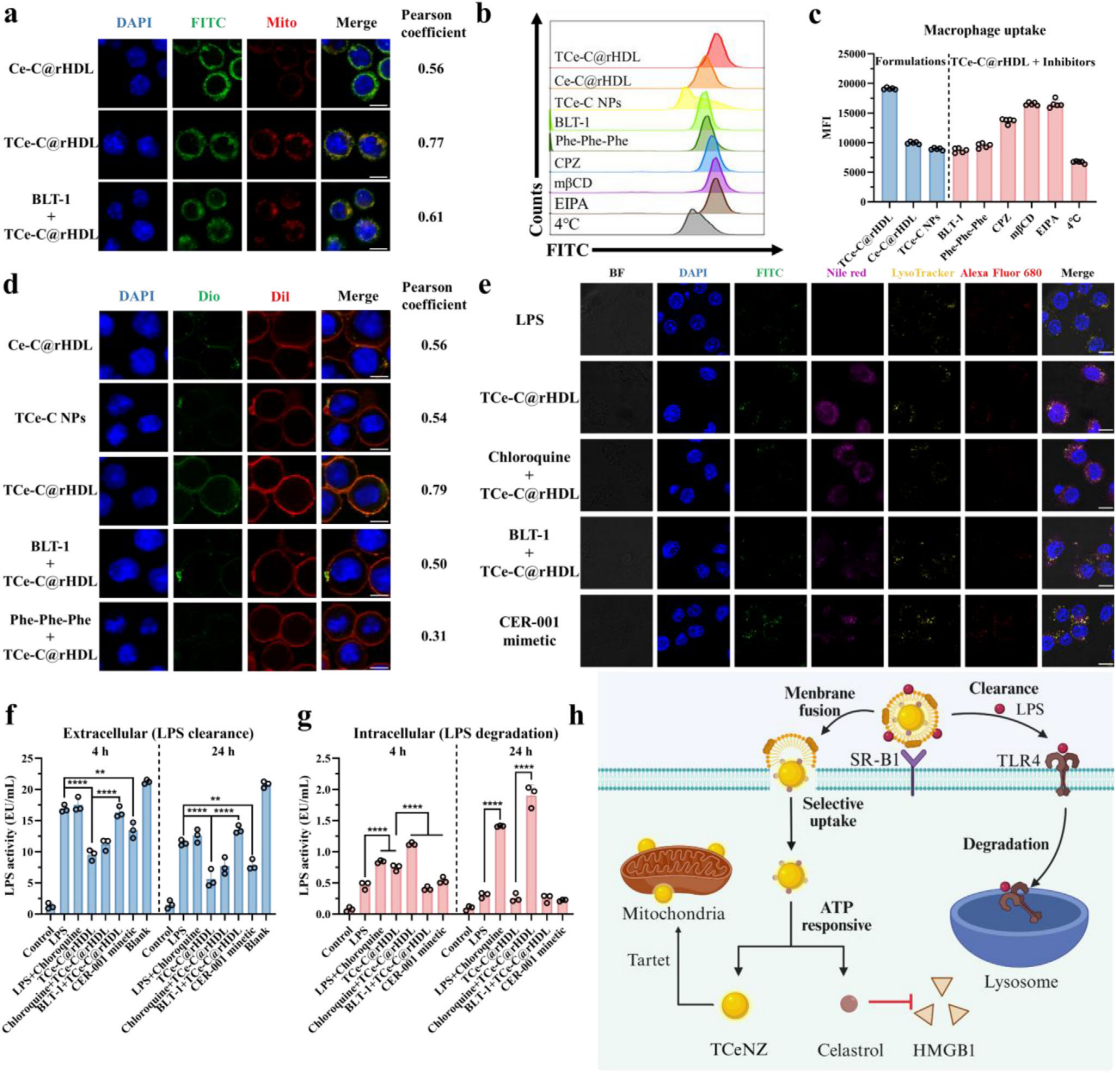
Hierarchical intracellular delivery of TCe‐C@rHDL and the intracellular fate of nanoparticle‐bound LPS. (a) Representative CLSM images showing mitochondrial targeting of TCeNZ/CeNZ in RAW264.7 cells. Blue, nucleus; green, FITC‐labeled TCeNZ/CeNZ; red, mitochondria. Scale bars, 8.5 µm. (b,c) Flow cytometry analysis and quantification of cellular uptake of the indicated TCe–C formulations in RAW264.7 cells (*n* = 5). (d) Representative CLSM images showing membrane fusion behavior of TCe‐C@rHDL in RAW264.7 cells. Blue, nucleus; green, DiO‐labeled formulations; red, plasma membrane. Scale bars, 6.7 µm. (e) Representative CLSM images tracking the intracellular fate of LPS‐nanoparticle complexes. Blue, nucleus; green, FITC‐LPS; magenta, Nile Red‐labeled Cel; yellow, lysosomes; red, Alexa Fluor 680‐labeled TLR4. Scale bars, 10.8 µm. (f,g) Kinetics of extracellular LPS clearance and intracellular LPS degradation at 4 and 24 h, quantified by LAL assay (*n* = 3). (h) Schematic summary of the hierarchical delivery mechanism of TCe‐C@rHDL and the intracellular processing of nanoparticle‐bound LPS. Data are presented as mean ± SD.

We first examined whether TPP modification improved mitochondrial targeting. CLSM showed that both Ce‐C@rHDL and TCe‐C@rHDL displayed appreciable mitochondrial localization, likely because their hydrophobicity conducive to mitochondrial targeting. However, Pearson's coefficient analysis revealed substantially stronger co‐localization of TCeNZ with mitochondria than CeNZ (0.77 vs. 0.56; Figure 4a), demonstrating that TPP further reinforces mitochondrial tropism after intracellular delivery. Notably, this co‐localization was partially reduced by BLT‐1 pretreatment, indicating that SR‐B1 contributes importantly to the intracellular routing of the hydrophobic core, although additional uptake processes may also participate.

We next quantified cellular uptake of the different formulations in macrophages. Within 4 h, uptake followed the order TCe‐C@rHDL > Ce‐C@rHDL ≈ TCe–C NPs (Figure 4b,c; Figure S17), indicating that both HDL‐based scaffold and TPP modification facilitate intracellular delivery of TCe–C. Mechanistic inhibition studies further showed that uptake of TCe‐C@rHDL was markedly suppressed by BLT‐1, the membrane fusion inhibitor Z‐Phe‐Phe‐Phe‐OH, and low temperature treatment, whereas only minor effects were observed after inhibition of clathrin‐mediated endocytosis, caveolae‐mediated endocytosis, or macropinocytosis. These results suggest that SR‐B1‐associated membrane lipid transfer contributes substantially to TCe‐C@rHDL internalization [21], whereas the tested classical endocytic pathways appear to play more limited roles. This is a key mechanistic advantage, because membrane fusion can bypass lysosomal sequestration and thereby better preserve intracellular bioavailability of the functional cargo.

To visualize this process more directly, membrane‐fusion behavior was further evaluated by CLSM using DiO‐labeled formulations and DiI‐labeled macrophage membranes. TCe‐C@rHDL exhibited a high membrane co‐localization coefficient of 0.79, significantly greater than that of Ce‐C@rHDL (0.56) or TCe–C NPs (0.54) (Figure 4d), confirming that both apoA‐I and the positively charged TPP modification promote close membrane interaction and fusion. Importantly, this co‐localization was reduced to 0.50 and 0.31 after BLT‐1 or Z‐Phe‐Phe‐Phe‐OH treatment, respectively, providing further evidence that SR‐B1‐associated membrane lipid transfer contributes importantly to the uptake behavior of TCe‐C@rHDL.

We next asked how the therapeutic components and captured LPS were processed after cellular entry. CLSM‐based intracellular fate analysis provided cellular‐level evidence complementary to the cell‐free ATP‐responsive dissociation studies (Figure 4e; Figure S18). Free LPS showed pronounced co‐localization with both TLR4 and lysosomes, consistent with receptor‐associated intracellular trafficking. After association with TCe‐C@rHDL, the intracellular LPS signal increased and remained substantially co‐localized with TLR4 and lysosomal compartments, indicating enhanced macrophage uptake and subsequent lysosomal processing of captured LPS. In contrast, the Nile Red‐labeled Cel‐associated signal exhibited a predominantly diffuse cytoplasmic distribution rather than punctate lysosomal confinement. Together with the preferential mitochondrial localization of TCeNZ observed in Figure 4a, these spatially distinct intracellular distributions indicate that the therapeutic components and captured LPS do not remain co‐localized after cellular uptake and are consistent with intracellular cargo separation followed by differential subcellular routing. Importantly, these CLSM observations provide intracellular evidence for component separation and hierarchical distribution, but do not directly identify the molecular trigger responsible for TCe–C dissociation. When considered together with the spectroscopic evidence showing that ATP can disrupt TCe–C coordination at intracellularly relevant concentrations, the data support the possibility that the ATP‐rich intracellular environment contributes to complex dissociation after cellular delivery. The subsequent inhibitor studies further clarified the cellular entry and processing pathways. Chloroquine treatment reduced LPS‐lysosome co‐localization, supporting the involvement of lysosomal processing in the intracellular fate of captured LPS, whereas BLT‐1 pretreatment markedly reduced both LPS and Nile Red‐associated intracellular signals, suggesting an important contribution of SR‐B1‐associated uptake to the delivery process. In comparison, the CER‐001‐like comparator also promoted intracellular LPS processing, whereas its Nile Red‐associated signal appeared predominantly punctate with greater lysosomal co‐localization, consistent with increased lysosomal sequestration of the associated cargo.

To quantify this process, extracellular clearance and intracellular degradation of nanoparticle‐bound LPS were measured by LAL assay (Figure 4f,g). TCe‐C@rHDL showed significantly greater extracellular LPS clearance than CER‐001 mimetic at both 4 and 24 h, consistent with its stronger LPS‐binding capacity observed in earlier sections. This effect was substantially attenuated by BLT‐1, indicating that efficient clearance depends on SR‐B1‐mediated uptake by macrophages. Intracellularly, the TCe‐C@rHDL group displayed higher recoverable LPS activity at 4 h, consistent with enhanced internalization, followed by a marked decline at 24 h. The persistence of intracellular LPS activity after chloroquine treatment, together with the CLSM localization data, supports the involvement of lysosomal processing in this decline. By contrast, chloroquine treatment resulted in sustained intracellular LPS activity, supporting an important contribution of lysosomal processing to the intracellular fate of captured LPS.

Collectively, these findings support a model in which TCe‐C@rHDL enhances LPS capture and SR‐B1‐associated cellular uptake, followed by TLR4/lysosome‐associated intracellular processing and a progressive reduction in recoverable LPS activity. Specifically, through apoA‐I/SR‐B1 recognition, the TCe‐C@rHDL‐LPS complex is efficiently captured by macrophages; the therapeutic components subsequently exhibited differential intracellular localization, with Cel‐associated signal distributed predominantly in the cytoplasm and TCeNZ showing preferential mitochondrial accumulation, whereas captured LPS underwent TLR4‐associated lysosomal processing (Figure 4h). In this way, TCe‐C@rHDL integrates endotoxin detoxification with functional cargo delivery in a single coordinated process, outperforming CER‐001 mimetic and providing a mechanistic bridge between preserved carrier functionality and downstream multimodal immunotherapy.

### 3.8 Hierarchical Delivery Amplifies the Multimodal Immunomodulatory Effects of TCe‐C@rHDL

The routing and cargo‐processing advantages established above should ultimately converge on a therapeutic endpoint, namely whether TCe‐C@rHDL can more effectively reprogram inflammatory macrophages at the functional level. We therefore next examined the cellular pharmacodynamic consequences of this design under oxidative and inflammatory stimulation, with particular focus on the interconnected processes of ROS accumulation, mitochondrial injury, apoptotic signaling, macrophage polarization, and upstream inflammatory trigger control [61, 62, 63].

We first assessed whether Cel and TCeNZ act synergistically in macrophages. MTT assays combined with combination index (CI) analysis showed that the Cel/TCeNZ combination produced robust synergistic effects across all tested models, with CI values ranging from 0.04 to 0.41 (Figure S19). Notably, this synergism was maintained under both oxidative and inflammatory stimulation, as well as in both pre‐intervention (TBS) and post‐intervention (TPS) regimens, indicating that the cooperation between Cel and TCeNZ is stable across distinct pathological settings. Among all tested formulations, TCe‐C@rHDL consistently showed the strongest protective effect, suggesting that hierarchical delivery further amplifies the intrinsic synergy between the two functional components.

**FIGURE 5:**
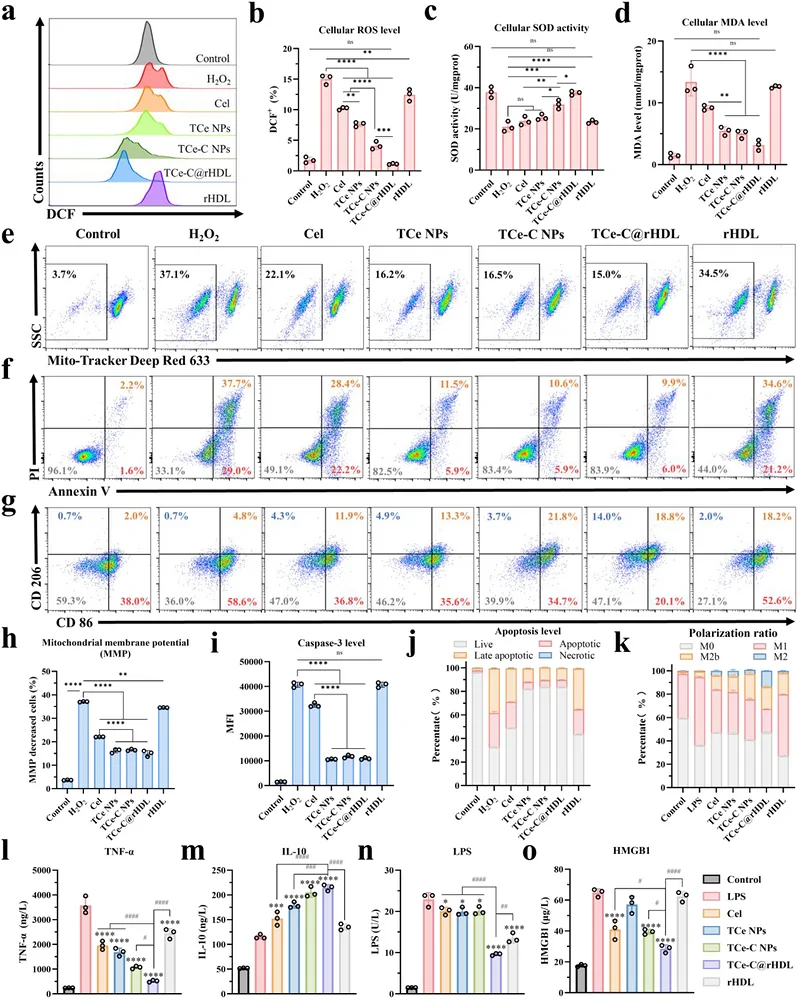
Hierarchical delivery amplifies the multimodal immunomodulatory effects of TCe‐C@rHDL in vitro. (a,b) Flow cytometry analysis and quantification of intracellular ROS levels in RAW264.7 cells stimulated with 800 µm H_2_O_2_. (c,d) Intracellular SOD activity and MDA level in H_2_O_2_‐stimulated RAW264.7 cells. (e) Flow cytometry analysis of mitochondrial membrane potential (MMP) in H_2_O_2_‐stimulated RAW264.7 cells. (f) Flow cytometry analysis of apoptosis in H_2_O_2_‐stimulated RAW264.7 cells. Quadrants: Annexin V‐/PI‐, live cells; Annexin V+/PI−, early apoptotic cells; Annexin V+/PI+, late apoptotic cells; Annexin V−/PI+, necrotic cells. (g) Flow cytometry analysis of macrophage polarization in RAW264.7 cells stimulated with LPS (1 µg/mL). Quadrants: CD86‐/CD206‐, M0; CD86+/CD206‐, M1; CD86+/CD206+, M2b; CD86‐/CD206+, M2. (h–k) Quantification of the MMP‐depolarized cell ratio, caspase‐3 activity, apoptotic cell ratio, and polarization phenotype ratios, respectively. (l–o) Levels of TNF‐α, IL‐10, LPS, and HMGB1 in the supernatant of LPS‐stimulated RAW264.7 cells. Data are presented as mean ± SD (*n* = 3).

Intracellular oxidative stress was first assessed, because it is a primary pathogenic driver of macrophage dysfunction [64]. Flow cytometric analysis of DCFH‐DA fluorescence showed that both Cel and TCe NPs reduced ROS accumulation in H_2_O_2_‐stimulated RAW264.7 cells, whereas their combination in TCe–C NPs further decreased the proportion of ROS‐high cells (Figure 5a,b). TCe‐C@rHDL produced the most pronounced effect, reducing intracellular ROS to a level not significantly different from that of untreated control cells. Consistently, measurements of SOD activity and MDA level showed the same trend (Figure 5c,d): TCe‐C NPs outperformed either single‐component formulation, confirming synergistic antioxidant activity, while TCe‐C@rHDL further strengthened this effect.

Because oxidative stress is closely coupled to mitochondrial dysfunction and apoptosis [65], we next evaluated mitochondrial membrane potential (MMP), caspase‐3 activation, and overall apoptotic cell death. H_2_O_2_ stimulation caused a marked increase in MMP depolarization, caspase‐3 activity, and apoptotic cell ratio, consistent with activation of the mitochondrial apoptotic pathway [66]. Although both Cel and TCe NPs partially alleviated these changes, TCe NPs showed a stronger effect than Cel alone, highlighting the central role of mitochondrial ROS control in preventing apoptosis. Importantly, TCe–C NPs further reduced MMP loss and caspase‐3 activation, while TCe‐C@rHDL achieved the strongest overall anti‐apoptotic effect (Figure 5e,f,h–j; Figure S20). These results indicate that combining Cel with TCeNZ not only suppresses oxidative stress, but also interrupts its downstream conversion into mitochondrial injury and cell death.

We then assessed macrophage polarization, a key determinant of immune homeostasis in sepsis. As shown in Figure 5g,k, LPS stimulation drove RAW264.7 cells toward a pro‐inflammatory M1 phenotype, whereas all therapeutic formulations induced varying degrees of reversal. Among them, TCe‐C@rHDL most effectively reduced the M1 population while promoting M2 polarization, demonstrating the strongest capacity to reprogram macrophages toward a pro‐resolving phenotype. This phenotypic shift was further supported by cytokine analysis: ELISA showed that TCe‐C@rHDL most potently decreased TNF‐α and increased IL‐10 levels in the supernatant (Figure 5l,m), confirming its superior ability to suppress inflammatory activation while restoring immunoregulatory balance.

To further connect these immunomodulatory effects with the upstream delivery mechanism, we measured the two central inflammatory triggers targeted by this platform, namely LPS and HMGB1. LPS and HMGB1 represent major pathogen‐ and damage‐associated inflammatory drivers, respectively, and together they sustain macrophage hyperactivation during sepsis. Consistent with the LPS‐clearance cascade established in Section 3.7, TCe‐C@rHDL significantly reduced extracellular LPS levels more effectively than the other formulations (Figure 5n). In parallel, Cel‐containing formulations reduced HMGB1 levels, with TCe‐C@rHDL producing the most pronounced suppression (Figure 5o). This is consistent with effective intracellular availability of Cel after delivery, enabling antagonism of HMGB1‐associated signaling. Thus, TCe‐C@rHDL simultaneously intercepts both an upstream endotoxin stimulus and a downstream danger signal, providing a mechanistic explanation for its broad anti‐inflammatory efficacy.

Collectively, these findings show that AEGIS converts preserved anti‐dysfunction performance into multimodal immunoregulation rather than a single‐pathway anti‐inflammatory effect, thereby interrupting the loop of oxidative stress, inflammatory amplification, and immune dysfunction. Within the pathology‐informed workflow, this section defines the pharmacodynamic output of the design and provides the mechanistic basis for subsequent in vivo validation of therapeutic efficacy.

### 3.9 TCe‐C@rHDL Translates Anti‐Dysfunction Design Into Systemic Immunoprotection In Vivo

**FIGURE 6:**
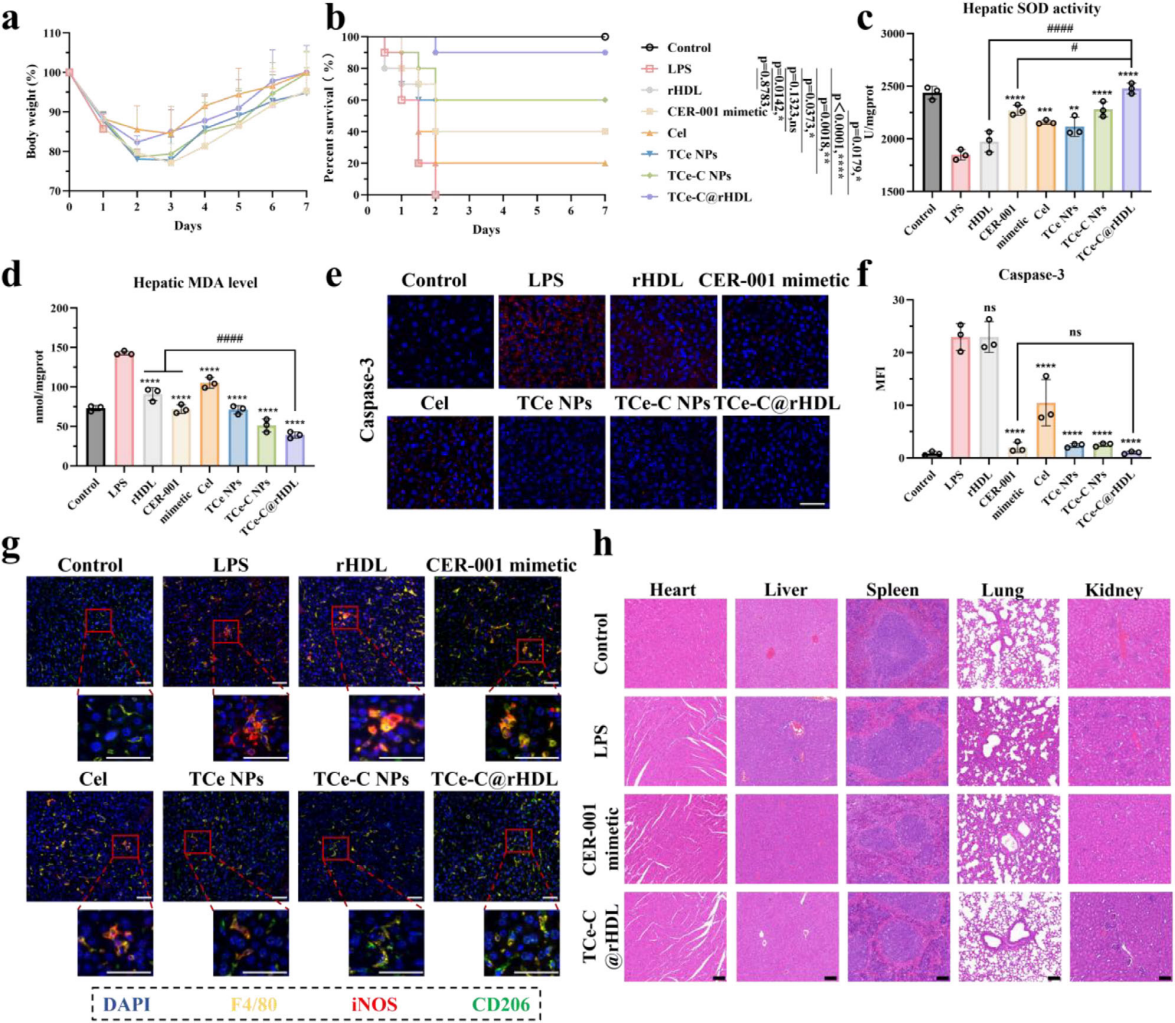
TCe‐C@rHDL translates anti‐dysfunction design into systemic immunoprotection in vivo. (a) Body weight changes over 7 days in LPS‐challenged mice. (b) Survival curves of LPS‐challenged mice over 7 days (*n* = 10). Survival was monitored at 12‐h intervals for the first 48 h and daily thereafter. Statistical significance was analyzed by log‐rank test. (c,d) Hepatic SOD activity and MDA level measured 24 h after LPS challenge (*n* = 3). (e,f) Representative immunofluorescence images and quantification of hepatic caspase‐3 expression in LPS‐challenged mice (*n* = 3). Blue, nuclei; red, caspase‐3. Scale bars, 50 µm. (g) Representative immunofluorescence images of macrophage polarization in liver tissue. Blue, nuclei; yellow, F4/80; red, iNOS; green, CD206. Scale bars, 50 µm. (h) Representative H&E‐stained sections of major organs from LPS‐challenged mice. Scale bars, 100 µm. Data are presented as mean ± SD. Symbols above bars denote significance versus the LPS group: ns, not significant; *P < 0.05, **P < 0.01, ***P < 0.001, ****P < 0.0001.

Thereafter, we evaluated TCe‐C@rHDL in an LPS‐induced systemic inflammation model and compared its performance with those of its individual components, TCe–C NPs, blank rHDL, and CER‐001 mimetic. Survival analysis provided direct evidence of therapeutic translation. TCe–C NPs achieved a 60% survival rate, outperforming Cel (20%) and TCe NPs (40%), consistent with the synergistic interaction between the two functional components. Notably, TCe‐C@rHDL further increased survival to 90%, markedly exceeding CER‐001 mimetic (40%) (Figure 6b). In parallel, mice treated with TCe‐C@rHDL recovered body weight more rapidly than those receiving CER‐001 mimetic (Figure 6a), indicating superior restoration of overall physiological status.

We next examined whether this survival benefit was associated with better control of systemic oxidative damage. In the liver, TCe‐C@rHDL significantly increased SOD activity and reduced MDA levels, restoring both markers close to those of the healthy control group and outperforming CER‐001 mimetic as well as blank rHDL (Figure 6c,d). DHE staining further showed that TCe‐C@rHDL most effectively reduced superoxide levels in multiple sepsis‐vulnerable organs, including the liver, kidney, and lung (Figure S21), demonstrating broad antioxidant protection at the whole‐organism level. Besides, immunofluorescence staining for caspase‐3 showed that both Cel and TCe NPs exerted anti‐apoptotic effects in the liver, in agreement with the cellular results, whereas TCe‐C@rHDL produced the strongest suppression of apoptotic signaling, exceeding TCe–C NPs and slightly outperforming CER‐001 mimetic (Figure 6e,f). Given the prominent pulmonary accumulation of TCe‐C@rHDL under inflammatory conditions, TUNEL staining was used to assess apoptosis in lung tissue. TCe‐C@rHDL markedly reduced apoptotic cell numbers in the lung (Figure S22), further supporting its capacity to confer multi‐organ protection. This protection was accompanied by restoration of macrophage immune balance in vivo. Liver sections were analyzed using F4/80 as a macrophage marker together with iNOS and CD206 as markers of M1‐ and M2‐ phenotypes, respectively (Figure 6g). Consistent with the in vitro polarization results, TCe‐C@rHDL most effectively suppressed the pro‐inflammatory macrophage phenotype while promoting a pro‐resolving state, indicating that the platform retains its macrophage‐reprogramming capacity in the inflamed organism.

Furthermore, multi‐organ injury was assessed by H&E staining (Figure 6h; Figure S23). Histopathological examination showed that LPS‐challenged mice developed typical tissue damage, including pulmonary edema and congestion, hepatic inflammatory infiltration, and glomerular edema. In contrast, TCe‐C@rHDL treatment markedly alleviated these pathological alterations and preserved near‐normal tissue architecture across the examined organs, particularly in the lungs, where the histological appearance was comparable to that of the Control group. Consistent with the histological results, blood biochemical analysis further showed that TCe‐C@rHDL restored liver and kidney function markers, including ALT, AST, BUN, and CREA, to near‐normal levels, with significantly greater improvement than CER‐001 mimetic (Figure S24).

Collectively, these results show that TCe‐C@rHDL translates preserved anti‐dysfunction performance into substantial systemic immunoprotection in vivo by suppressing oxidative damage, limiting apoptosis, reprogramming macrophage polarization, and alleviating multi‐organ injury. Notably, its therapeutic efficacy was markedly superior to that of the CER‐001 mimetic, further underscoring the translational value of the AEGIS strategy.

### 3.10 TCe‐C@rHDL Attenuates NF‐κB‐Centered Inflammatory and Pyroptosis‐Associated Signaling

**FIGURE 7:**
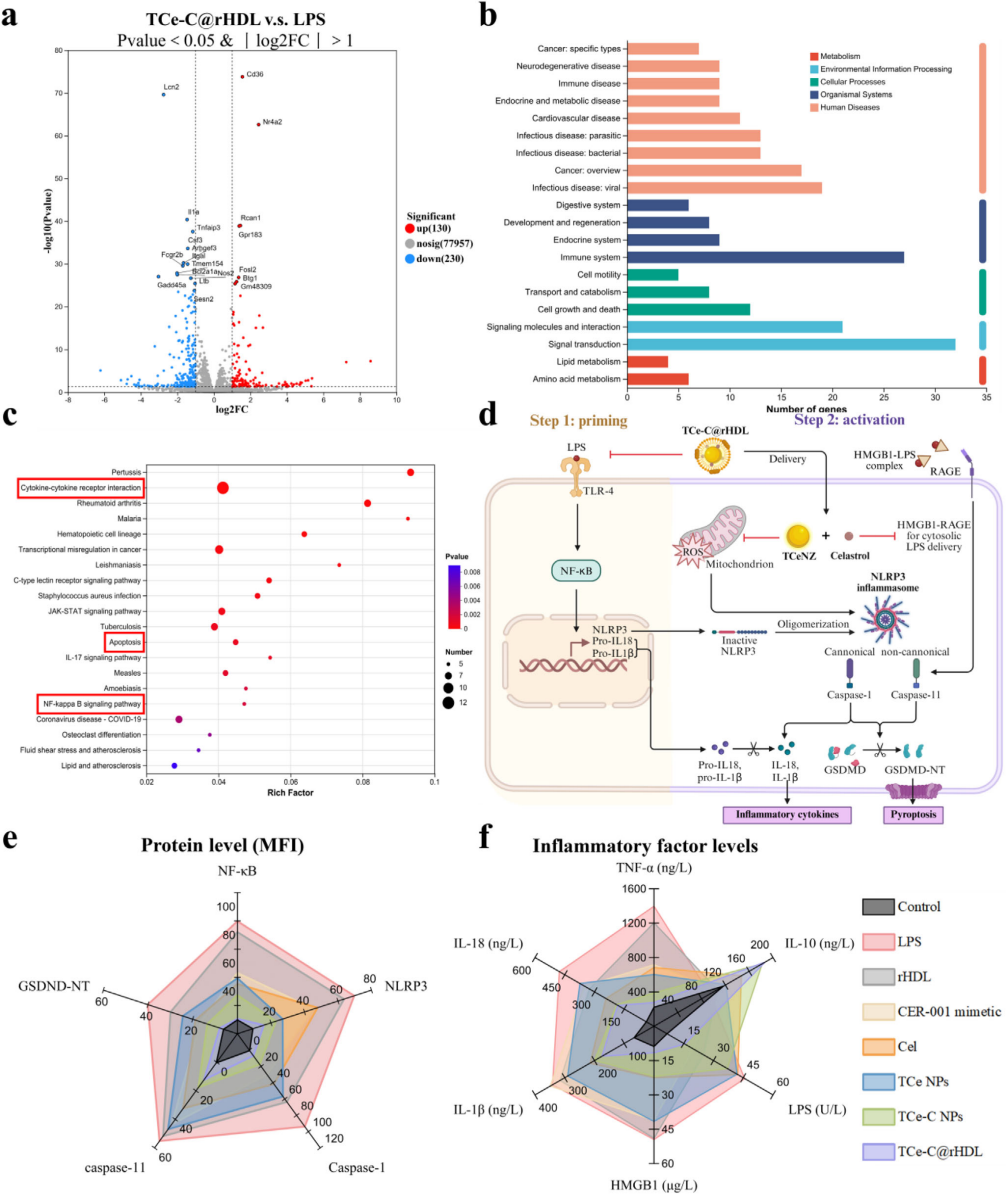
TCe‐C@rHDL attenuates NF‐κB‐centered inflammatory and pyroptosis‐associated signaling. (a) Volcano plot of differentially expressed genes (DEGs) between TCe‐C@rHDL‐treated and untreated LPS‐stimulated RAW264.7 cells. (b) Top 20 KEGG pathway annotation results for DEGs. (c) KEGG pathway enrichment analysis. Dot size and color represent gene ratio and *P‐*value, respectively. Rich factor denotes the ratio of DEG number to total gene number in a given pathway; a higher rich factor indicates stronger enrichment. (d) Schematic illustration of canonical‐ and noncanonical‐pyroptosis‐associated signaling and the proposed points of modulation by TCe‐C@rHDL. (e) Radar plot showing relative fluorescence intensities of key pyroptosis‐related proteins (NF‐κB, NLRP3, caspase‐1, caspase‐11, and GSDMD‐NT) in liver tissue (*n* = 3). (f) Radar plot showing serum levels of inflammatory mediators (TNF‐α, IL‐10, LPS, HMGB1, IL‐1β, and IL‐18) in LPS‐challenged mice (*n* = 3).

To further elucidate the molecular basis of the multimodal immunotherapy mediated by TCe‐C@rHDL, transcriptomic analysis was performed in LPS‐stimulated RAW264.7 cells. Compared with the LPS group, TCe‐C@rHDL treatment altered the expression of 360 differentially expressed genes (130 upregulated and 230 downregulated; Figure 7a), and heatmap analysis revealed a clearly distinct transcriptional profile from that of untreated inflammatory macrophages (Figure S25). KEGG annotation showed that these differentially expressed genes were mainly enriched in pathways related to signal transduction, cellular growth and death, immune system regulation, and infectious diseases (Figure 7b), indicating that TCe‐C@rHDL broadly reprograms macrophage responses rather than affecting a single inflammatory output. More specifically, pathway enrichment analysis demonstrated significant suppression of cytokine–cytokine receptor interaction, apoptosis, and NF‐κB signaling (Figure 7c), suggesting that the platform acts by coordinately attenuating inflammatory amplification, cell death, and immune dysregulation. Among these pathways, NF‐κB appears to serve as the central regulatory hub. As a master transcriptional driver of macrophage activation, NF‐κB is activated by both pathogen‐associated and damage‐associated inflammatory triggers, including LPS and HMGB1, and subsequently induces the expression of pro‐inflammatory cytokines such as TNF‐α, pro‐IL‐1β, and pro‐IL‐18. Therefore, the transcriptomic suppression of NF‐κB provides a molecular explanation for the cellular findings described above, including reduced TNF‐α production, lower LPS‐ and HMGB1‐associated inflammatory burden, and reversal of M1‐dominant macrophage polarization.

Importantly, NF‐κB is functionally coupled to the NLRP3 inflammasome, forming a core signaling axis that links inflammatory priming to pyroptotic execution (Figure 7d) [63]. In addition, oxidative mitochondrial damage further promotes NLRP3 inflammasome assembly, thereby connecting redox stress to inflammatory cell death. Once activated, the NLRP3 inflammasome triggers caspase‐1 activation, which cleaves gasdermin D to generate GSDMD‐NT, the pore‐forming executor of pyroptosis, while simultaneously processing pro‐IL‐1β and pro‐IL‐18 into their mature inflammatory forms. Beyond this canonical pathway, extracellular HMGB1 can facilitate cytosolic LPS entry through RAGE, leading to caspase‐11 activation and non‐canonical pyroptosis [67]. Thus, the inflammatory environment targeted by TCe‐C@rHDL converges on a dual pyroptotic network integrating NF‐κB priming, NLRP3 activation, oxidative stress, and HMGB1/LPS co‐signaling. Such multi‐factorial inflammatory networks also highlight the potential advantage of therapeutic strategies capable of concurrently attenuating multiple inflammatory mediators in sepsis [68].

Consistent with this mechanism, quantitative immunofluorescence analysis of liver tissue from LPS‐challenged mice showed increased signals for NF‐κB, NLRP3, caspase‐1, caspase‐11, and GSDMD‐NT in liver tissue (Figure 7e; Figure S26). The concurrent increase in upstream inflammasome‐associated proteins and the cleavage‐associated GSDMD‐NT fragment is consistent with enhanced pyroptosis‐associated signaling in the LPS‐challenged inflammatory state. Both Cel and TCe NPs reduced the signals of NF‐κB, NLRP3, caspase‐1, and GSDMD‐NT, whereas TCe–C NPs produced a more pronounced overall reduction, consistent with broader attenuation of inflammatory and pyroptosis‐associated signaling. Notably, Caspase‐11‐associated fluorescence was also reduced in the Cel‐containing groups, with the lowest signal observed after TCe‐C@rHDL treatment, suggesting a modulation of noncanonical‐pyroptosis‐associated signaling. Most importantly, TCe‐C@rHDL produced the strongest overall inhibition among all groups. This result is mechanistically consistent with the hierarchical delivery design: by enabling direct cytosolic release of Cel while preserving the antioxidant and mitochondrial‐protective activity of TCeNZ, TCe‐C@rHDL is better positioned to simultaneously suppress both branches of pyroptotic signaling.

Serum cytokine profiling further supported this interpretation (Figure 7f). LPS challenge markedly elevated circulating levels of LPS, HMGB1, TNF‐α, IL‐1β, and IL‐18, reflecting systemic hyperinflammation accompanied by increased inflammasome‐associated cytokine release. TCe‐C@rHDL most effectively reduced circulating LPS, in agreement with its previously established LPS‐clearance cascade, while Cel‐containing formulations substantially lowered HMGB1 levels. In parallel, TCe‐C@rHDL produced the greatest reductions in circulating IL‐1β and IL‐18, providing downstream evidence consistent with attenuation of inflammasome‐associated inflammation. It also produced the most pronounced reduction in TNF‐α and the largest increase in IL‐10, consistent with restoration of macrophage immune balance and a shift from inflammatory amplification toward tissue‐repair signaling. Overall, these results identify the molecular core of TCe‐C@rHDL‐mediated immunotherapy. By simultaneously antagonizing LPS and HMGB1, buffering oxidative stress, and suppressing the NF‐κB‐centered inflammatory‐pyroptotic axis, TCe‐C@rHDL interrupts the feed‐forward loop linking macrophage hyperactivation, apoptosis/pyroptosis, and cytokine storm.

### 3.11 Combining Anti‐Infection Therapy With TCe‐C@rHDL‐Based Immunoregulation Improves Outcomes in the CLP Model

**FIGURE 8:**
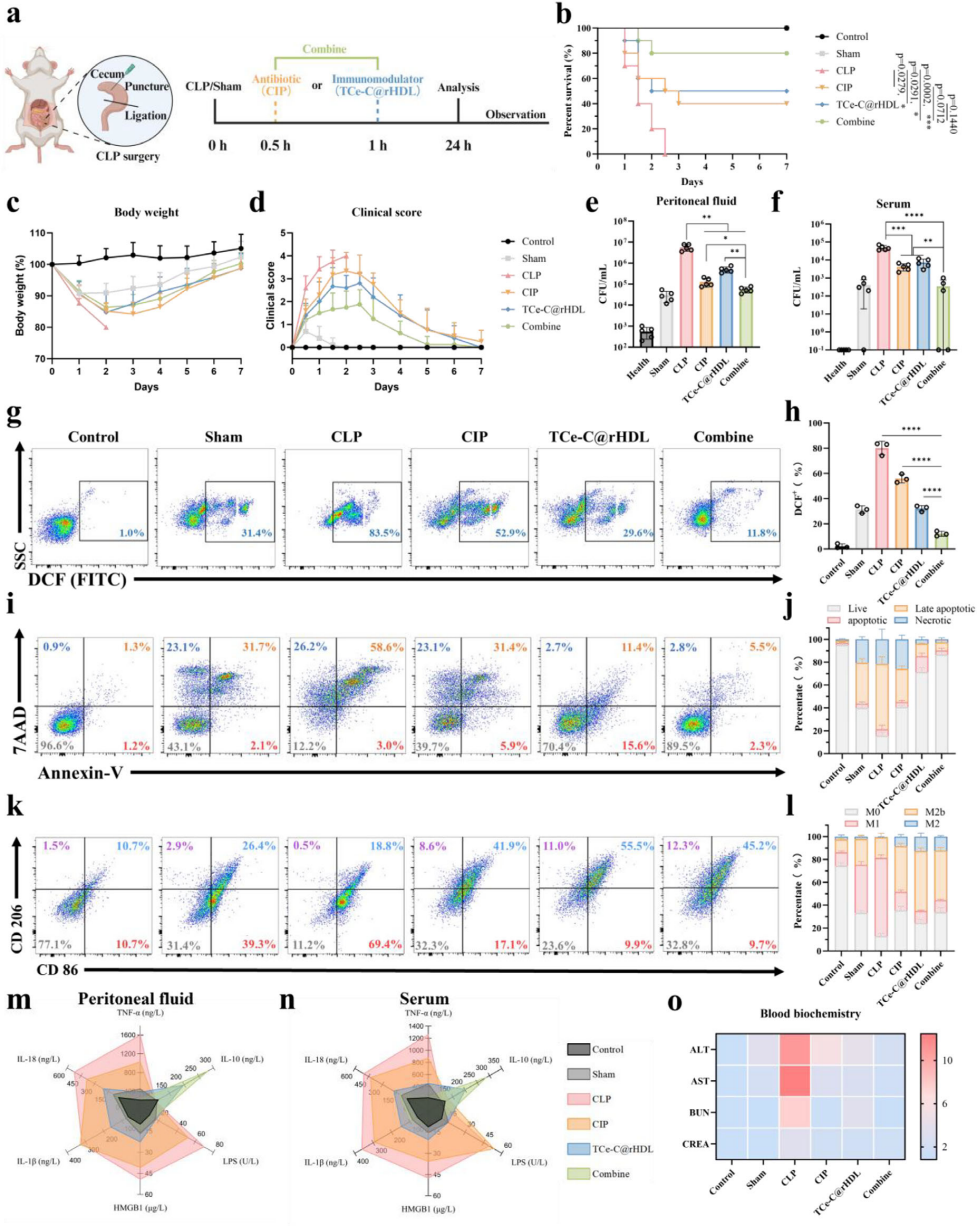
TCe‐C@rHDL‐based immunoregulation complements antibiotic therapy in the CLP model. (a) Schematic illustration of CLP model establishment and treatment schedule. Ciprofloxacin (CIP, 20 mg/kg) or TCe‐C@rHDL (0.75 mg/kg Ce and 0.12 mg/kg Cel) was administered by intraperitoneal injection at 0.5 and 1 h post‐CLP, respectively. The combination group received both treatments at the indicated time points. (b) Survival curves of CLP mice over 7 days (*n* = 10). Survival was monitored at 12‐h intervals for the first 72 h and daily thereafter. Statistical significance was analyzed by log‐rank test. (c,d) Body weight changes and clinical scores over 7 days in CLP mice. (e,f) Bacterial burdens in peritoneal lavage fluid and serum, quantified as CFUs (*n* = 5). “10^−1^” indicates colony counts below the detection limit under the current experimental conditions. (g,h) Flow cytometry analysis and quantification of intracellular ROS levels in peritoneal cells (*n* = 3). (i,j) Flow cytometry analysis and quantification of apoptosis in peritoneal cells (*n* = 3). Quadrants: Annexin V‐/7AAD‐, live cells; Annexin V+/7AAD‐, early apoptotic cells; Annexin V+/7AAD+, late apoptotic cells; Annexin V‐/7AAD+, necrotic cells. (k,l) Flow cytometry analysis and quantification of polarization phenotypes in peritoneal macrophages (gated as CD11b+F4/80+ cells) (*n* = 3). (m,n) Radar plots showing levels of inflammatory mediators (TNF‐α, IL‐10, LPS, HMGB1, IL‐1β, and IL‐18) in peritoneal fluid and serum of CLP mice (*n* = 3). (o) Blood biochemical analysis (*n* = 3). Data are presented as mean ± SD.

The therapeutic benefit of TCe‐C@rHDL in the LPS‐driven systemic inflammation model prompted us to further assess its translational value in CLP, a clinically relevant sepsis model that incorporates polymicrobial infection, hyperinflammation, and multi‐organ injury [69]. Because current sepsis management relies primarily on antimicrobial therapy but does not adequately correct the underlying immune dysregulation [70, 71], we hypothesized that combining antibiotic treatment with TCe‐C@rHDL‐based immunoregulation would provide superior therapeutic benefit to either strategy alone. Ciprofloxacin hydrochloride (CIP), a broad‐spectrum fluoroquinolone antibiotic, was selected as a model active antibacterial comparator because ciprofloxacin‐containing regimens have previously been used in murine CLP studies, including as antibacterial background therapy for evaluating adjunctive immunomodulatory interventions [72, 73] (Figure 8a).

Survival analysis provided direct support for this combined strategy. All mice in the untreated CLP group died within 60 h, confirming the severity of the model. CIP monotherapy improved 7‐day survival to 40%, whereas TCe‐C@rHDL monotherapy achieved a comparable survival rate of 50%, indicating that immunoregulation alone can provide meaningful protection even in an infection‐driven model. Notably, the combination group reached an 80% survival rate, substantially outperforming either monotherapy (Figure 8b). Consistent with this survival benefit, mice receiving combined treatment showed faster body‐weight recovery and the most pronounced reduction in clinical scores, approaching near‐complete recovery by day 5 (Figure 8c,d).

Histological and biochemical analyses further demonstrated that this therapeutic advantage translated into multi‐organ protection. H&E staining showed that CLP induced severe tissue injury, particularly pulmonary edema and red blood cell infiltration in the lungs, whereas all interventions alleviated these pathological changes to varying degrees (Figure S27). However, lungs from the CIP group still displayed thinning and disruption of alveolar walls, suggesting that bacterial control alone could not fully resolve inflammatory tissue damage. By contrast, tissues from the TCe‐C@rHDL and combination groups showed minimal pathological alterations and were much closer to healthy controls. Consistently, CLP markedly elevated serum markers of liver and kidney injury at 24 h, and although both CIP and TCe‐C@rHDL monotherapies reduced these abnormalities, the combination regimen restored biochemical parameters most effectively (Figure 8o). Together, these results indicate that integrating anti‐infection therapy with immunoregulation yields the most robust protection against sepsis‐associated organ dysfunction.

### 3.12 Combined Anti‐Infection and Immunoregulation Improve Immune Restoration in the CLP Model

To further clarify why the combined regimen outperformed either monotherapy in the CLP model, we next examined its effects on bacterial burden, cellular immune status, and inflammatory mediator profiles. We first quantified bacterial burden in the peritoneal cavity and blood by colony‐forming unit (CFU) analysis (Figure 8e,f; Figure S28). As expected, CIP monotherapy reduced bacterial counts in both compartments by approximately 1–2 log units. Notably, TCe‐C@rHDL monotherapy also significantly lowered bacterial burden, with blood CFU levels comparable to those in the CIP group. This finding suggests that immunoregulation itself can facilitate bacterial clearance, likely by improving the functional state of immune cells. Most importantly, the combination group exhibited the greatest reduction in bacterial load in both peritoneal fluid and blood, with bacteria becoming undetectable in the blood of two mice.

We next examined whether this improved pathogen clearance was accompanied by restoration of cellular immune status. Flow‐cytometric analysis of peritoneal cells showed that CLP induced marked oxidative stress, with approximately 80% of cells positive for DCF fluorescence (Figure 8g,h). CIP treatment partially reduced this proportion to about 55%, indicating that bacterial control alone was insufficient to normalize intracellular oxidative stress. By contrast, TCe‐C@rHDL reduced ROS‐positive cells to approximately 30%, consistent with its potent antioxidant activity. The combination group showed the strongest effect, lowering the DCF‐positive population to about 12%.

A similar trend was observed for apoptosis. CLP caused extensive necrosis and late apoptosis in peritoneal cells (Figure 8i,j). Although CIP monotherapy alleviated this injury and restored apoptosis levels close to those of the Sham group, TCe‐C@rHDL exerted a stronger anti‐apoptotic effect, markedly reducing necrotic and late‐apoptotic cells while slightly increasing the early‐apoptotic fraction, possibly by delaying progression toward irreversible cell death. Again, the combination group showed the greatest protection, with the lowest levels of apoptosis/necrosis and the highest proportion of viable cells, reaching approximately 90%.

Macrophage polarization analysis further highlighted the complementarity of the two treatments (Figure 8k,l). Compared with CIP, TCe‐C@rHDL more effectively reduced M1 macrophages while increasing M2 and M2b subsets, confirming its capacity to shift macrophages toward a reparative and highly phagocytic phenotype. However, because M2b macrophages are not the principal bactericidal effector subset and may increase susceptibility to opportunistic infection under persistent pathogen exposure [74], macrophage repolarization alone may not be sufficient in the CLP setting. Consistent with this notion, combination therapy slightly increased the M2 population while reducing M2b cells by approximately 10% and increasing M0 cells by approximately 10% relative to TCe‐C@rHDL monotherapy, suggesting a partial rebalancing away from excessive M2b skewing. Together with the reduced bacterial burden, these results imply that antibiotic treatment helps prevent infection‐driven maladaptive immune polarization, allowing immunoregulation to promote a more favorable restoration of immune homeostasis.

Finally, inflammatory mediator levels in peritoneal fluid and serum were measured to evaluate the overall inflammatory status (Figure 8m,n). CLP markedly increased the levels of LPS, HMGB1, TNF‐α, IL‐1β, and IL‐18. Both CIP and TCe‐C@rHDL monotherapies reduced these pro‐inflammatory factors, but with distinct emphases: CIP primarily acted through pathogen reduction, whereas TCe‐C@rHDL more effectively relieved inflammatory amplification and additionally increased IL‐10, which is associated with post‐inflammatory resolution and tissue repair. Notably, the combination group produced the most pronounced overall normalization of inflammatory mediators, with cytokine levels closest to those of healthy controls for all measured factors except LPS.

Overall, CIP primarily lowers pathogen burden, whereas TCe‐C@rHDL mitigates oxidative stress, apoptosis, inflammatory amplification, and macrophage dysfunction. Their combination therefore interrupts both arms of the infection‐inflammation vicious cycle, leading to improved bacterial clearance and more effective restoration of immune homeostasis than either monotherapy alone. These findings strengthen the translational significance of this work by showing that TCe‐C@rHDL is particularly well suited to function as an immunoregulatory partner in combination sepsis therapy.

## 4 Conclusion

In conclusion, this study demonstrates that biomimetic dysfunction can be addressed prospectively through pathology‐informed design. Using rHDL as a model, we identified representative sepsis‐relevant dysfunction liabilities and developed the AEGIS strategy, which integrates nanozyme armoring, microenvironment intervention, and preservation of HDL‐intrinsic delivery routing. Unlike our previous antioxidant HDL strategies, which primarily mitigated oxidation‐induced dysfunction by retaining endogenous protective components or introducing cascade antioxidants [31, 32], AEGIS extends protection to multiple sepsis‐associated liabilities, including oxidative injury, impaired LPS handling, and SAA‐associated apoA‐I remodeling, while interrupting the LPS/HMGB1‐driven inflammatory amplification loop and enabling hierarchical cytoplasmic and mitochondrial delivery. This integrated anti‐dysfunction design translated preserved biomimetic function into robust immunotherapeutic effects, including attenuation of oxidative stress and cell death, reprogramming of macrophage polarization, 90% survival in LPS‐induced systemic inflammation versus 40% for the CER‐001 mimetic, and 80% survival in the CLP model when combined with antibiotics versus 40% for antibiotics alone.

At the same time, several limitations should be acknowledged. First, although the study moved beyond simplified buffer systems by incorporating patient serum, physiologically relevant shear stress, and in vivo inflammatory models, validation in a larger, prospectively recruited and clinically characterized cohort with appropriate healthy‐donor controls will be necessary to assess inter‐patient variability, define associations with disease severity and biochemical features, and establish the broader clinical generalizability of these findings. Second, because clinical‐grade CER‐001 and CSL‐112 materials were not available, our comparative studies relied on in‐house mimetics prepared according to published compositions and protocols. Although these mimetics provide a rational and informative benchmark, subtle differences in composition, assembly quality, apolipoprotein conformation, or batch‐level physicochemical features may remain relative to the original clinical formulations. Accordingly, the present comparisons should be interpreted as demonstrating superiority over mimetic reconstructions of clinical candidates, rather than as a definitive head‐to‐head comparison against authenticated clinical products. Third, future studies should evaluate TCe‐C@rHDL in combination with more clinically representative broad‐spectrum antimicrobial regimens and include matched antibiotic PK/PD analyses to determine whether the nanoplatform alters antibiotic exposure or pharmacodynamic activity.

Despite these limitations, the broader implication of this work lies not only in advancing AEGIS as an anti‐dysfunction strategy for rHDL therapeutics, but also in establishing a pathology‐informed identification‐design‐evaluation‐validation workflow for biomimetic systems. Successful biomimetic design, in this view, should not be judged only by baseline resemblance to endogenous counterparts, but by whether function can be prospectively designed, screened, and validated under pathological stress. We anticipate that this framework may be extended beyond rHDL to other pathology‐sensitive biomimetic systems, including membrane‐coated nanoparticles, lipoprotein‐inspired carriers, and living or semi‐living therapeutic platforms, thereby helping shift the field from static biomimicry toward pathology‐resilient biomimetic engineering.

## Author Contributions

**Han Zhou**: conceptualization, methodology, data curation, investigation, writing – original draft, visualization. **Wenli Zhang**: supervision, writing – review and editing, conceptualization, funding acquisition, project administration, resources. **Guangyu Dai**: data curation. **Hanming Wang**: data curation. **Xiancheng Chen**: resources, project administration, writing – review and editing. **Jianping Liu**: funding acquisition. **Xue Chen**: data curation. **Xingyu Cai**: validation, formal analysis, data curation. **Lei Zhang**: data curation, formal analysis, validation. **Jia Hua**: data curation. **Jiajia Su**: data curation. **Chunmeng Sun**: project administration, writing – review and editing, resources. **Erhao Wang**: resources, project administration, writing – review and editing, funding acquisition.

## Conflicts of Interest

The authors declare no conflicts of interest.

## Supporting information




**Supporting File 1**: advs77714‐sup‐0001‐S1.mp4.

**Supporting File 2**: advs77714‐sup‐0002‐SuppMat.pdf.

## Data Availability

The data that support the findings of this study are available from the corresponding author upon reasonable request.
