# Supplementary material for: Beyond Biomimetics: Pathology‐Informed Engineering Rescues Reconstituted HDL From Inflammatory Dysfunction for Sepsis Immunotherapy
Source: Adv Sci (Weinh). 2026 Sep 10:e77714. Online ahead of print. doi: 10.1002/advs.77714 (PMC13562793; doi:10.1002/advs.77714)
Supplement: Supplementary file 1 [file ADVS-9999-e77714-s002.pdf]

Supplementary information

Beyond biomimetics: pathology-informed engineering rescues reconstituted HDL from inflammatory dysfunction for sepsis immunotherapy

Han Zhou[1], Lei Zhang[1], Xingyu Cai[1], Jiajia Su[1], Guangyu Dai[1], Xue Chen[1], Hanming Wang[1], Jia Hua[1], Jianping Liu[1], Chunmeng Sun[1,*], Xiancheng Chen[3,*], Erhao Wang[2,*], Wenli Zhang[1,*]

[1] Department of Pharmaceutics, China Pharmaceutical University, Jiangsu 211198, P. R. China

[2] Hainan Women and Children's Medical Center, Haikou 571100, P. R. China

[3] Department of Critical Care Medicine, Nanjing Drum Tower Hospital, The Affiliated Hospital of Nanjing University Medical School, Jiangsu 210008, P. R. China

[*] Correspondence: zwllz@163.com (W. Zhang); hyffy742@muhn.edu.cn (E. Wang); chenxiancheng-icu@foxmail.com (X. Chen); suncmpharm@cpu.edu.cn (C. Sun)

Materials and Methods

Reagents

Cel was purchased from Beitrenkang Biopharmaceutical Technology Co., Ltd (Beijing, China). Cerium (III) acetate hydrate, TPP, and oleylamine were purchased from Aladdin Biochemical Technology Co., Ltd (Shanghai, China). L-alpha-Dipalmitoyl phosphatidylcholine (DPPC), 1,2-distearoyl-sn-glycero-3-phosphoethanolamine-N-[methoxy (polyethylene glycol)-2000] (DSPE-PEG_{2000}) were purchased from AVT Pharmaceutical Tech Co., Ltd (Shanghai, China). apoA-I was extracted according to the methods reported by the laboratory[1]. Human-derived high-density lipoprotein was purchased from Yeasen Biotechnology Co., Ltd (Shanghai, China). CER-001 and CSL-112 mimetics were prepared respectively according to the preparation methods reported in the existing literatures[2]. The reported CER-001 formulation comprises recombinant human apoA-I complexed with egg sphingomyelin and DPPG at a 97:3 phospholipid weight ratio and a protein-to-phospholipid ratio of 1:2.7 (w/w) [1], whereas CSL112 is based on plasma-derived human apoA-I reconstituted with phosphatidylcholine to form discoidal rHDL particles.

SOD assay kit, CAT assay kit, and micro-MDA assay kit were purchased from KeyGEN BioTECH Co., Ltd (Jiangsu, China). Protein carbonyl content determination kit was purchased from Elabscience Biotechnology Co., Ltd (Wuhan, China). Fluorescein isothiocyanate-labeled LPS (FITC-LPS) was purchased from Ruixi Biological Technology Co., Ltd (Xi'an, China). SAA was purchased from Lvdu Bio-technique Industry (Shandong, China). Limulus amebocyte lysate (LAL) was purchased from Beyotime Biotechnology Co., Ltd (Shanghai, China). Adenosine triphosphate (ATP), coumarin 6 (C6), rhodamine B isothiocyanate (RBITC), Nile red, 3- (4,5-dimethylthiazol-2-yl)-2,5-diphenyltetrazolium bromide (MTT), and 2',7'-Dichlorodihydrofluorescein diacetate (DCFH-DA) were purchased from Macklin Biochemical Co., Ltd (Shanghai, China).

Cell culture vessels, DMEM-H medium were purchased from KeyGEN BioTECH Co., Ltd (Jiangsu, China). Fetal bovine serum (FBS) was purchased from ExCell Biotechnology Co., Ltd (Suzhou, China). LPS (form *Escherichia coli* O111:B4) was purchased from Sigma-Aldrich Corporation (American). Apoptosis detection kits and 1,1'-dioctadecyl-3,3,3',3'-tetramethylindotricarbocyanine iodide (DIR) were purchased from Yeasen Biotechnology Co., Ltd

(Shanghai, China). Live cell Caspase-3 activity and mitochondrial membrane potential detection kit, 2-(4-amidinophenyl)-1H-indole-6-carboxamidine (DAPI), Mito-Tracker Deep Red FM, 1,1'-dioctadecyl-3,3,3',3'-tetramethylindocarbocyanine perchlorate (DiI), 3,3'-dioctadecyloxacarbocyanine perchlorate (DiO), chloroquine, and methyl-β-cyclodextrin (mβCD) were purchased from Beyotime Biotechnology Co., Ltd (Shanghai, China). BLT-1 was purchased from Proteintech Group, Inc (Wuhan, China). Z-Phe-Phe-Phe-OH was purchased from GuoPing Pharmaceutical Co., Ltd (Anhui, China). Chlorpromazine (CPZ) and 5- (N- ethyl- N- isopropyl)-Amiloride (EIPA) were purchased from Aladdin Biochemical Technology Co., Ltd (Shanghai, China).

Alexa Fluor 680-conjugated TLR4 antibody was purchased from Santa Cruz Biotechnology, Inc (American). APC Anti-mouse CD86 antibody, PE Anti-mouse CD206 antibody, FITC Anti-mouse/human CD11b antibody, Percp/Cy5.5 Anti-mouse F4/80 antibody, and Anti-mouse CD16/CD32 antibody were purchased from Elabscience Biotechnology Co.,Ltd (Wuhan, China). All Mouse Elisa test kits, F4/80 rabbit mAb, iNOS rabbit mAb, CD206 rabbit mAb, NF-κB rabbit mAb, NLRP3 rabbit mAb, Caspase-1 rabbit mAb, Caspase-3 rabbit mAb, GSDMD-NT rabbit mAb, and Alexa Fluor-conjugated secondary antibodies were purchased from Dream Biotechnology Co.,Ltd (Nanjing, China). Caspase-4/11 rabbit mAb was purchased from Affinity Biosciences Ltd (Jiangsu, China). Ciprofloxacin hydrochloride (CIP) was purchased from MedChemexpress Biotechnology Co.,Ltd (American).

Synthesis and Characteristic of TCeNZ

TCeNZ were synthesized via the reverse micelle method[3]. Initially, 0.43 g of cerium (III) acetate hydrate, 1.05 g of TPP, 2.14 g of oleylamine, and 15 mL of xylene were added to a round-bottom flask. The mixture was then heated to 90 °C under an argon atmosphere with stirring for 32 h. After that, 1 mL of deionized water was rapidly injected into the solution. The reaction mixture was aged at 90°C for 3 hours. After cooling to room temperature, the solution was combined with 50 mL of ethanol and centrifuged to discard the supernatant. Finally, the obtained precipitate was dispersed in 10 mL of chloroform for storage or further use. The surface elemental composition and cerium (Ce) oxidation states of TCeNZ were analyzed using XPS (K-Alpha,

Thermo Fisher, American). Infrared spectra of TCeNZ were acquired using an FTIR spectrometer (TENSOR27, Bruker, German).

Synthesis and Characteristic of TCeNZ-Celastrol Complex (TCe-C)

Synthesis of TCe-C

Cel (0.5 mg) and TCeNZ nanoparticles (2 mg) were combined in 2 mL chloroform. The mixture was stirred for 2 hours to form the TCe-C.

Characteristic of TCe-C

Coordination Bond Formation Analysis: Varying amounts of TCeNZ were added to 1 mL Cel solution (0.8 mM) to achieve Ce: Cel molar ratios of 0, 1:12, 1:6, 1:3, 1:2, 1:1, 2:1, and 3:1. The solutions were photographed, and their UV-Vis spectra were recorded using a UV-Vis spectrophotometer (UV1800, Qinghua Technology Instrument Co., China).

Coordination Bond Dissociation Analysis: Cel was mixed with TCeNZ (Ce: Cel= 1:1). Then, ATP solution was injected to achieve final ATP concentrations of 100 nM, 1 μM, 10 μM, 100 μM, 250 μM, 500 μM, and 1 mM. The solutions were mixed, photographed, and their UV-Vis spectra were recorded. A similar protocol was used for the response experiment on coordination bonds using EDTA (1 mM) as a competitive binding reagent.

Structural Characterization: Cel, TCe-C, and ATP-treated TCe-C were dried under vacuum at room temperature. FTIR and Raman spectra (LabRAM HR, HORIBA Scientific, Japan) of the dried samples were recorded.

Preparation of TCe-C NPs and TCe-C@rHDL

Preparation of TCe-C NPs and TCe-C@rHDL

5 mg DPPC and 5 mg DSPE-PEG$_{2000}$ were dissolved in the previously prepared TCe-C in chloroform. The organic solvent was removed by rotary evaporation (60 °C, vacuum) to form a thin, homogeneous lipid film. Subsequently, the film was hydrated with 3 mL of deionized water. The resulting dispersion was sonicated using a probe sonicator (400 W output, pulse cycle: 2 s on / 3 s off) for 5 minutes to form a clear dispersion. TCe-C NPs were obtained by filtering through

a 0.22 μm polyethersulfone membrane.

An appropriate volume of 10× PBS solution was added to the TCe-C NPs dispersion to achieve a final 1× PBS concentration. 4.0 mg apoA-I powder was added, and the mixture was stirred at 4 °C for 12 hours to facilitate the incorporation of apoA-I. The TCe-C@rHDL was obtained by filtering through a 0.22 μm polyethersulfone membrane.

Characteristic of TCe-C NPs and TCe-C@rHDL

Particle size, Zeta potential, and morphology: The size distribution and zeta potential of nanoparticles was measured by dynamic light scattering (DLS, NanoBrook, Brookhaven instruments, American). The morphology and size of nanoparticles were characterized by transmission electron microscope (TEM, Hitachi HT7700, Japan).

Encapsulation efficiency and drug loading: Lyophilized nanoparticle samples were thoroughly dissolved in ethanol (for Cel quantification) or digested in aqua regia (for cerium quantification). Cel concentration was determined by Ultra-Performance Liquid Chromatography (UPLC, Shimadzu, Japan). Ce ion concentration was determined by Inductively Coupled Plasma Mass Spectrometry (ICP-MS, iCAP™ RQ, Thermo Fisher Scientific, USA).

Stability assessment: Formulations were stored at 4 °C or in PBS containing 10% FBS at 37 °C. The hydrodynamic diameter and polydispersity index (PDI) were measured at predetermined time points to evaluate formulation stability.

In vitro drug release: The release profile of Cel from the formulations was determined using a dialysis method in PBS or PBS containing 10% FBS at 37 °C. the cumulative release rate of Cel was calculated according to the following formula:

$$\text{Cumulative release rate} = (\mathrm{C_i} \times V_1 + \sum_{\mathrm{k}=1}^{i-1} \mathrm{C_k} \times V_2)/(C_I \times V_3) \times 100\%$$

wherein, C_i and C_k are the drug concentration in the release medium at different time points; i = 1, 2, 3, …; k = 1, 2, …, i-1; V_1 is the volume of the released medium; V_2 is sampling volume; and C_I is the initial Cel concentration in the formulations before dialysis; V_3 is the total volume of the sample.

Safety Evaluation of TCe-C@rHDL

In vitro Hemocompatibility Assay

Test samples, 2% (v/v) red blood cell suspension, and normal saline were mixed and incubated

at 37 °C for 2 hours. The mixtures were then centrifuged at 3000 rpm for 10 minutes. The absorbance of the supernatant was measured at 540 nm using a multifunctional microplate reader.

Cell Safety Assessment

RAW 264.7 cells were seeded in 96-well plates at a density of 1×10^5 cells/well and cultured for 24 hours. The culture medium was replaced with fresh medium containing different formulations and incubated for 24 hours. After treatment, cells were washed with PBS and incubated with MTT solution (0.5 mg/mL) for 4 hours. Then, the medium was removed, and dimethyl sulfoxide (DMSO) was added to dissolve the formazan crystals. The absorbance was measured at 570 nm using a multifunctional microplate reader. Cell viability (%) was calculated relative to untreated control cells.

In vivo Safety Assessment in Mice

ICR mice were randomly divided into 6 groups and received i.v. injections of following respective formulations: PBS, TCe@rHDL (Ce 0.75 mg/kg), TCe@rHDL (Ce 1.5 mg/kg), TCe-C@rHDL (Ce 0.75 mg/kg + Cel 0.08 mg/kg), TCe-C@rHDL (Ce 0.75 mg/kg + Cel 0.12 mg/kg), and TCe-C@rHDL (Ce 0.75 mg/kg + Cel 0.18 mg/kg). 72 hours post-injection, blood samples were collected from each mouse. The complete blood counts, including red blood cell count (RBC), white blood cell count (WBC), platelet count (PLT), lymphocyte percentage (Lym%), monocyte percentage (Mon%), and neutrophil percentage (Neu%), were performed using a veterinary fully automatic hematology analyzer (BC-5000Vet, Mindray, China). The remaining blood was centrifuged (4 °C, 3000 rpm) for 10 minutes. The supernatant was collected for blood biochemical analysis through a blood biochemical analyzer (Modular P800, Roche, Switzerland). Following blood collection, mice were euthanized. The heart, liver, spleen, lungs, and kidneys were harvested and weighed. The organ coefficient (%) was calculated for each organ. Finally, the organs were fixed in paraformaldehyde, processed for hematoxylin-eosin (H&E) staining, and their morphology was examined under an optical microscope.

Cytoprotection Evaluation in Inflammation and Oxidative Stress Models

RAW 264.7 cells were allocated to: a) Negative control (untreated cells); b) Positive controls: 800 μM H_2O_2 (oxidative stress) and 2.5 μg/mL LPS (inflammatory stress); c) Intervention groups: treated with Cel, TCe NPs, TCe-C NPs, TCe-C@rHDL, or rHDL using two regimens including

treatment before stimulation (TBS) and treatment post stimulation (TPS). In the TBS regimen, cells were incubated with respective formulations for 2 h prior to stressor administration (H_2O_2 or LPS). Cells were then cultured for an additional 24 h. In the TPS regimen, cells were first exposed to stressors for 2 h, followed by formulation addition and 24 h incubation. The final concentration of Cel and Ce were 0. 063, 0. 125, 0. 25 μM and 0.94, 1.88, 3.75 μM, respectively. rHDL was diluted at equivalently gradient. Finally, cell viability was quantified by MTT assay. The synergistic interaction between Cel and TCeNZ was quantitatively evaluated using the combination index (CI) method based on the median-effect principle of Chou and Talalay.

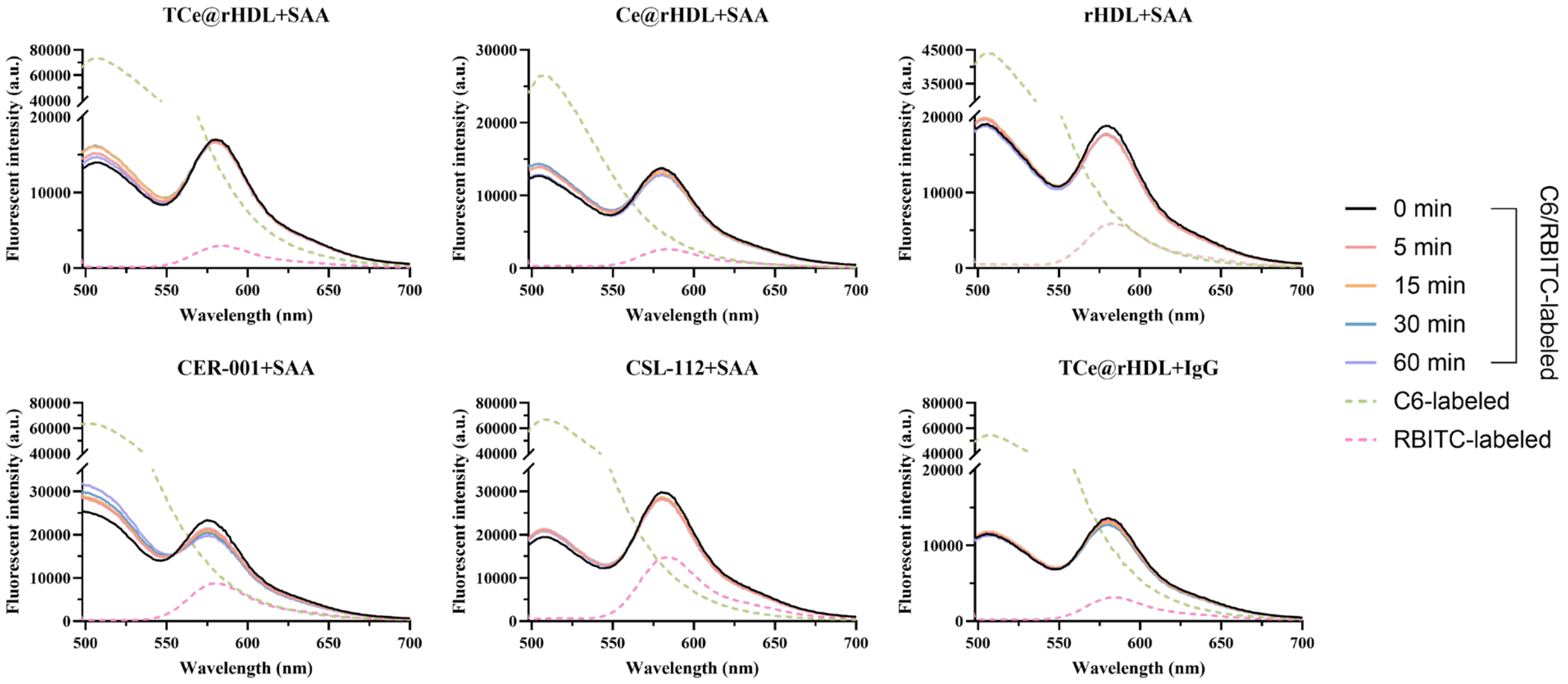

Figure S1. Fluorescence emission spectra of different rHDL formulations incubated with 100 μg/ml SAA within 1 h. The solid lines are C6/RBITC-labeled formulations, the green dashed lines are C6-labeled formulations, and the rose dashed lines are RBITC-labeled apoA-I.

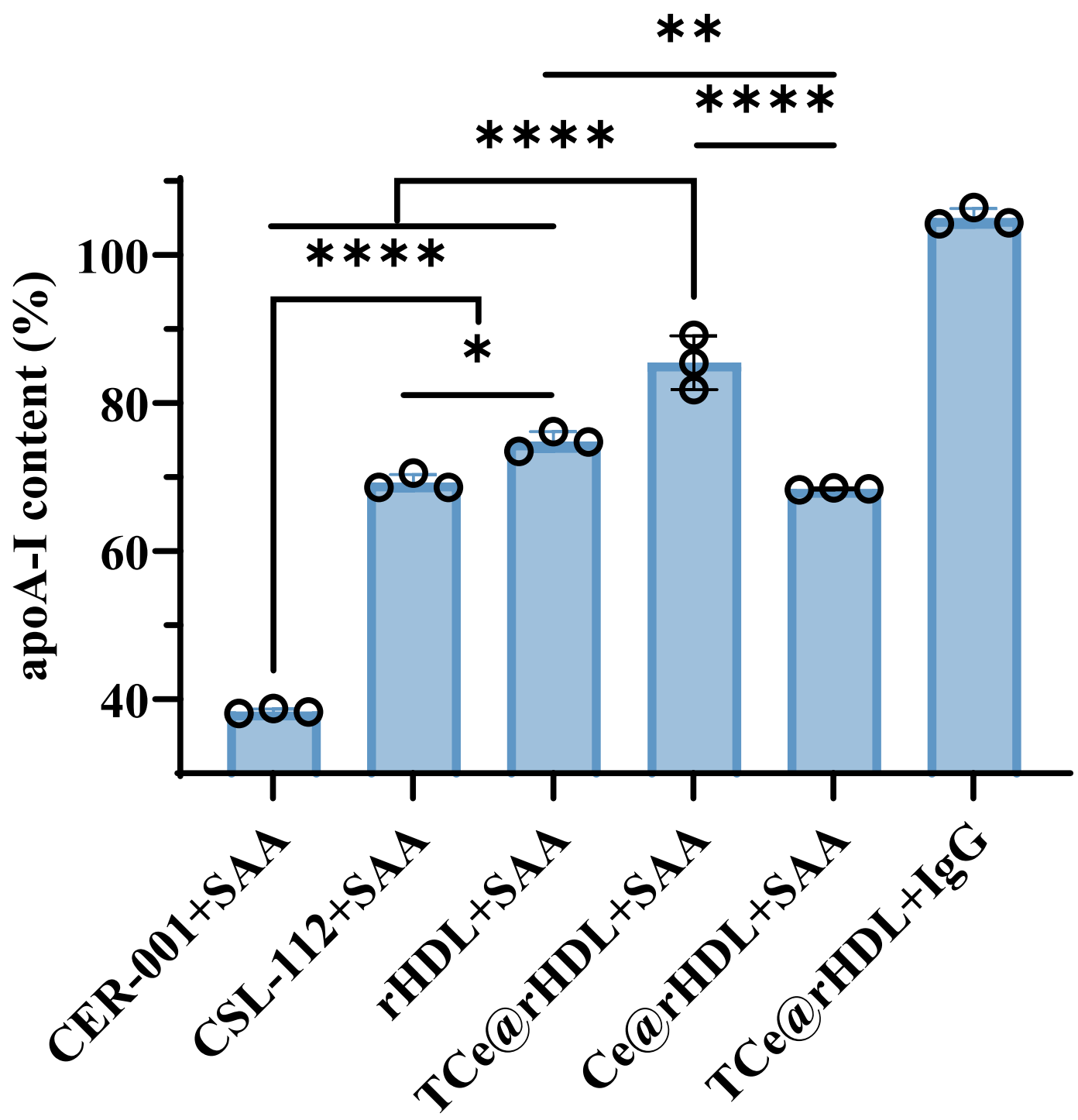

Figure S2. Ultrafiltration-based quantification of particle-associated apoA-I after SAA challenge. RBITC-apoA-I single-labeled rHDL formulations were incubated with SAA (100 µg/mL) for 1 h and subjected to 100-kDa MWCO ultrafiltration. Fluorescence of the retained nanoparticle-associated fraction was quantified and normalized to the corresponding 0-min value (mean ± SD, n = 3).

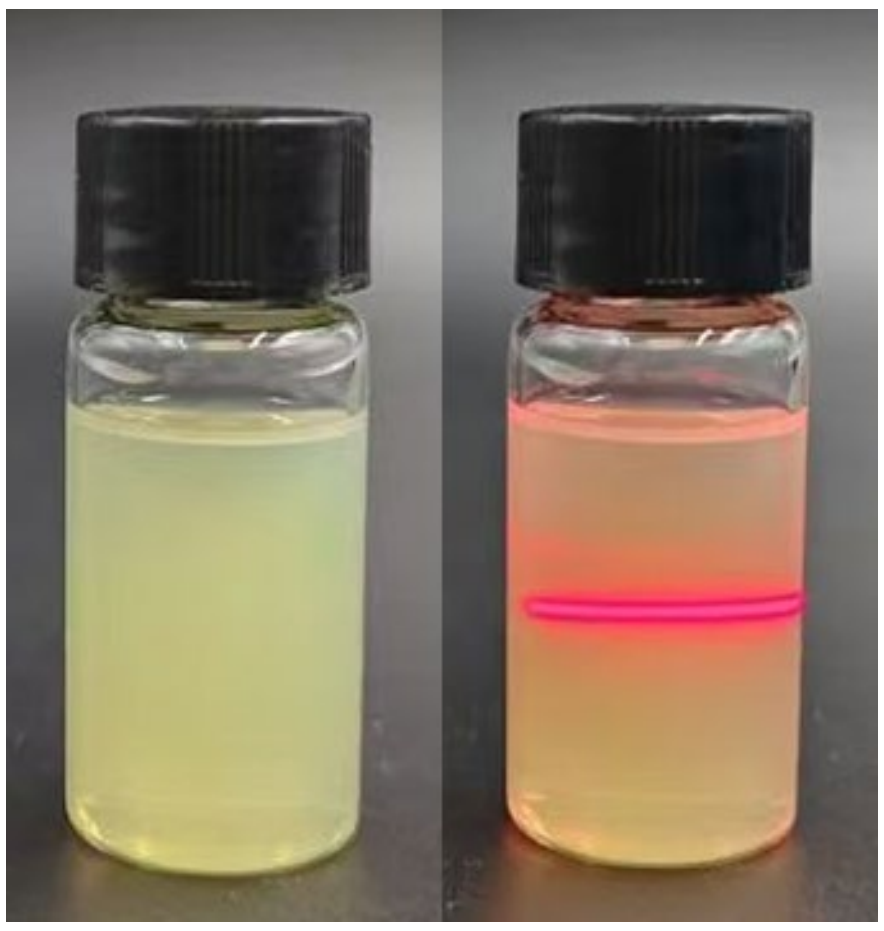

Figure S3. The appearance and Tyndall effect of TCeNZ.

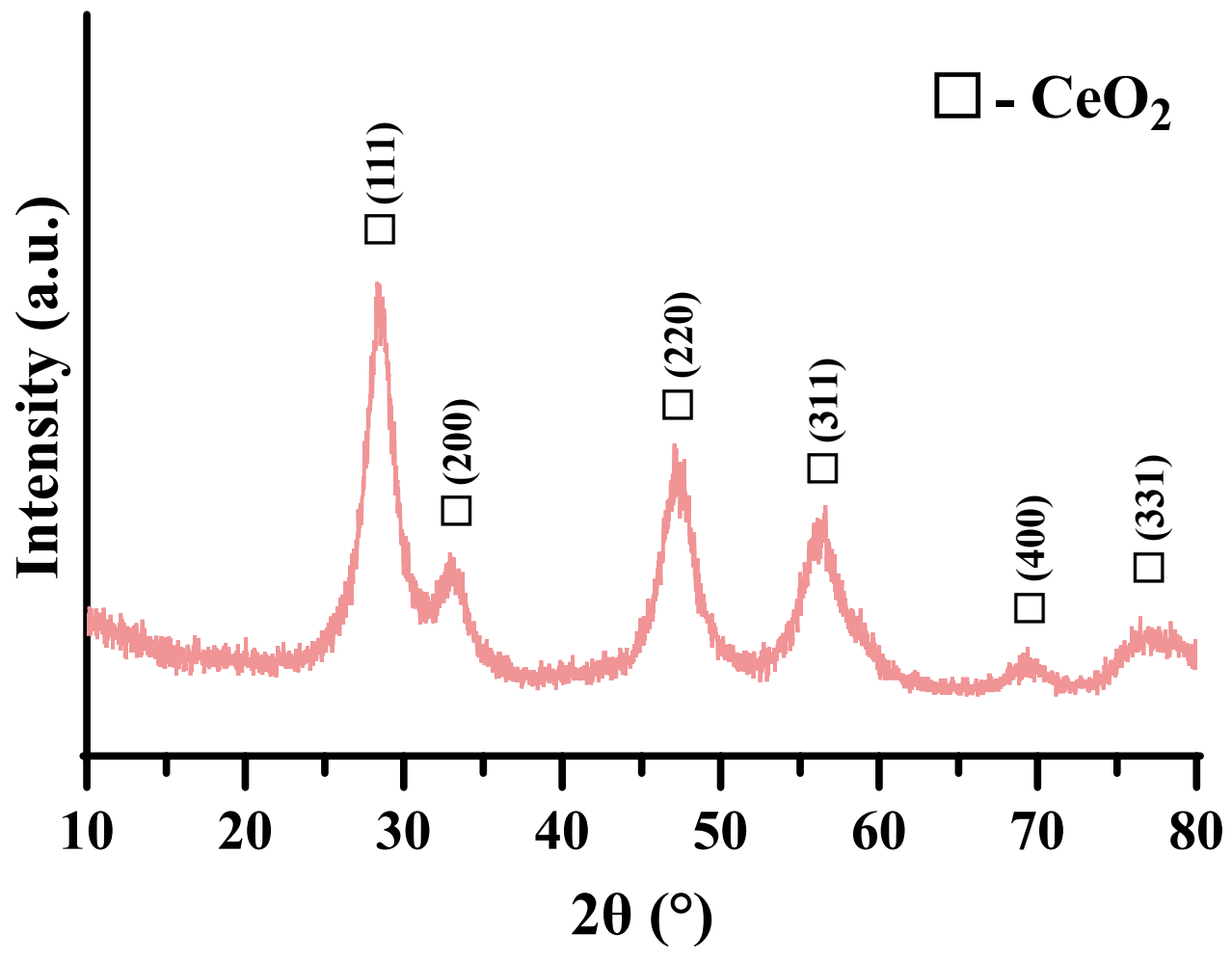

Figure S4. X-Ray diffraction (XRD) pattern of TCeNZ

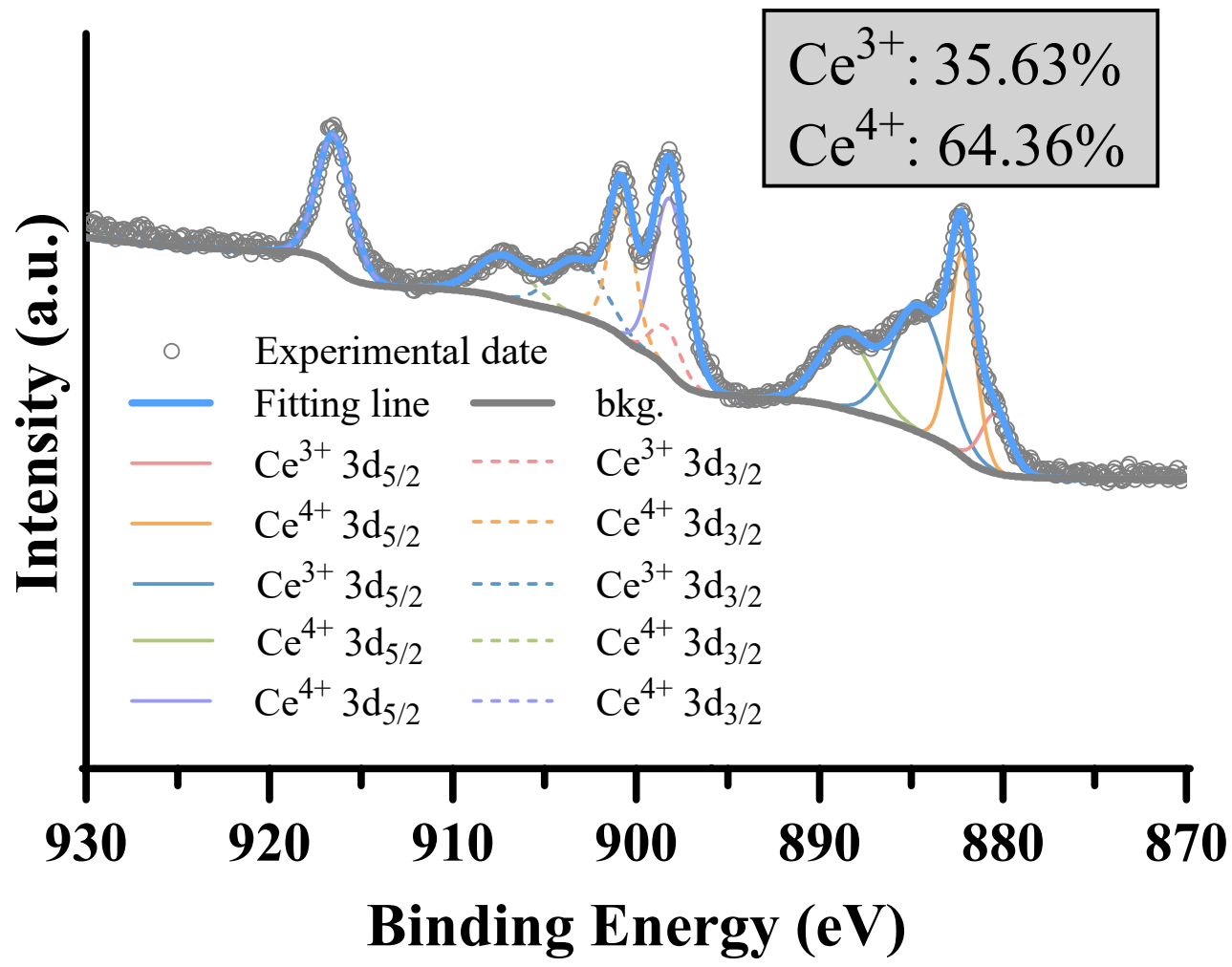

Figure S5. High-resolution Ce 3d XPS spectrum of TCeNZ (870–930 eV).

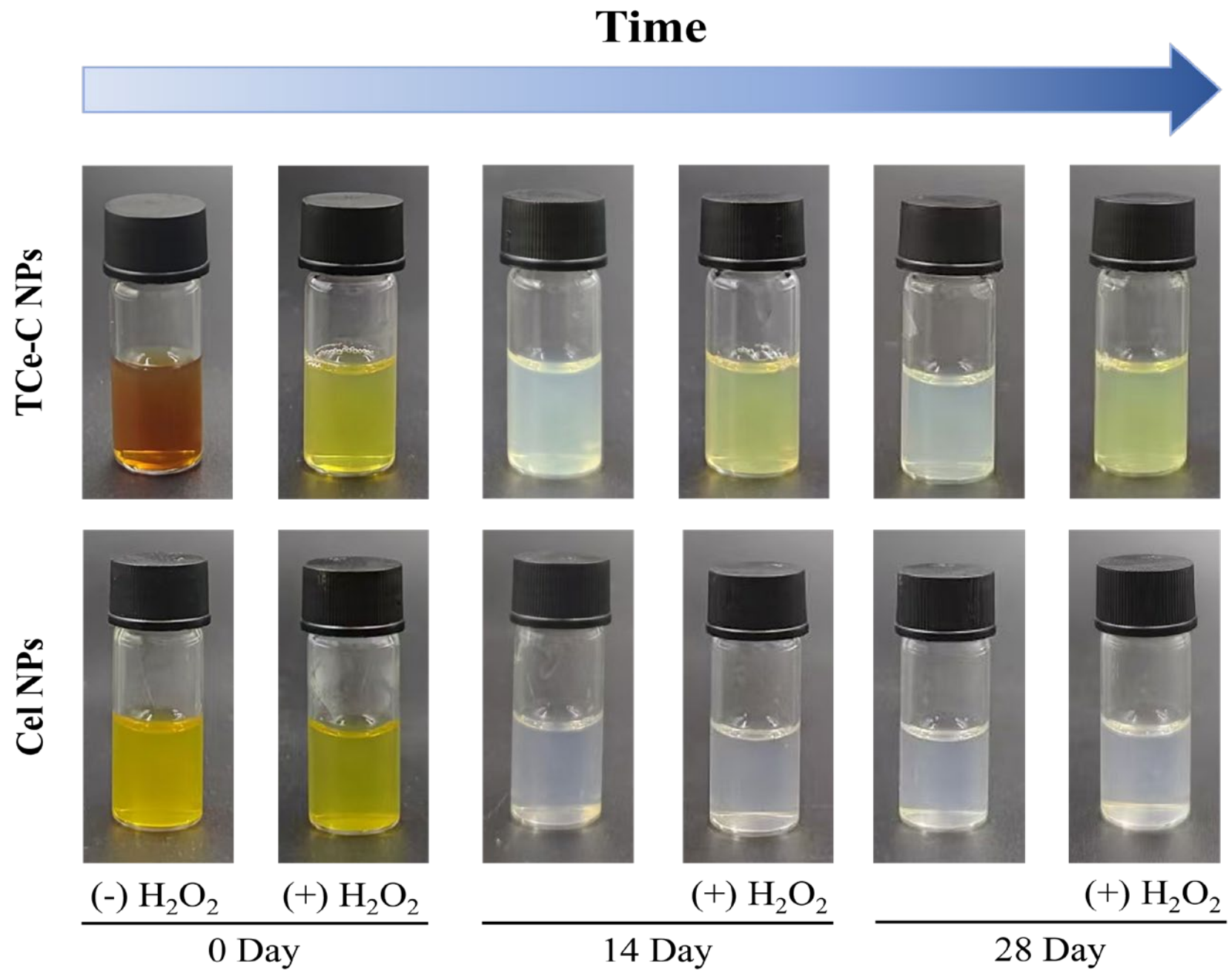

Figure S6. Regenerative ROS scavenging by TCe-C NPs. Photographic evidence of TCe-C NPs color transition following treatment with 1 mM H_2O_2.

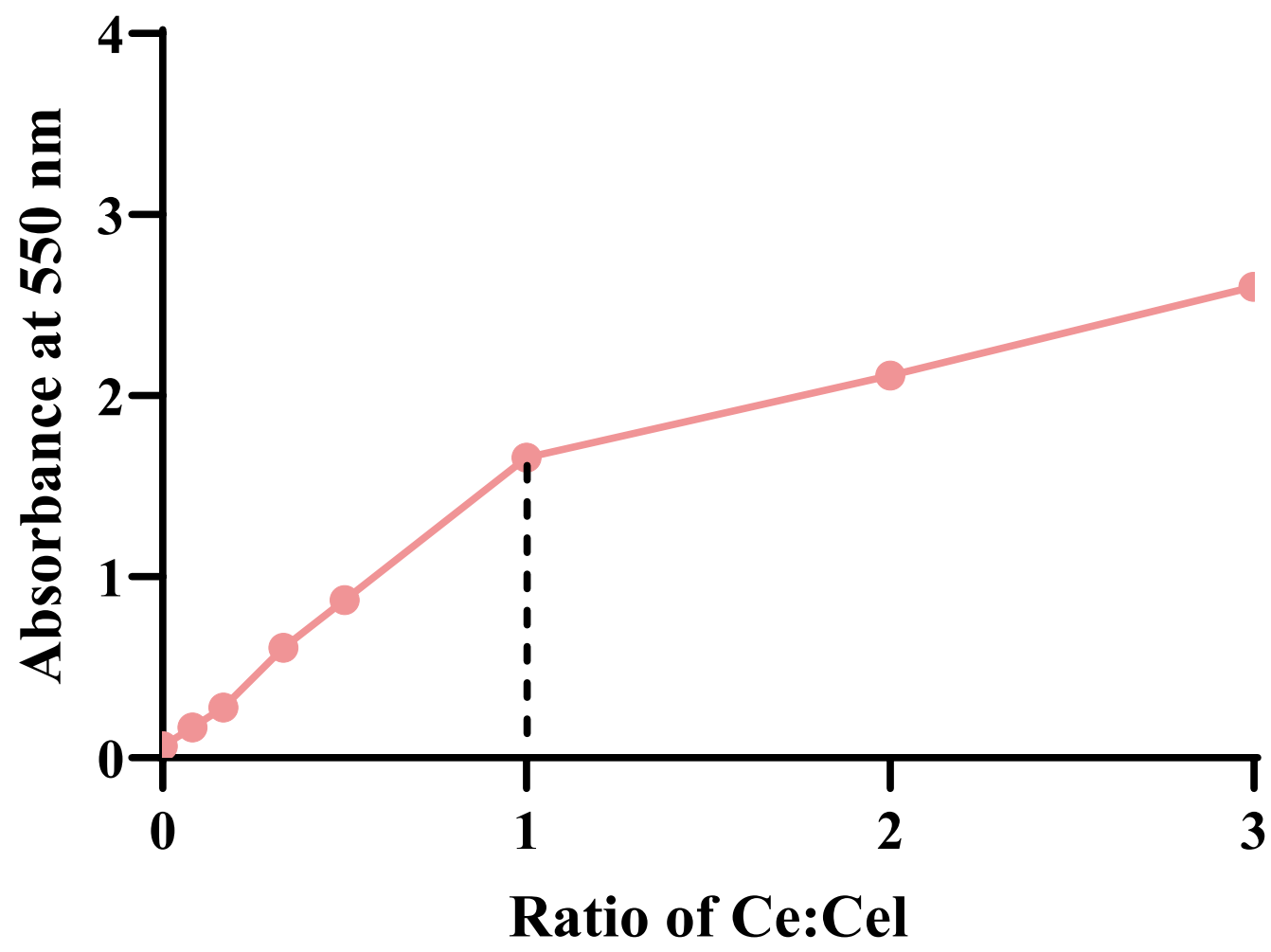

Figure S7. Absorbance at 550 nm (A_{550}) versus Ce:Cel molar ratio. A_{550} increases linearly with the ratio of Ce: Cel from 1:12 to 1:1 with significantly reduced slope at ratios ≥1:1, indicating complex research saturation.

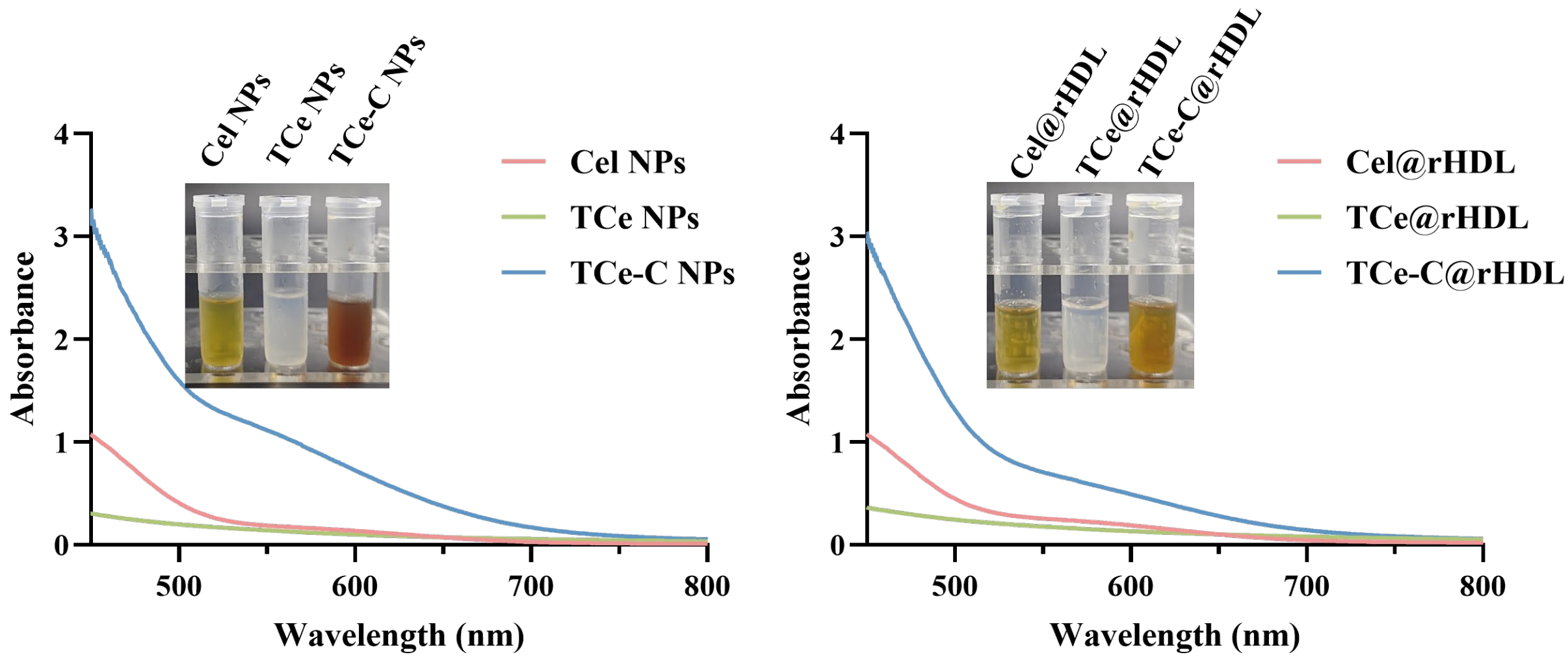

Figure S8. UV-vis absorption spectra of Cel, TCeNZ, and TCeNZ-Cel-loaded formulations. Insets: Photographs showing the color changes observed in lipid nanospheres (left) and rHDL nanoparticles (right).

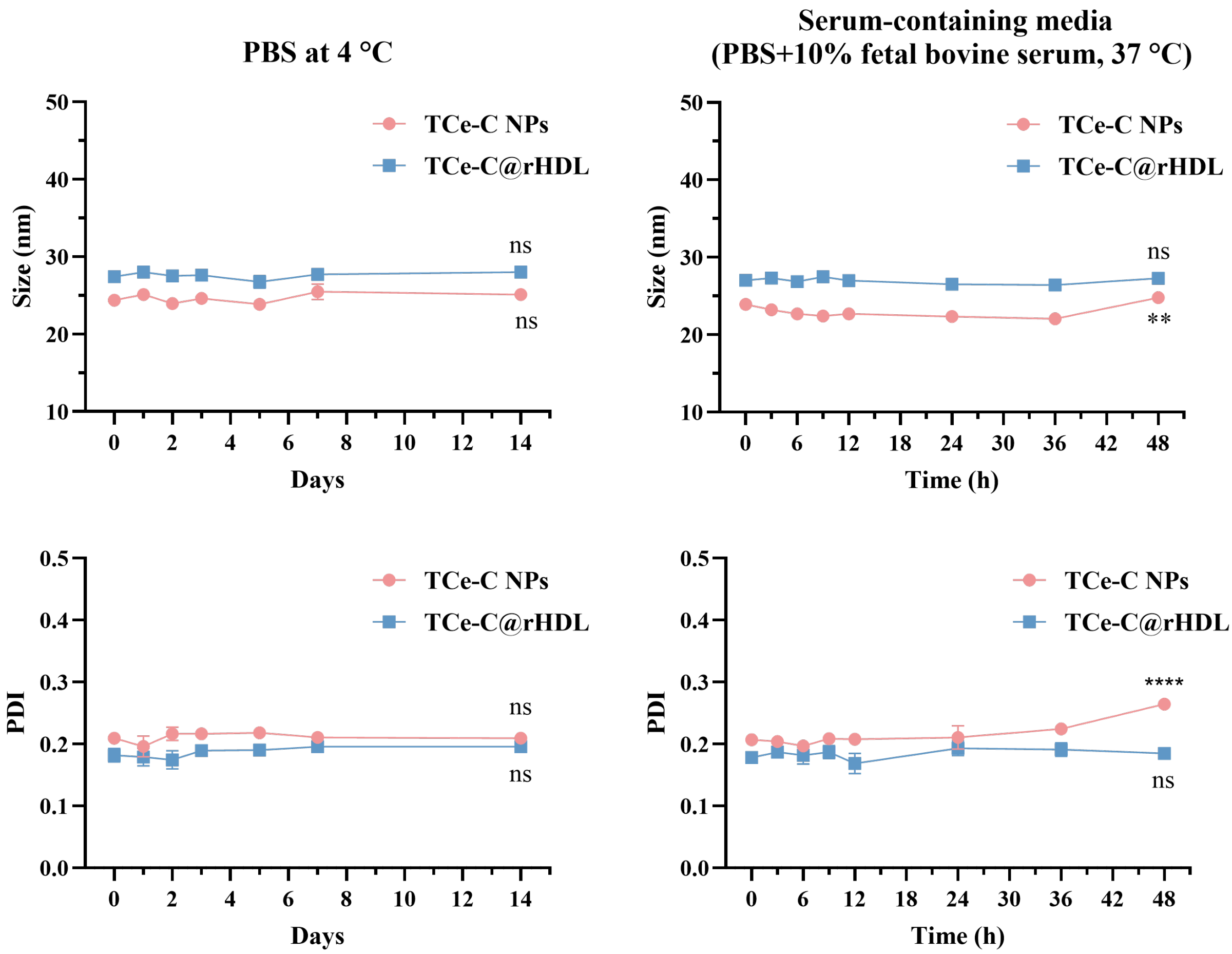

Figure S9. Time-dependent stability of the TCe-C NPs and TCe-C@rHDL in PBS or serum-containing media (i.e., PBS+10% fetal bovine serum, 37°C) with their sizes and PDI (mean ± SD, n=3).

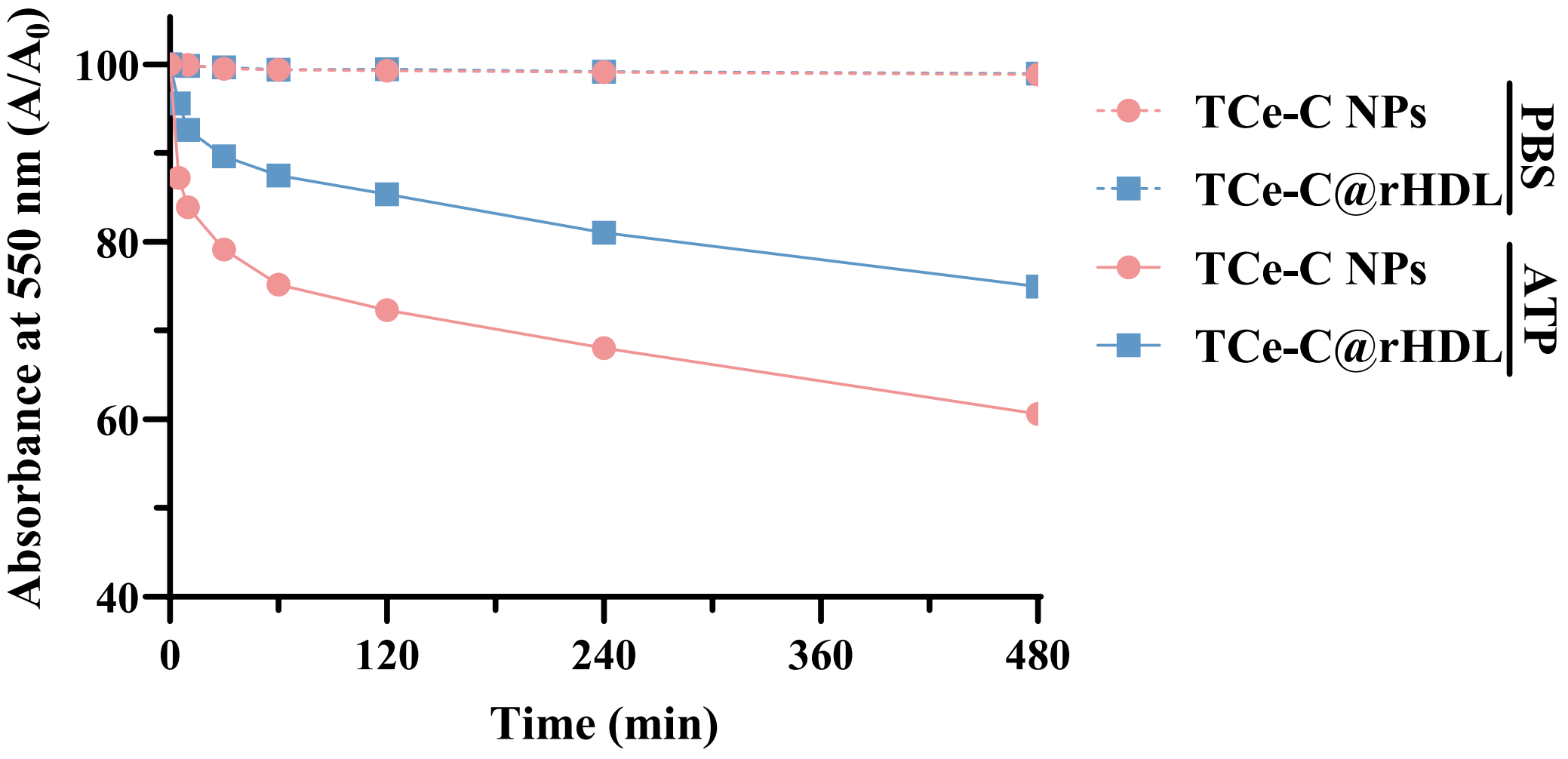

Figure S10. Stability of the coordination bonds in TCe-C NPs and TCe-C@rHDL at 0.5 mM ATP or PBS buffer (n=3).

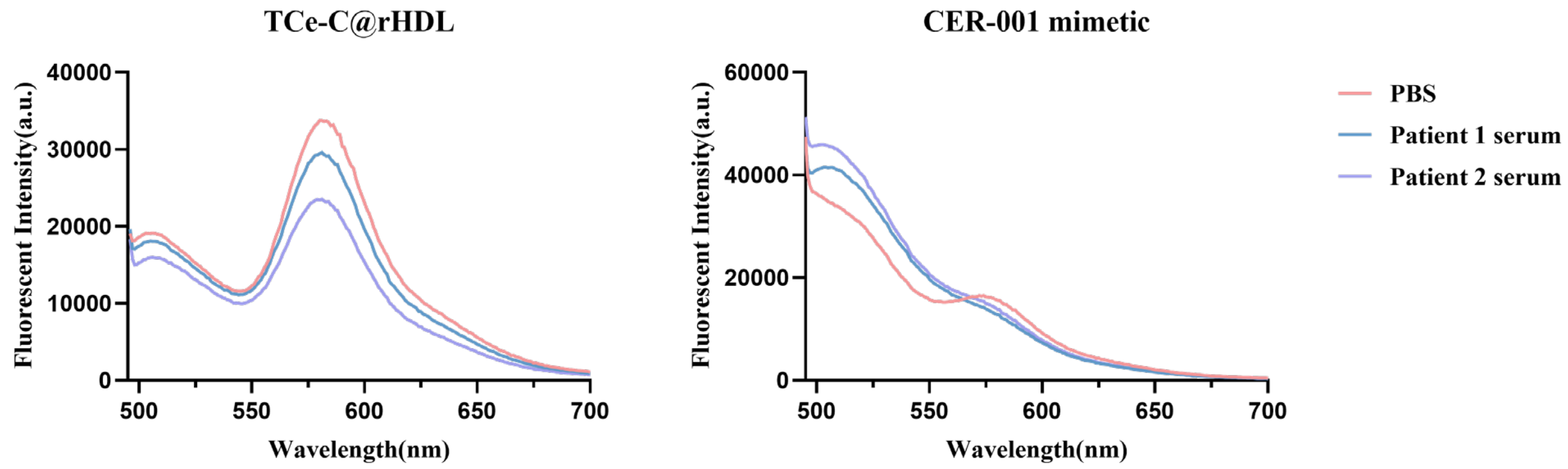

Figure S11. Fluorescence emission spectra of TCe-C@rHDL and CER-001 mimetic incubated with serum from Patient 1 and Patient 2.

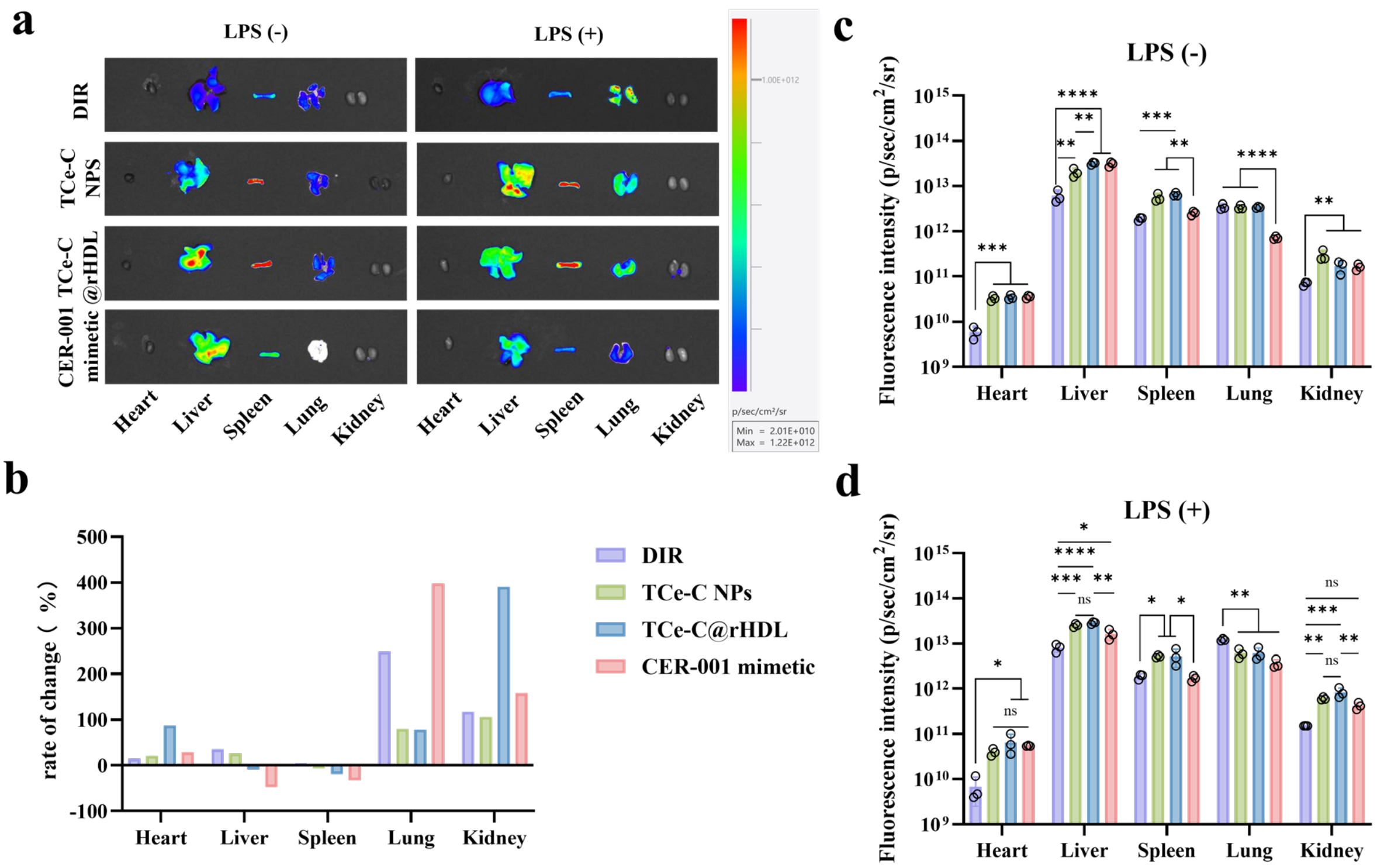

Figure S12. Biodistribution studies of different formulations. **a** Representative images of organs (heart, liver, spleen, lung, kidney) of different formulations (free DIR, TCe-C NPs, TCe-C@rHDL, CER-001 mimetic) in LPS (-) groups and LPS (+) groups. **b** Rate of change in fluorescence intensity of each formulation in LPS-treated mice compared with healthy mice. **c, d** Fluorescent quantitation of organs in LPS (-) groups and LPS (+) groups (mean ± SD, n = 3).

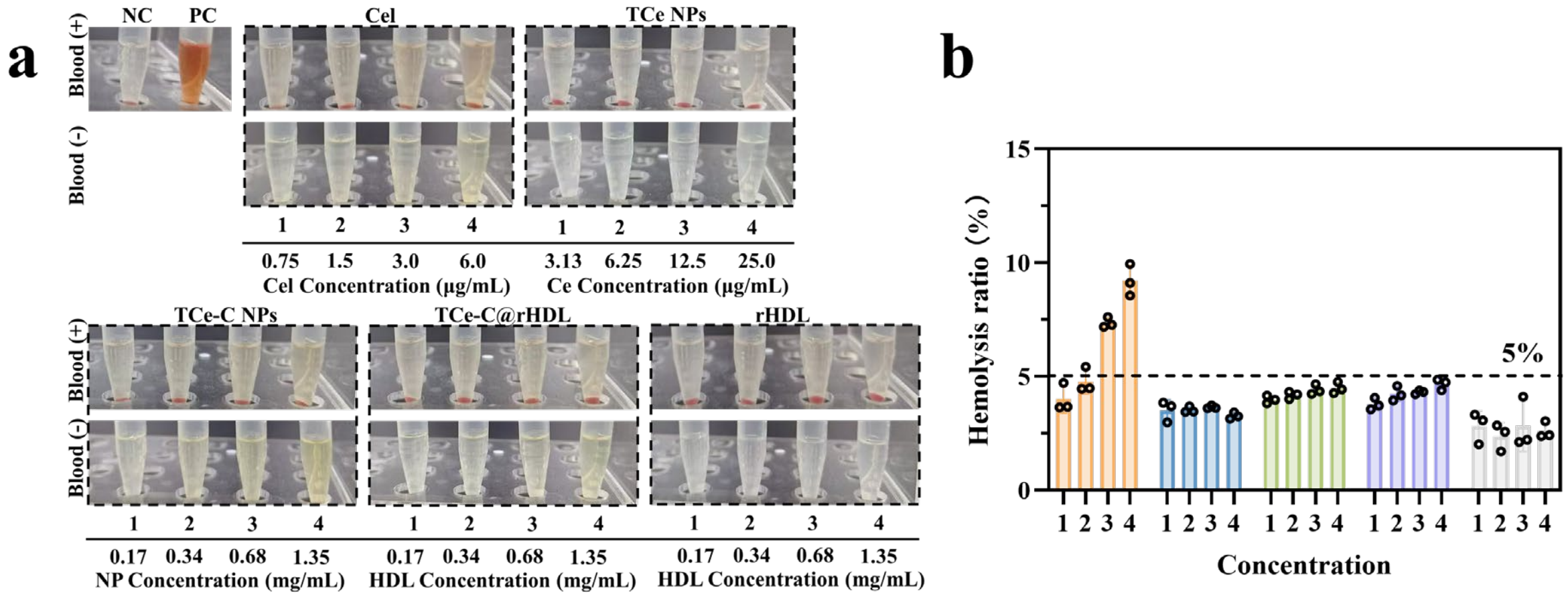

Figure S13. Hemolytic tests. (a) The effect of various formulations with different concentrations on red blood. (b) hemolysis ratio of various formulations (mean ± SD, n = 3).

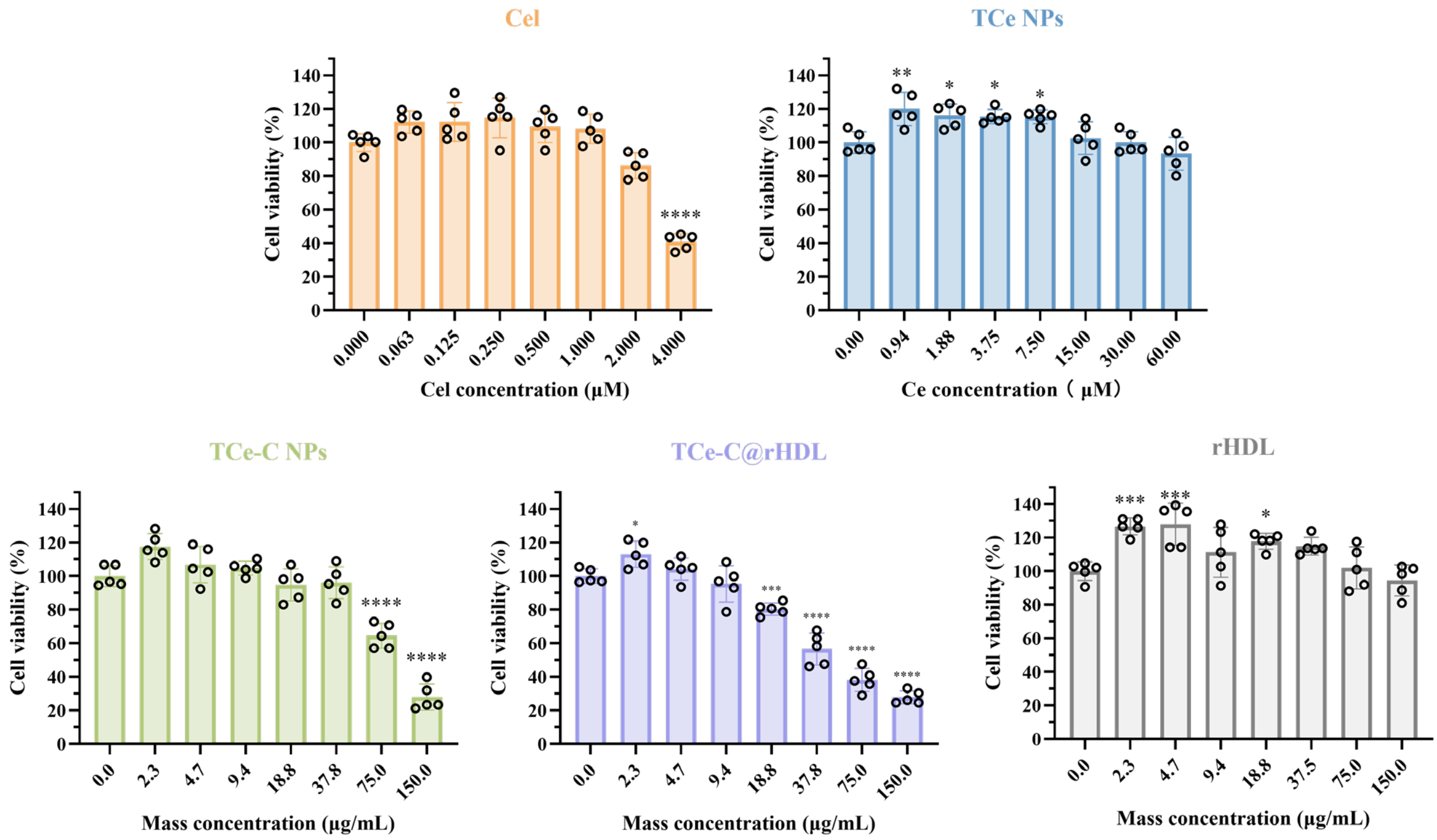

Figure S14. The cytotoxicity of different concentrations of formulations on RAW 264.7 cells after 24 h (mean ± SD, n = 5).

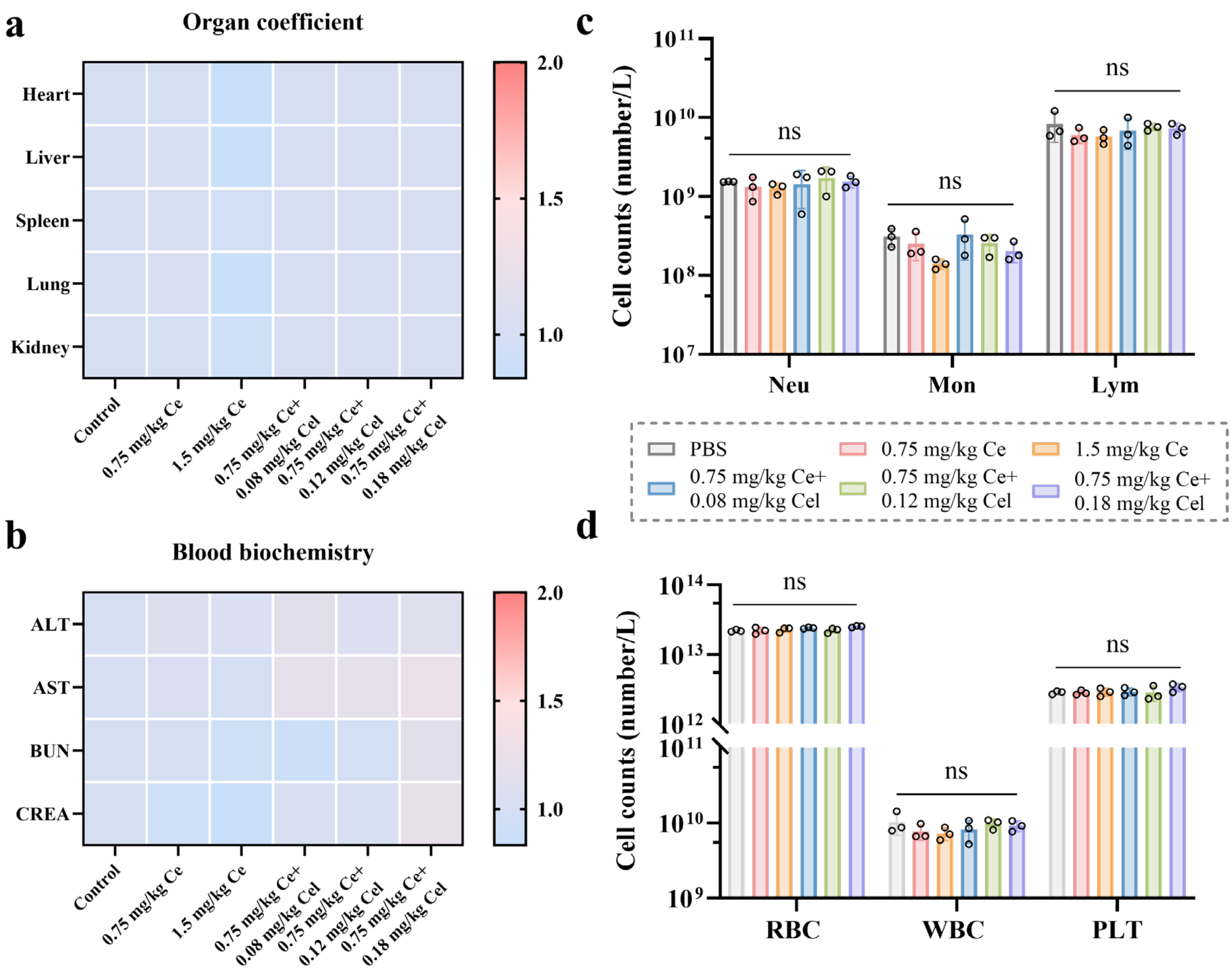

Figure S15. The safety tests of different doses of HDL formulations containing Ce or Ce + Cel. a) The heat map of organ coefficients and b) blood biochemistry of health mice at 72 h post injection (n = 3). All groups were normalized by Control group. Statistical differences compared to the data in PBS group were calculated via one-way ANOVA with Tukey's post hoc test. However, no significant difference was observed between any of the groups and the control group ($P > 0.05$). c) The blood cell counts (mean ± SD, n = 3). RBC is the red blood cell count; WBC is the white blood cell count; PLT is the platelet count. d)The leukocyte classification counts (mean ± SD, n = 3). Neu is the neutrophil count; Mon is the monocyte count; Lym is the lymphocyte count.

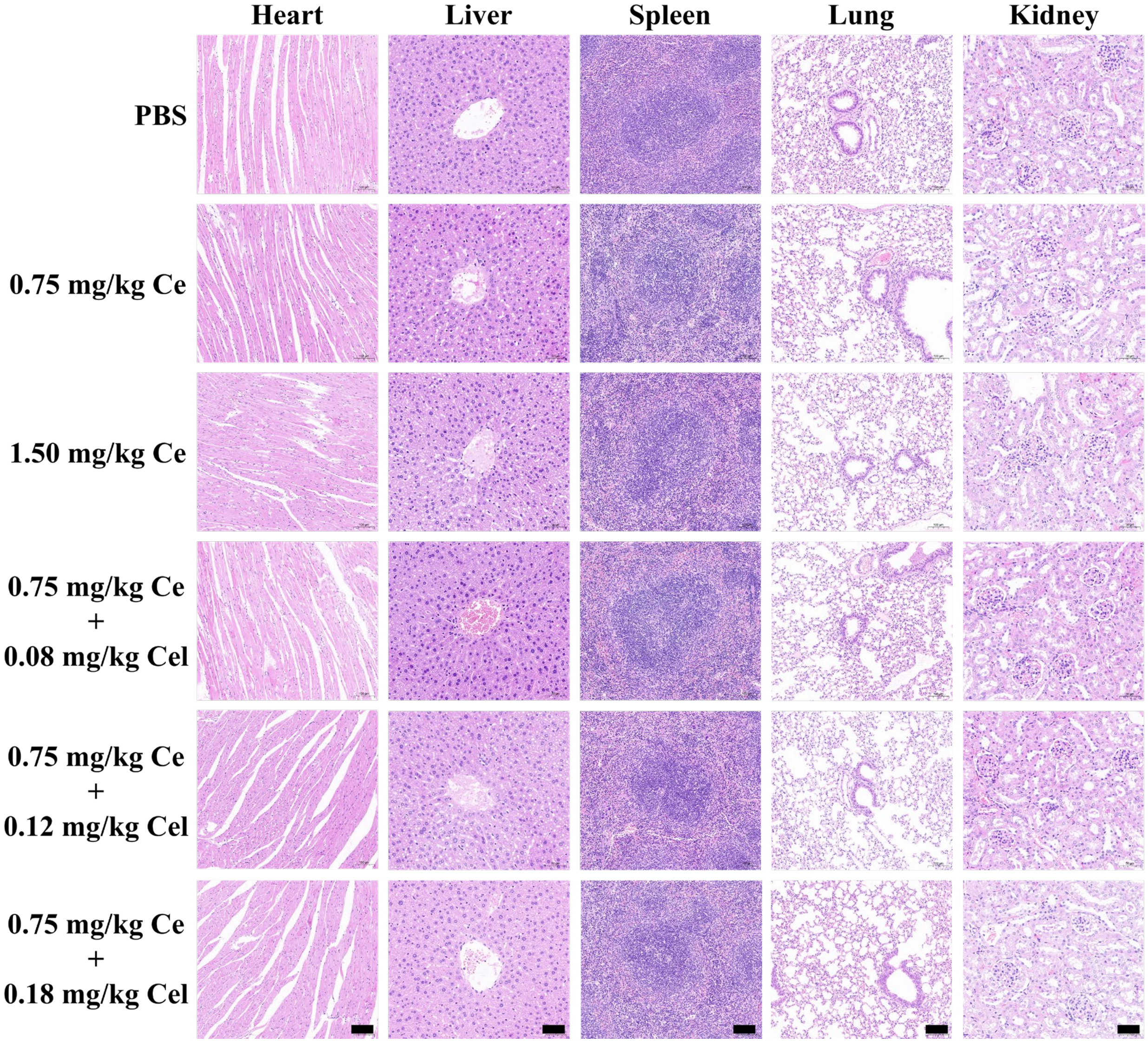

Figure S16. H&E staining in the safety tests. The scale bars in hearts, spleens, and lungs ware 100 μm. The scale bars in livers and kidneys are 50 μm.

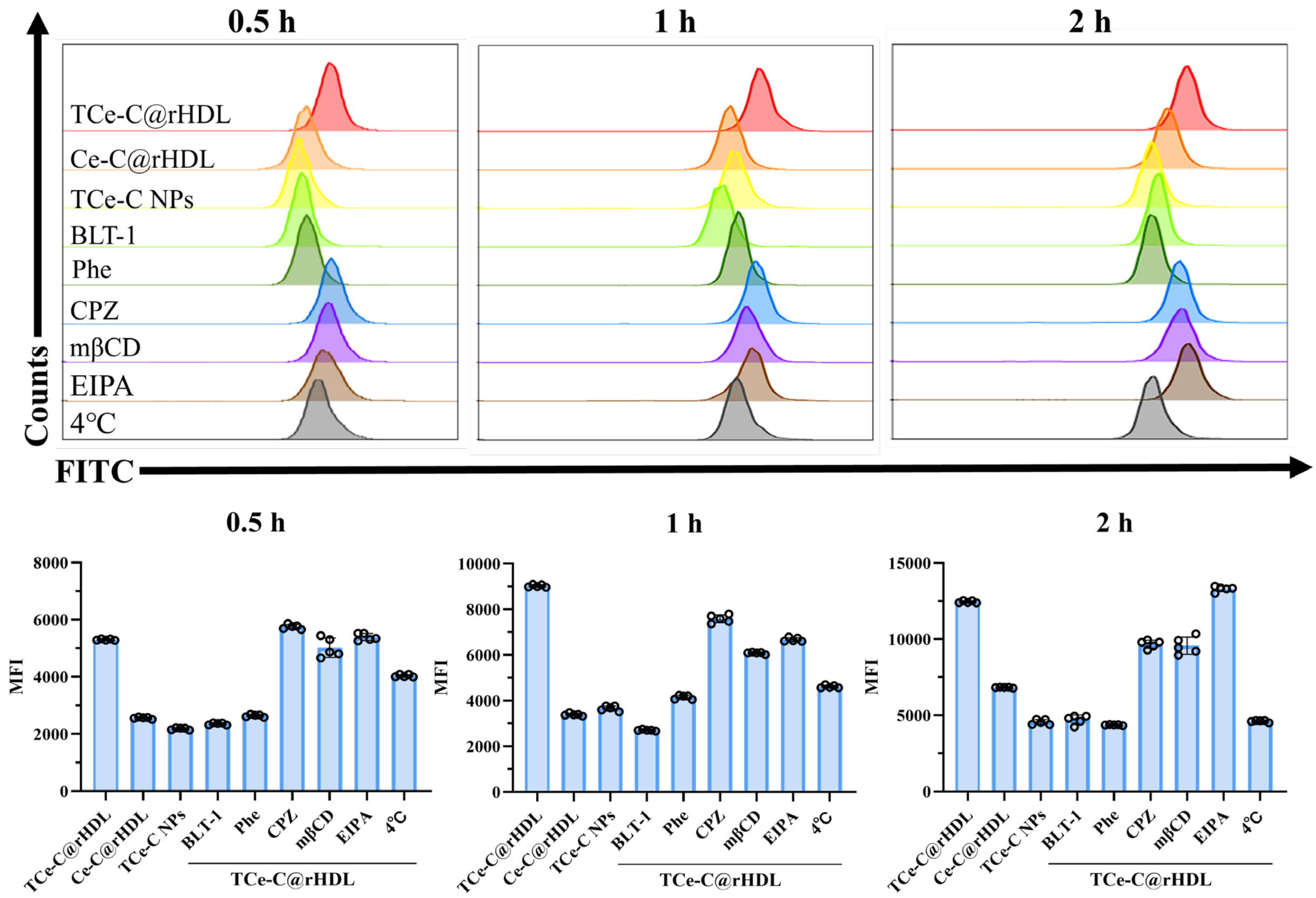

Figure S17. Flow cytometry detection of RAW264.7 uptake of TCe-C@rHDL, Ce-C@rHDL, TCe-C NPs or after different inhibitors treatments at 0.5, 1, and 2 h (mean ± SD, n = 5).

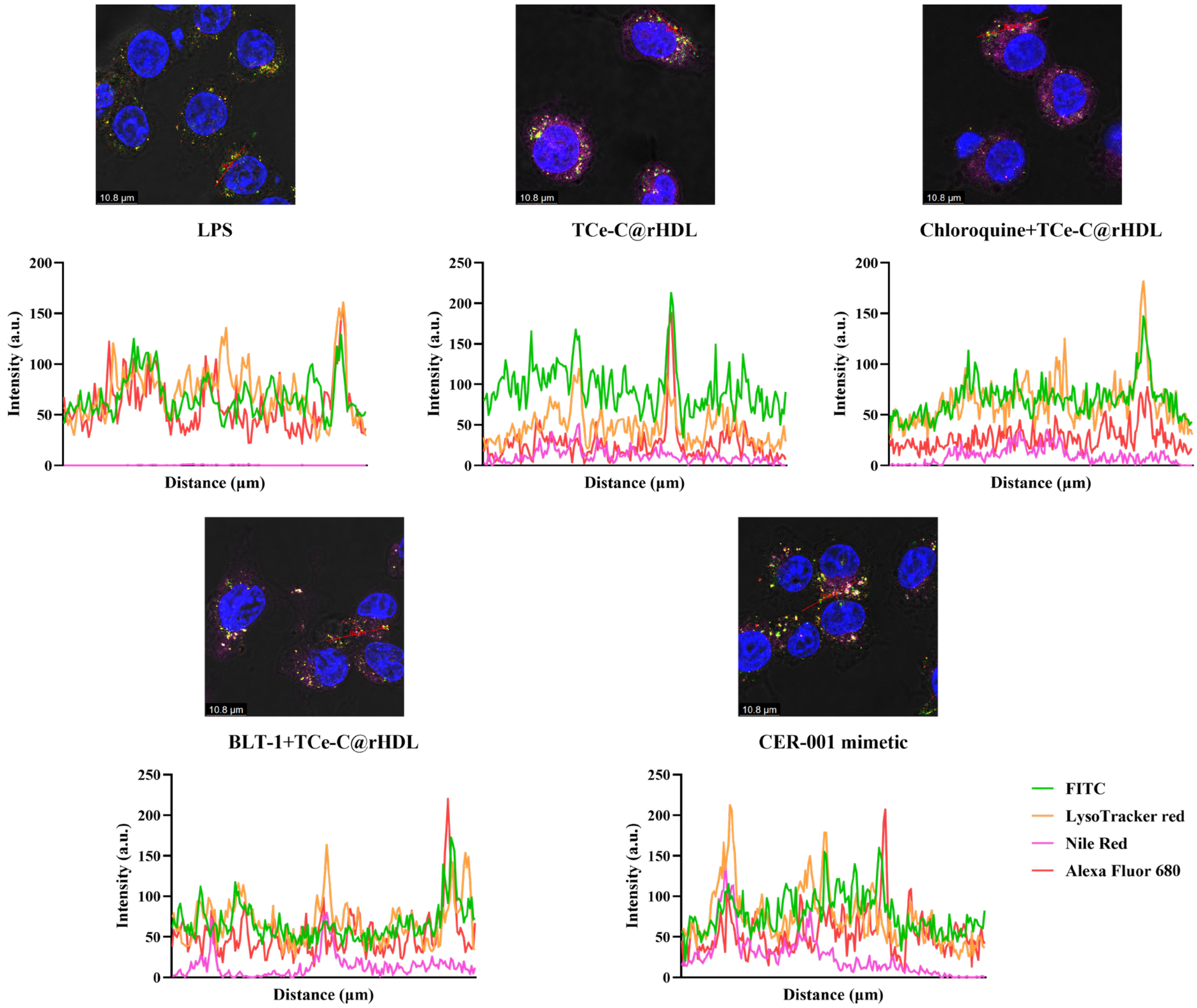

Figure S18. Multichannel fluorescence imaging and colocalization analysis. Green: FITC-LPS; Magenta: Nile Red-labeled Cel; Yellow: LysoTracker Red-labeled lysosomes; Red: Alexa Fluor 680-labeled TLR4. Scale bars: 10.8 μm.

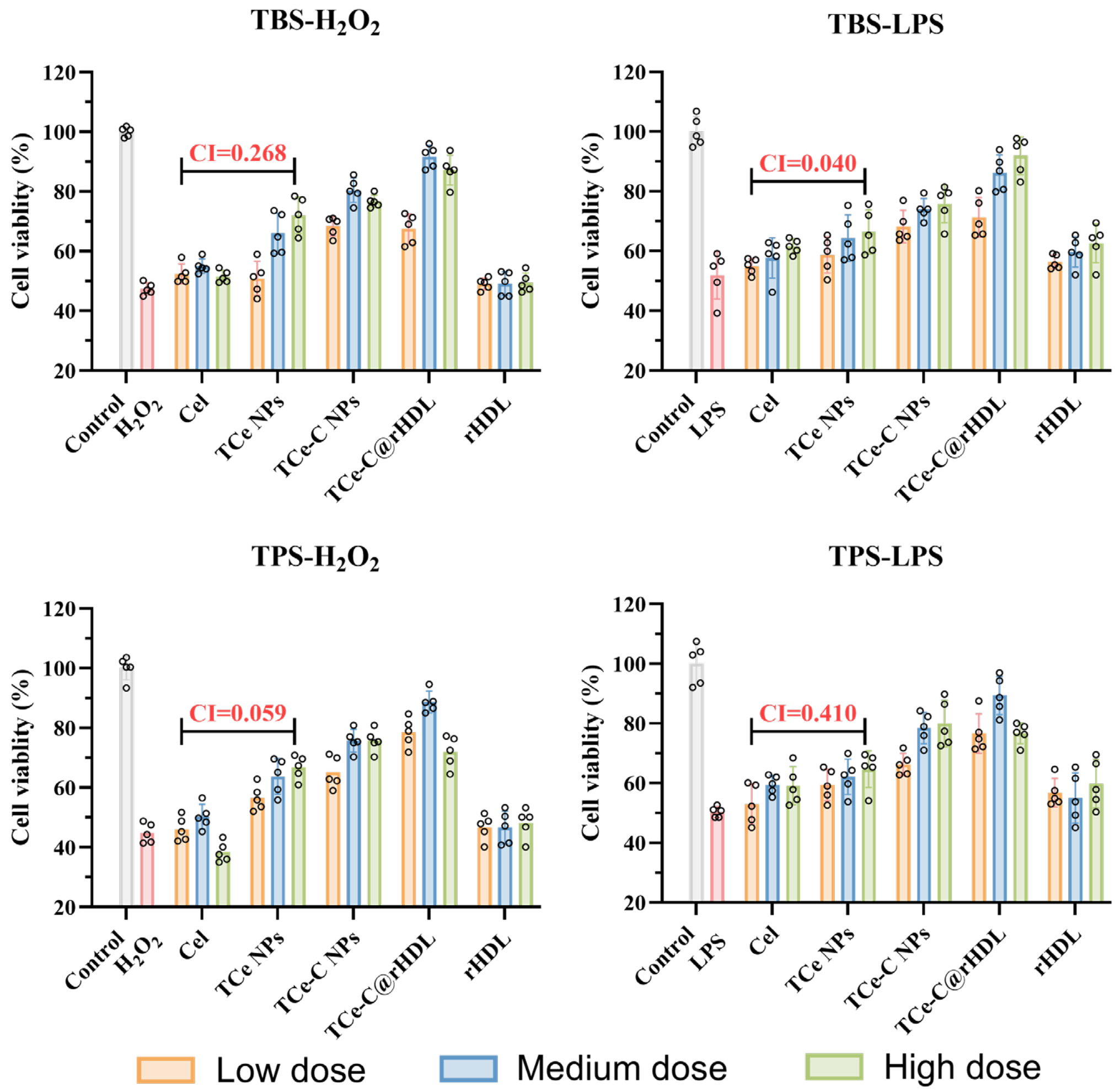

Figure S19. Cell viabilities of H_2O_2 or LPS-stimulated RAW264.7 cells treated with different interventions including treatment before stimulation (TBS) and treatment post stimulation (TPS). The concentration of H_2O_2 and LPS was 800 μM and 1.0 μg/mL respectively; The low, medium, and high doses corresponding to 0.63 μM Cel/0.94 μM Ce, 0.125 μM Cel/1.88 μM Ce, and 0.25 μM Cel/3.75 μM Ce respectively. The synergistic interaction between Cel and TCeNZ was quantitatively evaluated using the combination index (CI) method based on the median-effect principle of Chou and Talalay. (mean ± SD, n = 5).

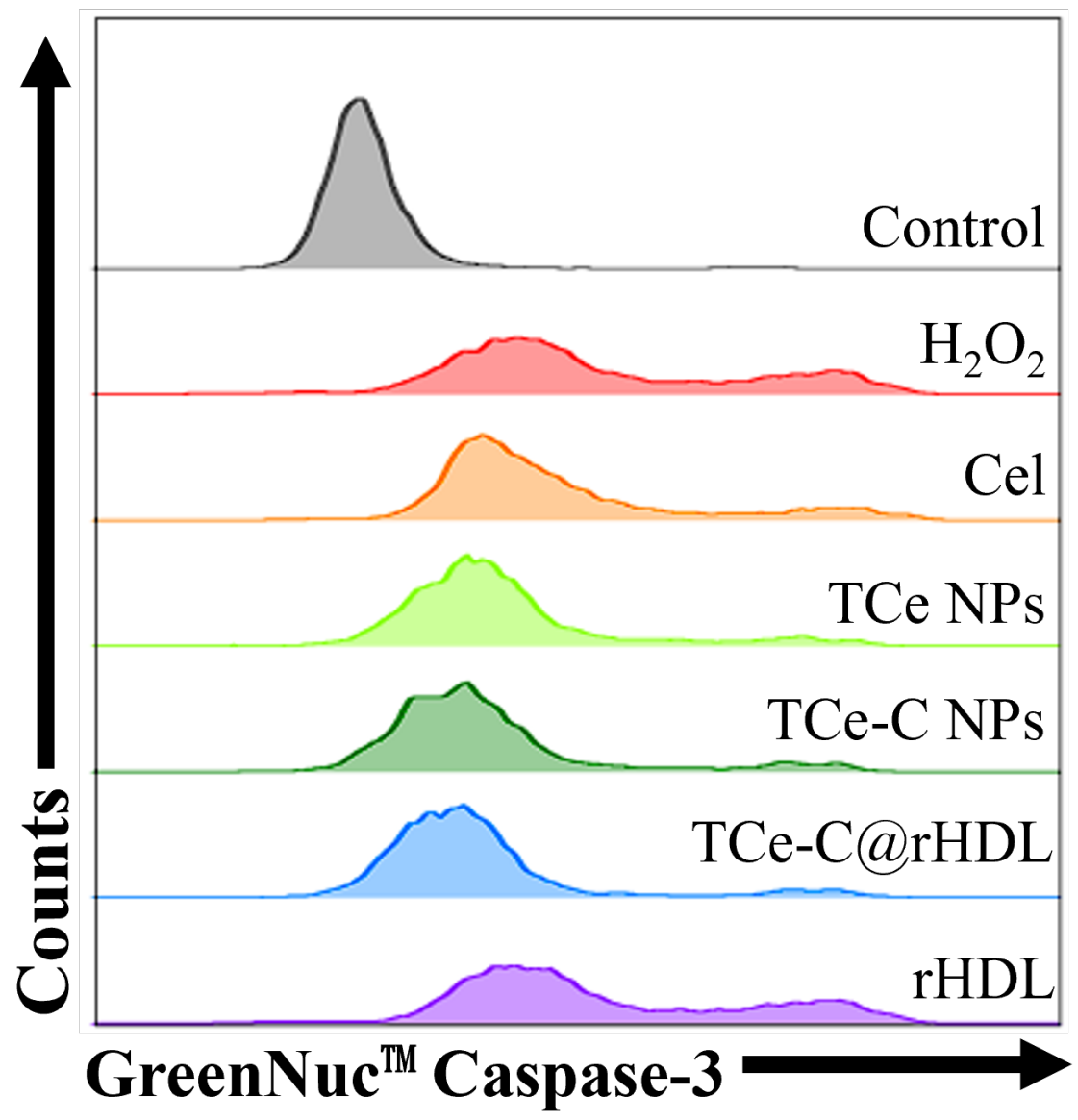

Figure S20. Flow cytometry analysis of intracellular caspase-3 levels in H_2O_2-stimulated RAW264.7 cells.

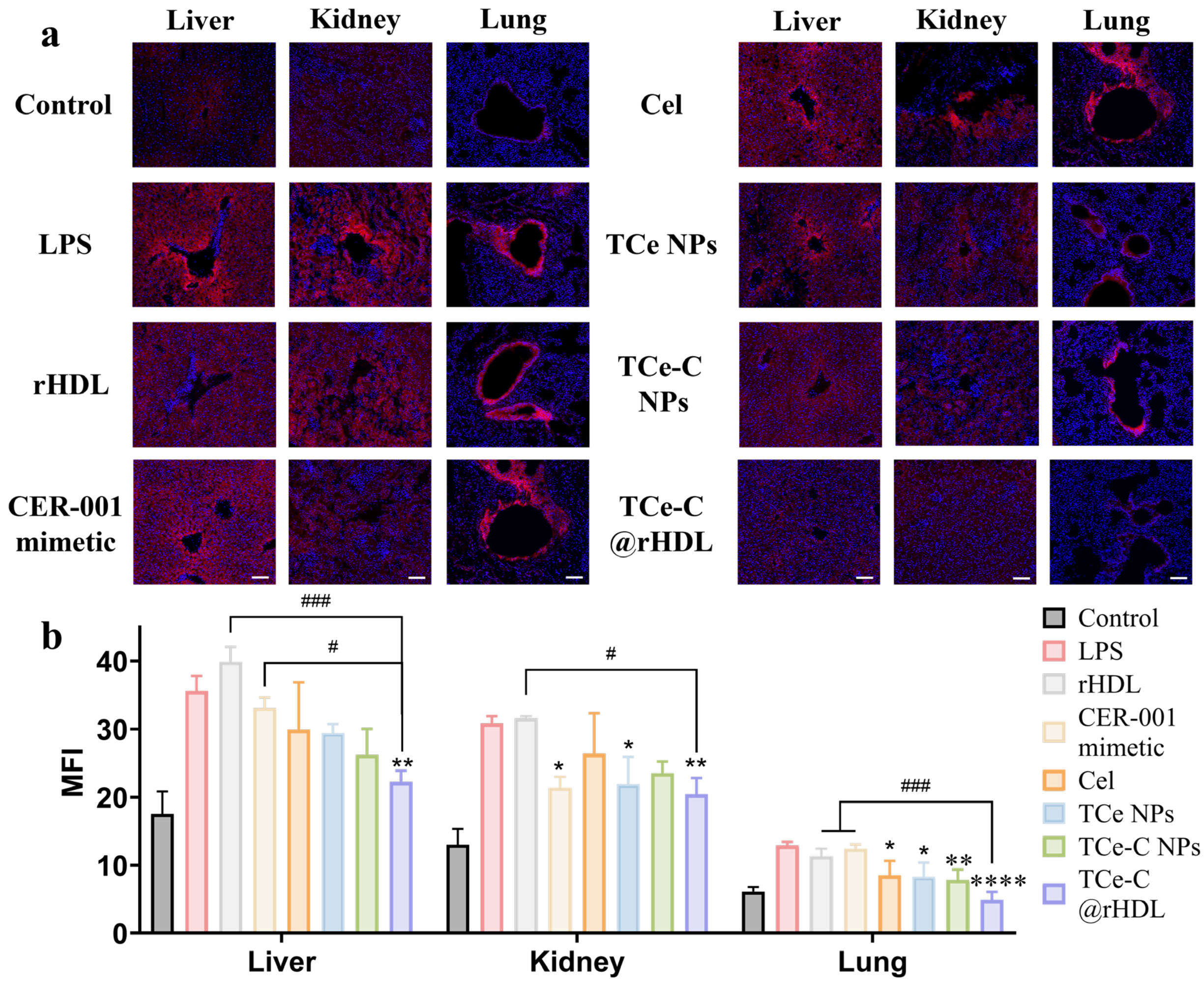

Figure S21. Hepatic superoxide detection by DHE staining in LPS-challenged mice after 24-hour treatment with experimental formulations. a) Representative fluorescence images of DHE-stained liver, kidney, and lung sections. scale bar: 100 μm. Red fluorescence intensity correlates with superoxide levels. b) Quantitative analysis of DHE fluorescence intensity (mean ± SD, n = 3). Symbols above the bars denote significant differences compared to the LPS group: ns (not significant), *p<0.05, **p<0.01, ***p<0.001, ****p<0.0001. Significance markers denoted by hashes (#) above the horizontal lines indicate comparisons with the TCe-C@rHDL group: #p<0.05, ##p<0.01, ###p<0.001, ####p<0.0001.

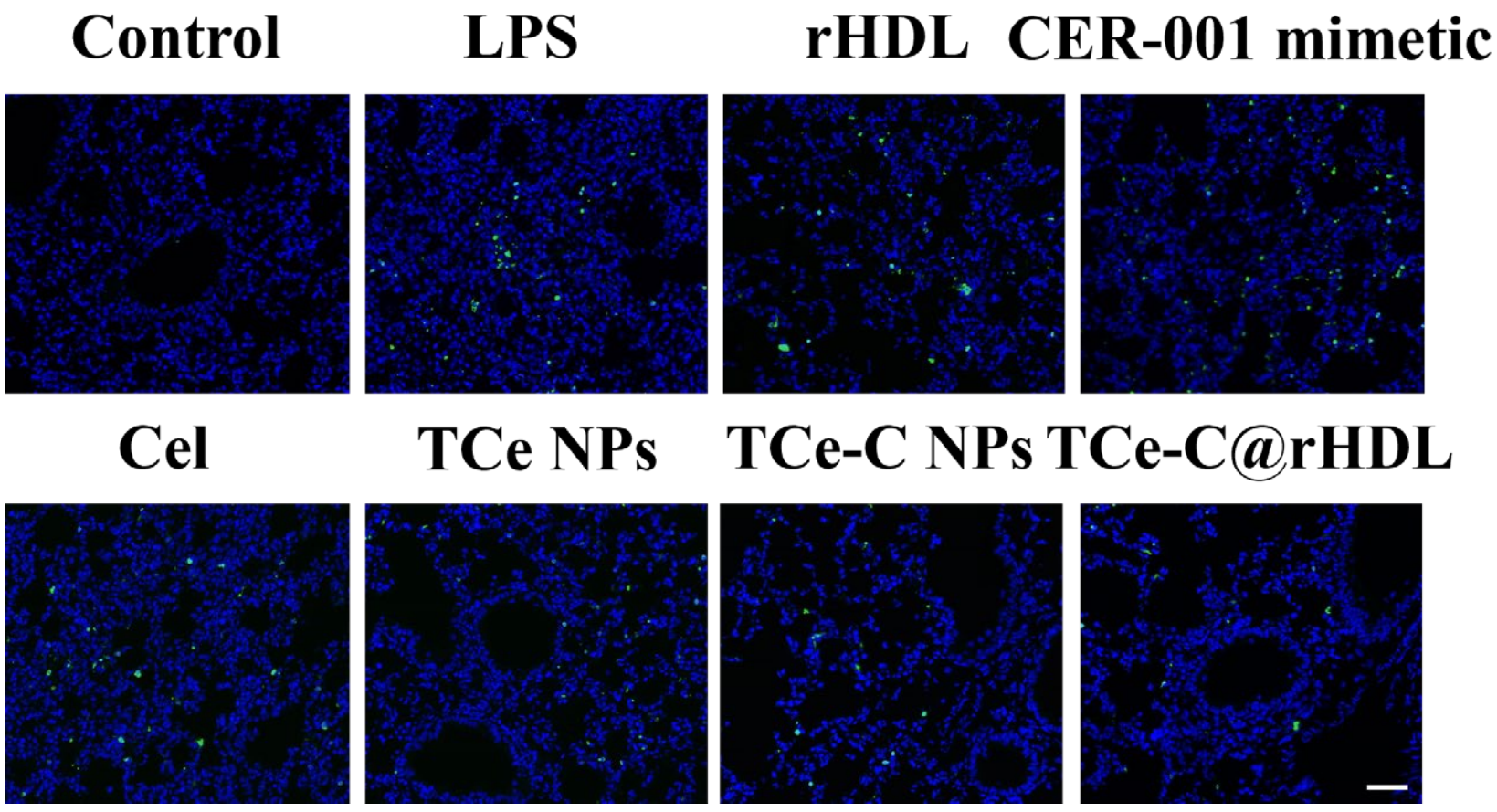

Figure S22. Representative fluorescence images of TUNEL- stained lung sections in LPS-challenged mice after 24-hour treatment with experimental formulations. Green fluorescence intensity correlates with apoptotic levels. Scale bars: 100 μm.

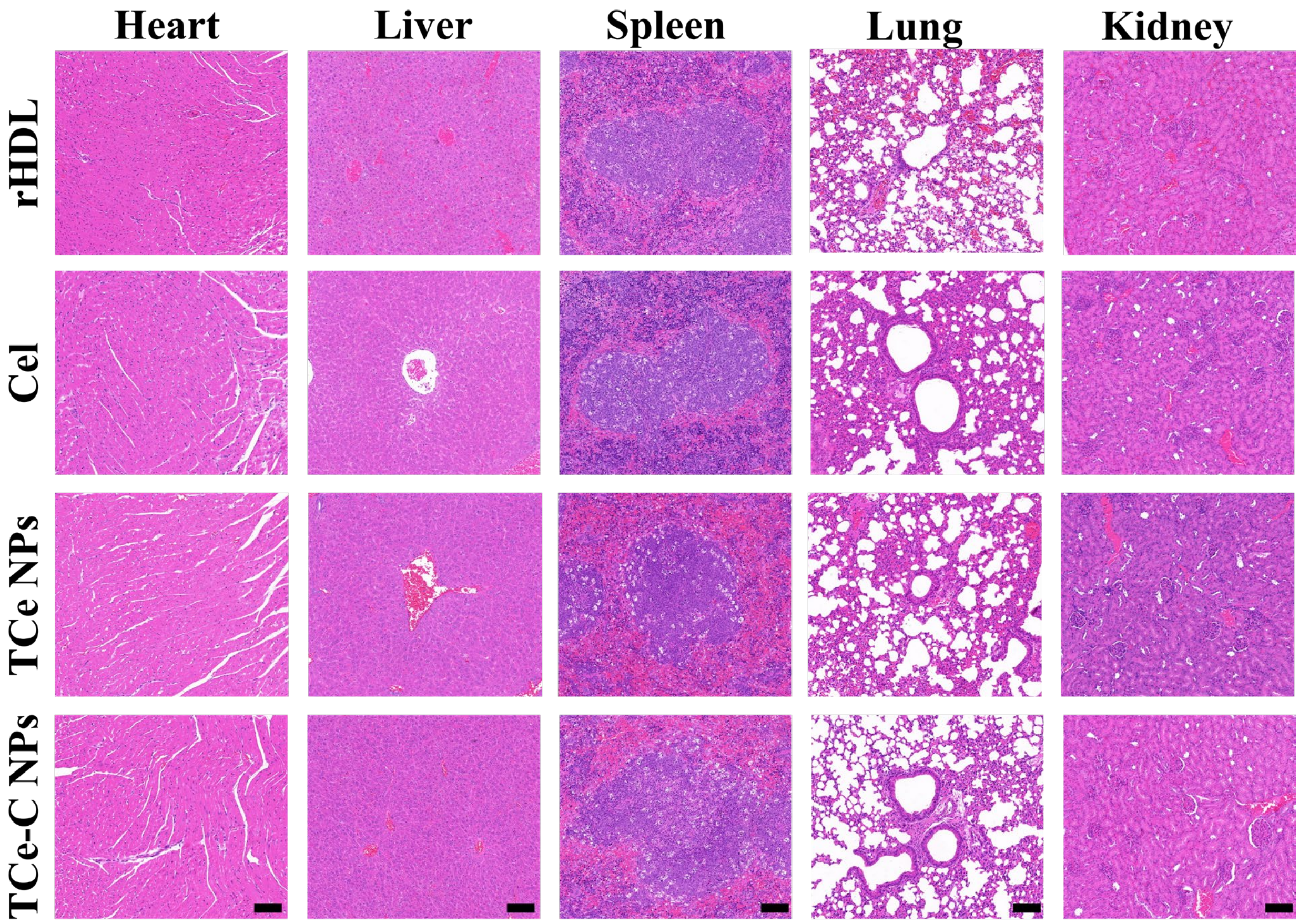

Figure S23. Representative images of H&E staining in LPS-challenged mice. Scale bars: 100 μm.

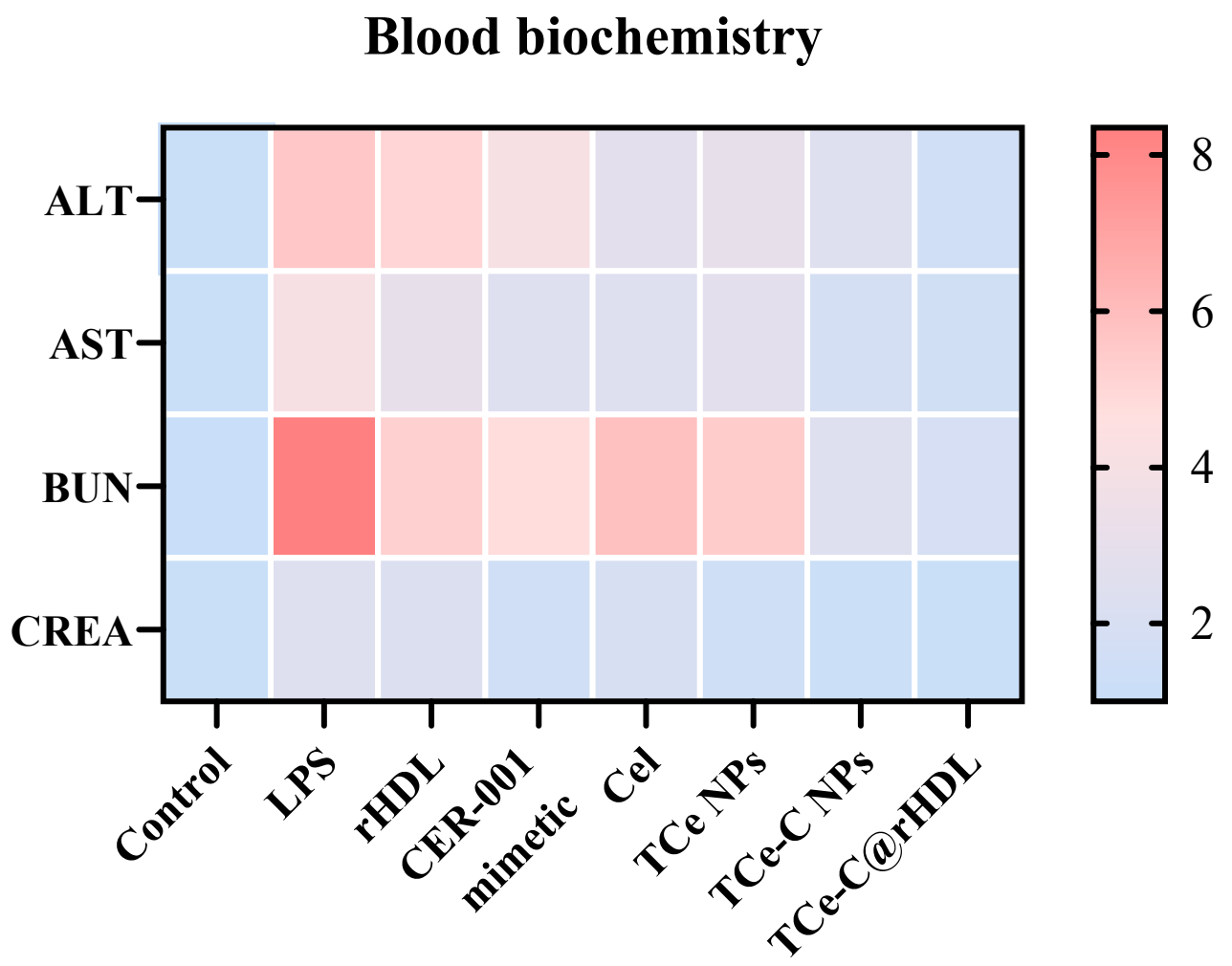

Figure S24. The heat map of blood biochemistry in LPS-challenged mice. All groups were normalized by Control group.

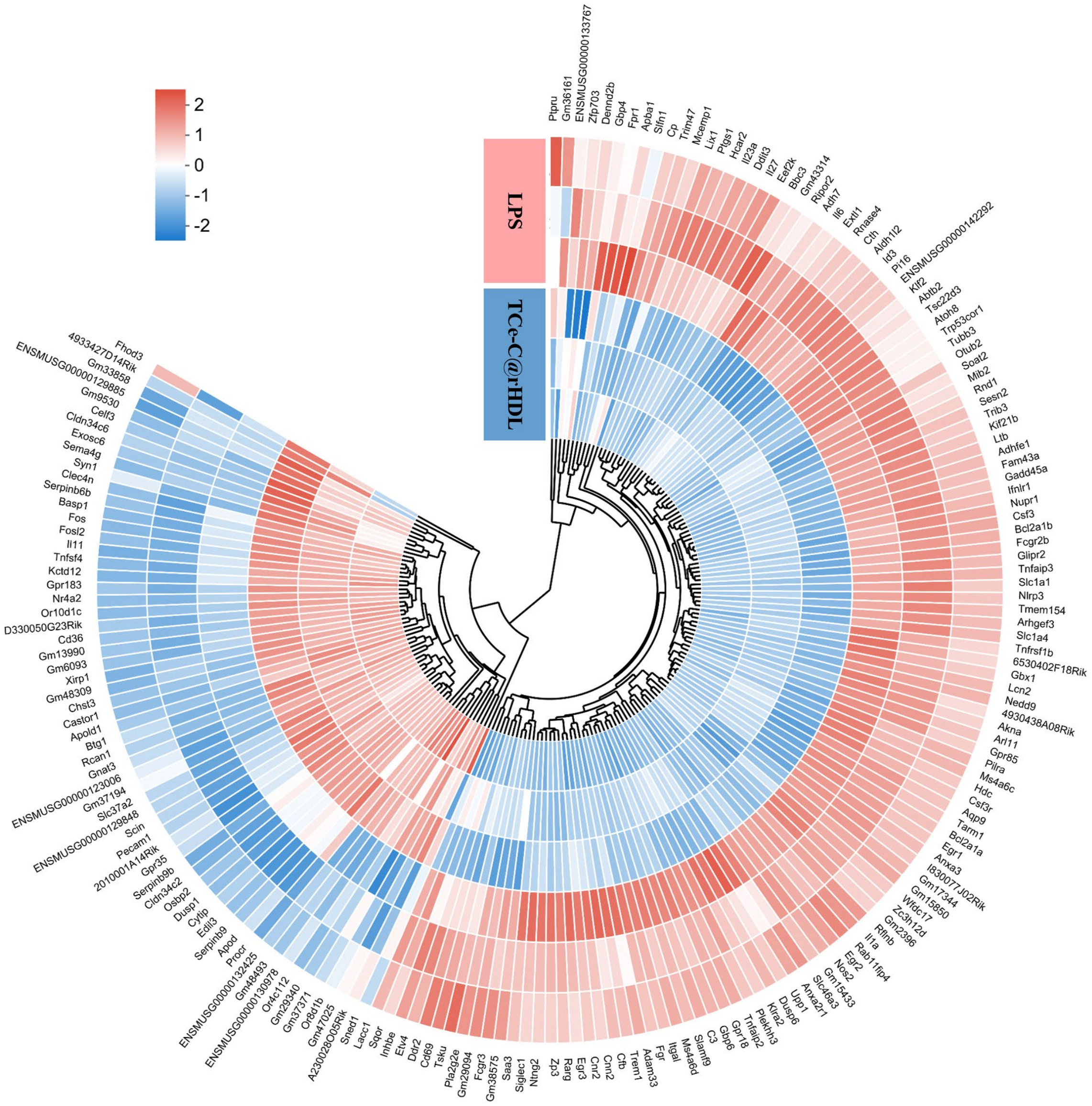

Figure S25. Heatmap of gene expressions of TCe-C@rHDL treatment in LPS-stimulated RAW264.7.

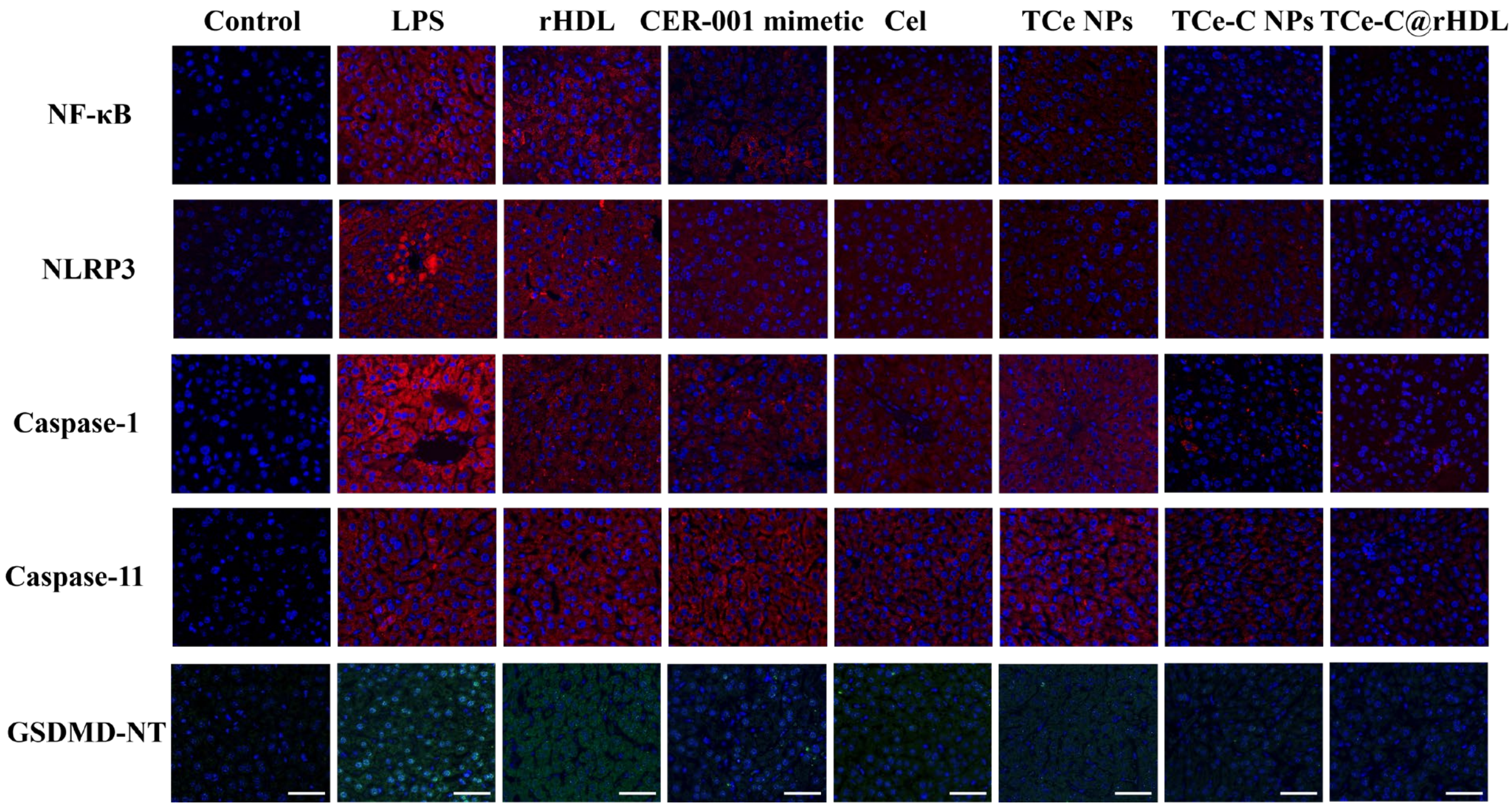

Figure S26. Representative fluorescence images of hepatic NF-κB, NLRP3, caspase-1, caspase-11, and GSDMD-NT expression in LPS-challenged mice. Scale bars: 50 μm.

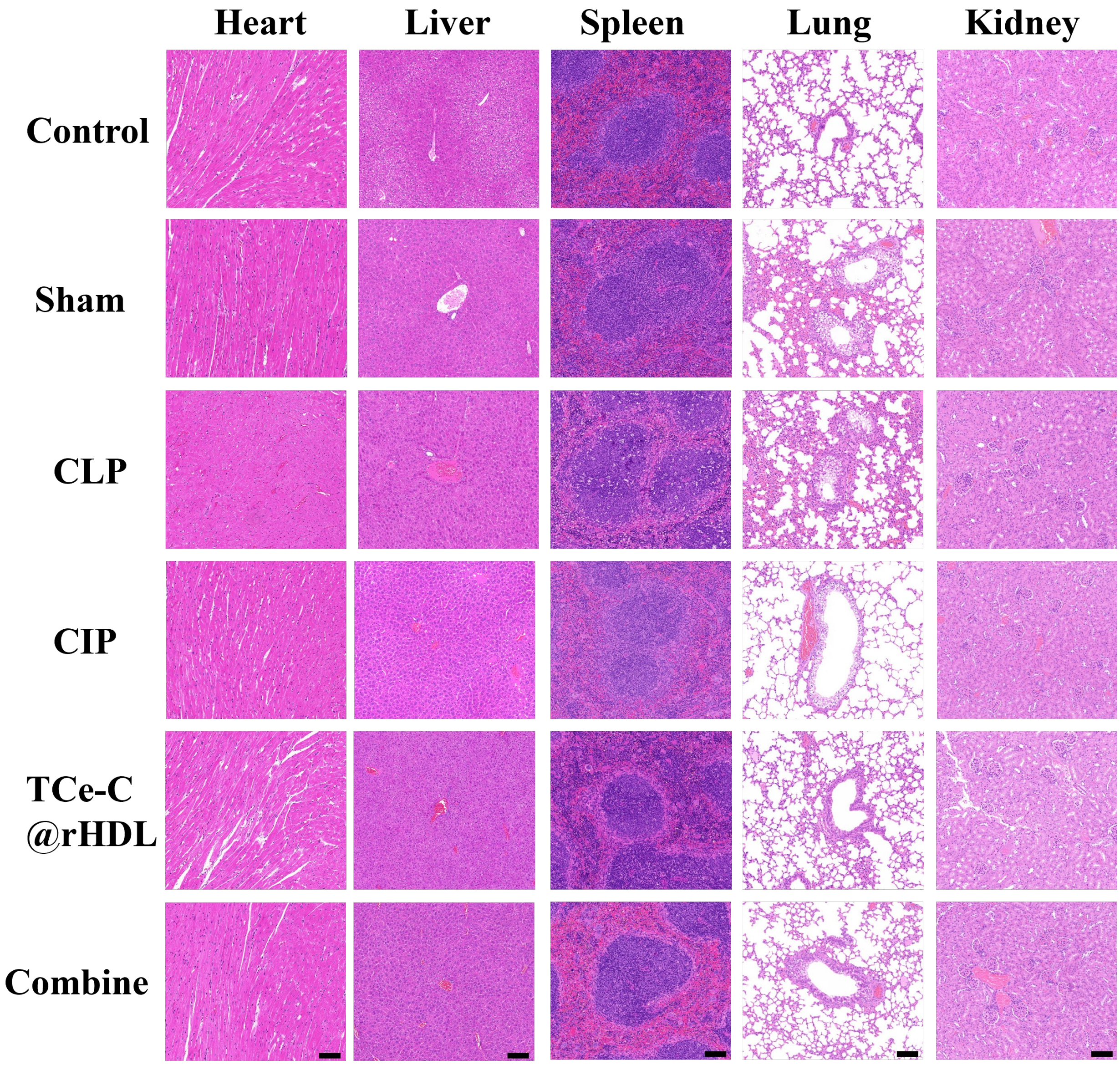

Figure S27. Representative images of H&E staining in CLP-induced septic mice. Scale bars: 100 μm.

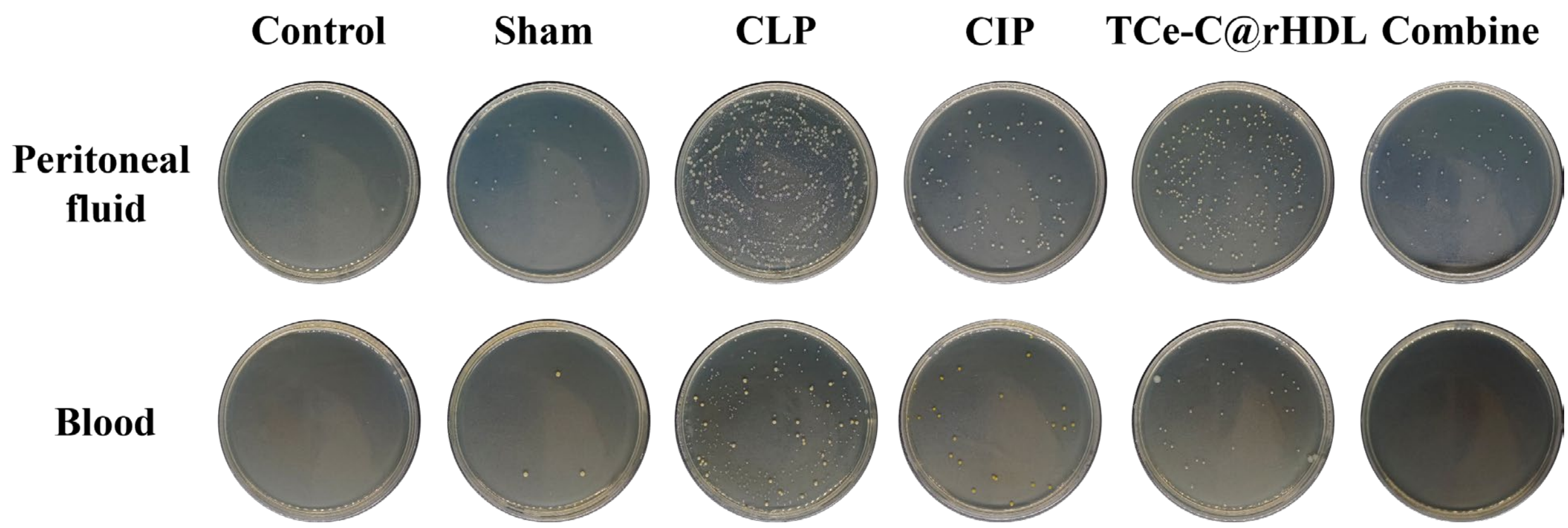

Figure S28. Representative bacterial colony images from cultures of peritoneal lavage fluid and whole blood samples in septic mice induced by CLP.

Table S1. Hydrated particle size and Zeta potential of different formulations (mean ± SD, n = 3).

Formulations	Size（nm）	Zeta potential（mv）
nHDL	21.07 ± 4.12	-9.76 ± 1.52
CER-001 mimetic	24.37 ± 3.31	-17.63 ± 0.17
CSL-112 mimetic	30.48 ± 4.04	-5.99 ± 1.23
rHDL	21.13 ± 2.03	-10.58 ± 1.00
TCe@rHDL	25.06 ± 2.41	+9.99 ± 1.12
TCe NPs	23.29 ± 4.35	+10.63 ± 2.15
Ce@rHDL	24.42 ± 3.78	-25.59 ± 3.23
TCe-C@rHDL	28.05 ± 3.20	+9.64 ± 2.05

Table S2. Physicochemical properties of TCe-C NPs with different Ce/Cel feeding ratios (mean ± SD, n = 3).

Ce: Cel	Size (nm)	PDI	Zeta potential (mV)	Concentration of TCeNZ （μg/mL）	Concentration of Cel （μg/mL）
15:1	84.94 ± 2.33	0.364 ± 0.001	+3.5 ± 1.5	133.4 ± 7.8	18.1 ± 2.3
9:1	24.82 ± 0.79	0.187 ± 0.009	+10.3 ± 1.9	282.0 ± 5.6	90.1± 4.2
6:1	46.42 ± 1.76	0.212 ± 0.002	+5.4 ± 2.8	231.8 ± 10.4	91.5 ± 7.3
3:1	53.52 ± 1.48	0.215 ± 0.006	-1.3 ± 2.2	146.5 ± 6.7	94.9 ± 5.1
1:1	64.93 ± 1.89	0.257 ± 0.005	-7.4 ± 1.7	67.4 ± 5.5	92.9 ± 3.7

Table S3. The EE and DL of TCe-C and TCe-C@rHDL (mean ± SD, n = 3)

**Formulation**	**EE_{TCeNZ}（%）**	**EE_{cel}（%）**	**DL_{TCeNZ}（%）**	**DL_{cel}（%）**
TCe-C NPs	99.94 ± 0.66	91.38 ± 1.75	8.29 ± 0.39	2.75 ± 0.20
TCe-C@rHDL	99.70 ± 0.76	89.43 ± 1.16	7.24 ± 0.34	1.66± 0.02

Table S4. The excitation and emission measurement of channels.

**Channel**	**E_x**	**E_m**
D	C6 (465 nm)	C6 (504 nm)
F	C6 (465 nm)	RBITC (576 nm)

Reference:

[1] W. Zhang, J. Wang, J. Jia, L. Chen, Z. Wu, J. Liu, *J Pharm Pharm Sci* **2015**, 18, 184.

[2] a)C. Tardy, M. Goffinet, N. Boubekeur, R. Ackermann, G. Sy, A. Bluteau, G. Cholez, C. Keyserling, N. Lalwani, J. F. Paolini, J. L. Dasseux, R. Barbaras, R. Baron, *Atherosclerosis* **2014**, 232, 110; b)S. Diditchenko, A. Gille, I. Pragst, D. Stadler, M. Waelchli, R. Hamilton, A. Leis, S. D. Wright, *Arterioscler Thromb Vasc Biol* **2013**, 33, 2202.

[3] a)W. Wang, X. Xu, Y. Song, L. Lan, J. Wang, X. Xu, Y. Du, *Asian J Pharm Sci* **2023**, 18, 100846; b)H. Yu, F. Jin, D. Liu, G. Shu, X. Wang, J. Qi, M. Sun, P. Yang, S. Jiang, X. Ying, Y. Du, *Theranostics* **2020**, 10, 2342.
